# Differential impact of environmental factors on systemic and localized autoimmunity

**DOI:** 10.3389/fimmu.2023.1147447

**Published:** 2023-05-22

**Authors:** Hanane Touil, Kristin Mounts, Philip Lawrence De Jager

**Affiliations:** ^1^ Center for Translational and Computational Neuroimmunology, Department of Neurology, Columbia University Irving Medical Center, New York, NY, United States; ^2^ Columbia Multiple Sclerosis Center, Department of Neurology, Columbia University Irving Medical Center, New York, NY, United States

**Keywords:** multiple sclerosis, systemic lupus erythematosus, Alopecia Areata, vitamin D, diet, therapeutic strategies

## Abstract

The influence of environmental factors on the development of autoimmune disease is being broadly investigated to better understand the multifactorial nature of autoimmune pathogenesis and to identify potential areas of intervention. Areas of particular interest include the influence of lifestyle, nutrition, and vitamin deficiencies on autoimmunity and chronic inflammation. In this review, we discuss how particular lifestyles and dietary patterns may contribute to or modulate autoimmunity. We explored this concept through a spectrum of several autoimmune diseases including Multiple Sclerosis (MS), Systemic Lupus Erythematosus (SLE) and Alopecia Areata (AA) affecting the central nervous system, whole body, and the hair follicles, respectively. A clear commonality between the autoimmune conditions of interest here is low Vitamin D, a well-researched hormone in the context of autoimmunity with pleiotropic immunomodulatory and anti-inflammatory effects. While low levels are often correlated with disease activity and progression in MS and AA, the relationship is less clear in SLE. Despite strong associations with autoimmunity, we lack conclusive evidence which elucidates its role in contributing to pathogenesis or simply as a result of chronic inflammation. In a similar vein, other vitamins impacting the development and course of these diseases are explored in this review, and overall diet and lifestyle. Recent work exploring the effects of dietary interventions on MS showed that a balanced diet was linked to improvement in clinical parameters, comorbid conditions, and overall quality of life for patients. In patients with MS, SLE and AA, certain diets and supplements are linked to lower incidence and improved symptoms. Conversely, obesity during adolescence was linked with higher incidence of MS while in SLE it was associated with organ damage. Autoimmunity is thought to emerge from the complex interplay between environmental factors and genetic background. Although the scope of this review focuses on environmental factors, it is imperative to elaborate the interaction between genetic susceptibility and environment due to the multifactorial origin of these disease. Here, we offer a comprehensive review about the influence of recent environmental and lifestyle factors on these autoimmune diseases and potential translation into therapeutic interventions.

## 1 Introduction

**Figure 1:**
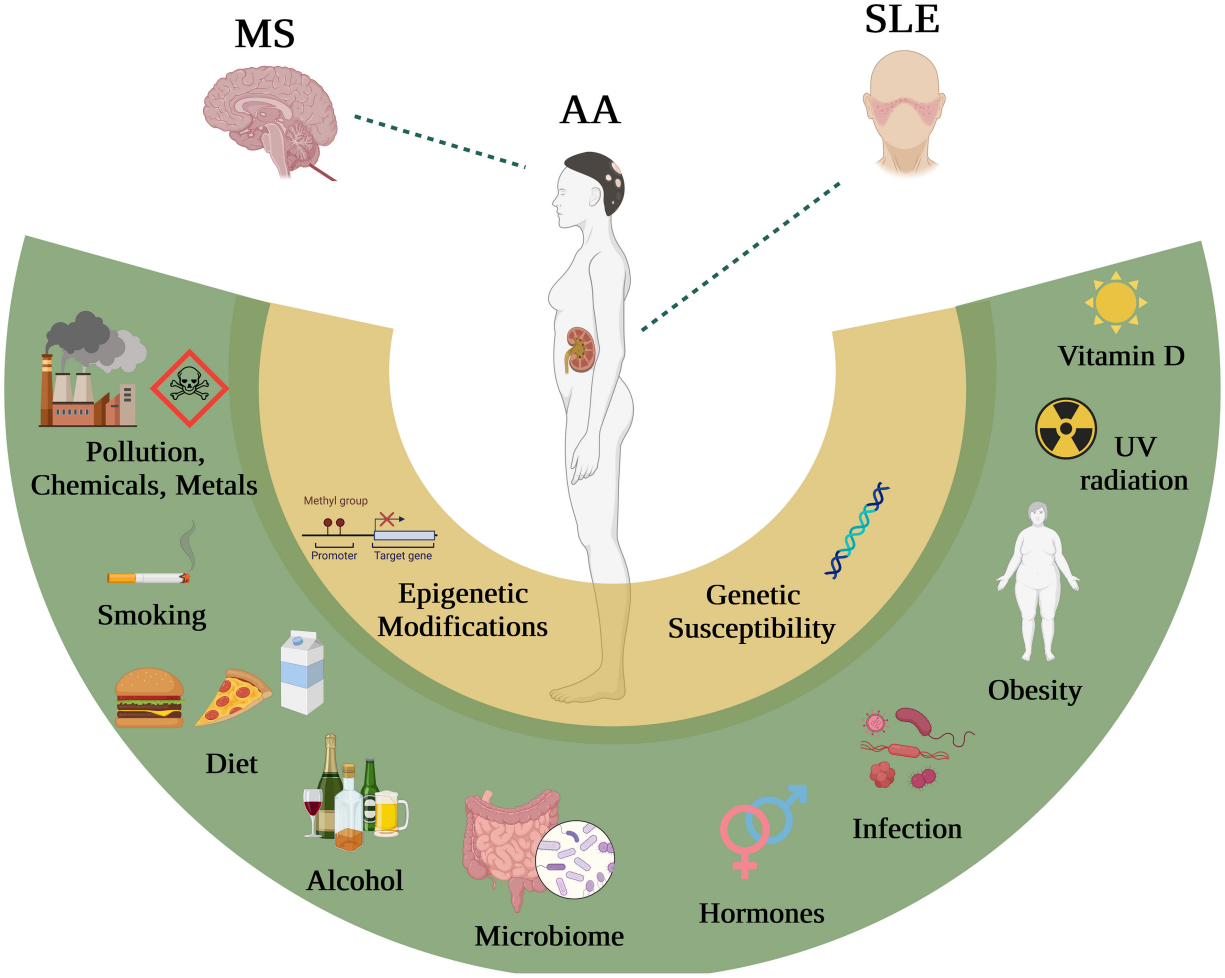
The influence of distinct environmental factors on MS, AA and SLE. Environmental factors including Vitamin D, obesity, viral infections, hormones, microbiome, alcohol, diet, smoking and chemicals, have all been shown (with some extend of controversy) to interact with genes in patients with MS, SLE and AA.

Autoimmune diseases occur when the immune system fails to distinguish self from foreign, leading to aberrant immune responses to self and qualified as ‘self-reactivity’. Overall, the prevalence of autoimmunity is around 9% of the population, and it affects higher proportions of females compared to males. Autoreactivity can be directed against specific organs such as the brain in Multiple Sclerosis (MS), the skin in Alopecia Areata (AA), or against systems such as Systemic Lupus Erythematosus (SLE), which are the focus of this review due to their broad coverage of the diverse types of autoimmunity. Several FDA approved disease-modifying therapies (DMTs) are already being used to treat MS (1), but limited therapies exist for SLE and AA. Thus, effective future therapies for SLE and AA remain an unmet need and should be designed to intervene prior to disease onset as growing evidence suggests that the initial pathogenic events take place prior clinical manifestations. MS, SLE and AA present clinical heterogeneity, while their polygenic nature suggests a multifactorial causal effect. Genetic predisposition is thought to be a key factor of increased autoimmunity, although it is now clear that the complex interplay between environmental factors and genetic susceptibility triggers disease onset. Several nucleotide polymorphisms (SNPs) were identified using genome-wide association studies (GWAS) for MS (2), SLE (3), and AA (4). Each SNP alone confers a modest risk to develop autoimmunity, as opposed to cumulative risk variance associated with higher disease prevalence. With the advent of big data and large consortiums including more elaborate technical and analytical tools (single cell-RNA-sequencing, CITE-Sequencing and causal-gene analytic tools), the genetics field evolved towards identifying potential causal genes. Nonetheless, the elucidation of causal genes alone would not suffice to guide effective therapeutic interventions, as careful consideration of environmental factors associated with autoimmunity is needed. Epidemiological data suggest a panoply of environmental factors associated with MS, SLE and AA including the lack of vitamin D, obesity, prior viral infections, lifestyle such as smoking, exercising and consuming alcohol (**Figure 1**). Nonetheless, epidemiological studies are susceptible to higher systematic errors due to the differential classification of lifestyles, and biased interpretations of causation/association reports. Since extensive reviews have already focused on the factors including Epstein-Barr virus (EBV) infection (5), consuming coffee, tobacco, and alcohol, here we highlight other risk factors with some conflicting findings including vitamin D deficiency, obesity, and diet (**Table 1**). Moreover, we will discuss opportunities on how to best leverage existing knowledge in the field of MS and benefit the fields of SLE and AA. We will also discuss strategies to integrate genetic risk factors with findings about environmental risk factors to help predict disease onset and progression for an efficient therapeutic intervention.

**Table 1:** Impact of environmental factors on MS, SLE and AA.

Environmental factors	Multiple Sclerosis (MS)	Systemic Lupus Erythematosus (SLE)	Alopecia Areata (AA)
Vitamin D (Vit D)	Deficient (<20ng/mL), or insufficient (21-29ng/mL) Vit D levels are reported in MS (6) Higher levels of ultraviolet B (UVB) irradiation are strongly associated with the prevalence of MS (r=-0.80, p<10^-5^) (7) Association between rs731236 (Taq-1) gene polymorphism within vitamin D Receptor (VDR) with MS (8)	SLE genetic susceptibility (CYP24A1 gene allele) (9) Deficient Vit D levels (~21.6ng/mL) (10) Low Vit D levels as a result of sunlight avoidance due to photosensitivity/renal insufficiency (11, 12)	Low serum levels of Vit D (14.03 ± 8.09 ng/mL) in AA, which represents 79.6% of patients with low Vit D compared to controls (13) Lower serum (9.99 ± 1.69 ng/mL) and tissue (199.7 ± 33.38ng/mL) Vit D receptor (VDR) in AA (14)
Obesity	˜3% weight increase in MS cases (n=1571) compared to controls (n=3371) (15) Significant 1.61-1.95 fold increased risk of MS associated with higher body mass index (BMI) in a cohort of (n=302,043) children (16) Adult GWAS data (n=32,105) identified 70 distinct SNPs related to genetically high BMI as a risk to develop MS (17) Expansion of adipocytes, a source of pro-inflammatory cytokines IL-6, TNF-α, in turn, help recruit immune cell infiltrates (18, 19) High resistin levels correlate with higher BMI, EDSS and IL-1β and TNF-α (20, 21)	85% significant increase risk to develop SLE in obese women compared to those with healthy BMI (22) Obesity at age 18 is associated with double SLE incidence during adulthood (HR 2.38, 95% CI:1,26-4.51) (23) Increased pro-inflammatory markers IL-6, IL-23, TNF-α & C-reactive protein (CRP) (24) known to be elevated in SLE and produced by adipose tissue. SLE patients harbor elevated serum resistin levels (p<0.001) that correlate with renal dysfunction and proinflammatory markers (25)	Higher BMI (p=0.005) in a Japanese AA cohort (n=70) compared to controls (n=70) (26) Higher BMI correlates positively with alopecia disease severity in males (n=189, p=0.01) (27) Lower serum levels of adiponectin (p=0.031) and resistin (p=0.017) with AA (n=65) compared to controls (n=71) (28) Negative correlation between adiponectin serum levels and AA disease severity p<0.05 (28)
Diet	Low iron (p=0.04), magnesium (p<0.001) levels in MS patients (9, 29) High sodium (40nM) found in western diet promotes *in vitro* Th17 differentiation (30) Mediterranean diet reduces inflammation through phenols and protect CNS from oxidative stress (31)	Inadequate intakes of iodine, potassium, magnesium, folate, and vitamins E and D, and overconsumption of sugar, sodium, and phosphorus (32, 33) High fiber intake by SLE patients may prevent disease activity (34) Vegetarian/pesco-vegetarian diets is associated with lower odds of SLE (35)	Protein deficiency (intake <30g/day) is associated with AA disease onset (28) Deficiencies in biotin (B7) (<100ng/L) and folate (B9) (110.62-243.75 ng/mL/cells) are associated with hair loss and AA (36, 37) Iron deficiency was reported in 56% of AA patients (<40ng/mL) compared to controls (38) Beneficial role of Mediterranean diet rich in fruits, vegetables (OR 0.43; 95% CI 0.21-0.89) in AA (n=104) compared to controls (39, 40)

## 2 Multiple Sclerosis (MS)

### 2.1 Multiple sclerosis pathogenesis

**Figure 2:**
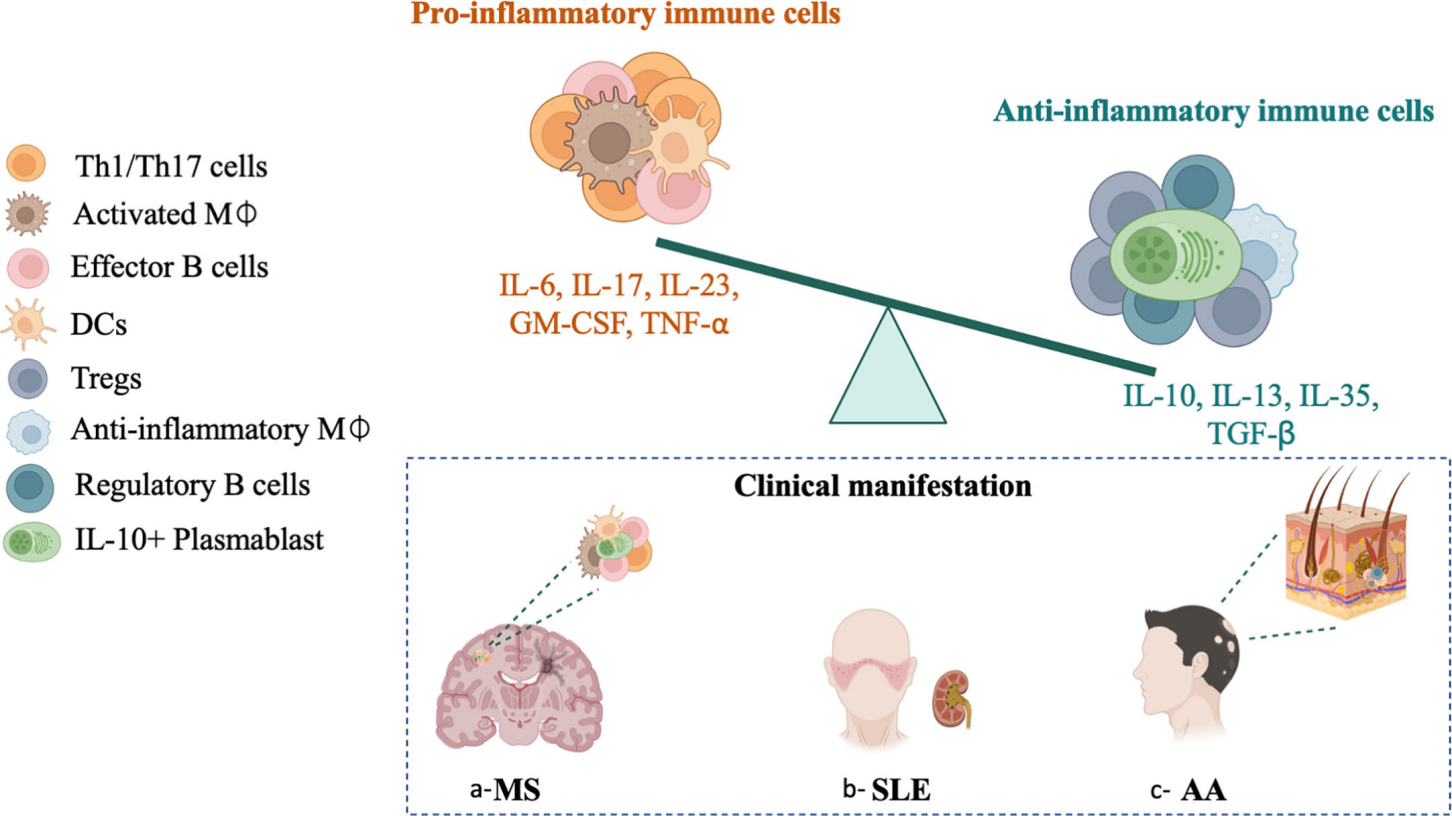
Altered balance between pro-inflammatory and regulatory immune cells associated MS, SLE and AA. Increased frequencies of pro-inflammatory Th1/Th17 cells (IL-17+, IL-23+), effector B cells (IL-6+, TNF-α+, GM-CSF+), pro-inflammatory macrophage (GM-CSF+, IL-6+, IL-23+), and lack or dysfunctional anti-inflammatory regulatory T cells Tregs (IL-10+, IL-35+, TGFβ+), regulatory B cells Breg (IL-10+, IL-35+), anti-inflammatory plasmablasts (IL-10+) and anti-inflammatory macrophage (IL-13+, IL-10+ and TGF-β+). The altered balance between pro-inflammatory and regulatory profiles in MS, SLE and AA might be a result of an over activation of the immune system, or an insufficient regulation.

Multiple sclerosis (MS) is a chronic autoimmune disease targeting the central nervous system (CNS) and affects a ratio of 4:1 females to males (41). Although it is a debilitating chronic condition, the past few decades brought considerable progress for MS patients. Notably, the increased number of available DMTs options for relapsing remitting form of MS (RRMS) (1) and more recently for patients with progressive MS (PMS) (42, 43). RRMS is thought to be driven by infiltrating peripheral immune cells causing peri-vascular injury within the CNS; the progressive form (PMS) remains not well understood and is thought to involve CNS-compartmentalized inflammation. Although RRMS/PMS are biologically distinct, the current consensus views them within the same spectrum and sharing subclinical biologic processes that overlaps for years prior to clinical manifestations. The latter evidence suggests an earlier influence of the environmental factors on individuals’ prior disease onset and clinical manifestations. Furthermore, environmental factors are thought to interact with the genetic background during the preclinical stage leading to MS disease onset. Yet specific factors triggering MS or contributing to relapse episodes and disease progression remain a mystery. At the cellular level, MS represents an abnormal balance between effector and regulatory T cells, including aberrant pro-inflammatory functions of IFN-**γ** ^+^ TNF-α^+^ CXCR3^+^ Tbet^+^ Th1, IL-17^+^ CCR6^+^ CD161^+^ ROR**γ**t**^+^** Th17 and GM-CSF^+^ T cells (44), while IL10^+^ CD25^high^ CD127^-^ FOXP3^+^ Tregs present deficient functions. While persistence of plasmablasts and increased immunoglobulin synthesis is a well-recognized feature of MS (45), growing evidence suggest that antibody independent functions of B cells are associated with new disease activity as supported by anti-CD20 therapies (43, 46, 47). B cells derived from MS patients secrete high levels of pro-inflammatory cytokines (TNF-α, Lymphotoxin-a, IL-6 and GM-CSF) (48, 49), but have a diminished capability to produce IL-10 (48, 50) (**Figure 2**). MS is thought to be more prevalent in Western countries and increases further away from the equator with lower exposure to sunlight, however such notions were recently challenged based on migration studies suggesting that MS disease onset might have taken place prior to migration towards countries away from the equator (51). Recent epidemiology data gathered by the Multiple Sclerosis International Federation found within the open source of the Atlas of MS (www.atlasofms.org), suggest that the overall worldwide MS numbers increased to 2.8 million patients in 2020; a 30% rise compared to 2013 (41). Additionally, higher numbers were reported in regions closer to the equator such as North Africa and the Middle East (52). It remains challenging to dissect the triggering cause leading to MS, while the genetic susceptibility alone cannot explain the recent increase in MS. Herein, we discuss environmental key factors associated with MS.

### 2.2 Influence of environmental factors during the lifespan

Onset of MS disease in pediatric populations is now recognized worldwide (53, 54). McDonalds criteria specific for pediatric patients were published in 2018 (55). Together with observational migration studies (56), it suggests that the risk exposure to environmental factors may occur prior 15 years-old as Rotstein D L et al. (57), demonstrated a greater MS risk in individuals who migrated at a younger age than 15 years old to Canada (Hazard Ratio (HR) 0.73, 95% confidence interval (CI) 0.63 – 0.85), and perhaps as early as the gestation period *in utero* and in neonates (58–60). Indeed, individuals who migrated from a low to a high-risk country before adolescence have higher risks of developing MS compared to the general population of high-risk countries (HR 1.72, 95% CI 1.00-2.75, p=0.049) (61). Although contributions and timing of environmental exposures promoting disease onset remain unclear, some evidence suggests that the exposure to environmental factors during pregnancy or early-life may be associated with disease onset. Maternal illness during pregnancy was associated with 2.3-fold increase to develop MS (95% CI 1.20-4.21, p=0.01) and is considered a risk factor for pediatric MS onset, while cesarean delivery appeared protective as it reduced the MS risk by 60% (95% CI 0.20-0.82, p=0.01) based on a large American case-control study (n=265 MS cases, and n=412 controls) (62). Which is contradictory with a Danish (n=930 MS cases) (63) and an Iranian (n=449 MS cases, n=900 controls) (64) cesarean case-controlled studies demonstrating that the latter had no effect on the risk of developing MS (OR=2.51; 95% CI 1.43-4.41; p=0.001 and RR = 1.77; 0.92-1.46), respectively. Moreover, having worked in a gardening-related occupation and exposure to pesticides from 3 months pre-pregnancy through the first year of life increases the risk of pediatric MS (OR 2.18, 95% CI 1.14-4.16, p=0.02 and OR 1.73, 95%, CI 1.06-2.81, p=0.03), respectively (62). While exposure to adhesives or paint thinners petroleum products after the first year of life was associated with a two-fold higher MS risk (OR 1.22, 95% CI 1.23-3.29, p<0.01) (62). In a cohort of (n=6649) babies born in the post-winter season appear to be more prone (11% higher risk) to develop MS (p=0.045) (65) compared to babies born between April and May, which may be due to residual confounding factors (66, 67). Yet, biological mechanisms behind those observations remain unclear and need more attention. The predominance of sex dimorphism in MS is stronger between puberty and menopause, while before/or after this time period the sex ratio is 1:1 earlier age. An earlier age at puberty tends to be associated with increased risks of developing MS (OR 0.56, 95% CI 0.33-0.69; p=0.035) (68), and peaks two years post-puberty (69). Although it is unclear whether this is due to the direct impact of hormonal changes, past infections, or other factors.

### 2.3 Vitamin D

Vitamin D deficiency has been recognized as a risk factor for MS since the 1970s, leading to considerable efforts to understand how clinical intervention using vitamin D supplementation throughout the disease course may prevent or alter MS pathology (70). Regardless of their geographical localization, MS patients exhibit a deficiency in vitamin D (<20ng/mL) or insufficient levels (21-29ng/mL) (6). Deficient/insufficient vitamin D levels are generally associated with a low sunlight exposure, although MS patients from sunny countries such as southern Italy (71) and Australia (41) also display low vitamin D levels, which suggests deficiencies might be attributed to low levels of active vitamin D, or perhaps a lack of availability of the vitamin D receptor (VDR). To exert its direct anti-inflammatory property, vitamin D must bind to VDR, forming VDR-D complex that further binds to the receptor RXR that is activated by the retinoic acid (RA) metabolite found in vitamin A (71). This process indicates that supplementation with vitamin D and A in MS patients might improve MS by triggering an anti-inflammatory cascade suggested by the increase of IL-10^+^ CD4 T cells, and decreased ratio of IFN-**γ** ^+^/IL-14^+^ T cells (72, 73). In addition, active vitamin D has immunomodulatory effects by suppressing the innate and the adaptive immune system (74). Its effect could be mediated directly through the VDR signaling pathway, or indirectly through the antigen presenting cells (APCs). Vitamin D3 isoforms were reported to modulate the balance between the pro-inflammatory and anti-inflammatory cytokines, known to be altered in MS. In humans, 1,25 dihydroxyvitamin D3 (1,25(OH)2D3) induces the differentiation of IL-4 secreting T cells through the induction the transcriptomic factor GATA-3, while inhibiting the pro-inflammatory Th1 cells by blocking the secretion of IFN-γ (74, 75), which in turn, induces the cytotoxic and proliferation of both CD4^+^ and CD8^+^ T cells. Thus far, despite the conflicting data about the impact of vitamin D3 on B cells, the most robust data is in support of an inhibition of B cell proliferation and differentiation into plasma cells, resulting in reduced levels of immunoglobulin G (IgG) (74, 76, 77). 1,25(OH)2D3 seems to also affect the innate immune system, by increasing the differentiation of NK cells from the hematopoietic stem cells *via* VDUP1 (49, 78). The important roles of 1,25(OH)2D3 within the CNS include its secretion by neurons and microglia, which modulates neurotrophic factors secretion enabling calcium influx into neurons through L-type calcium channels (79, 80).

### 2.4 Obesity

Obesity is considered an MS risk factor, while most recent findings implicate obesity as a contributor to MS pathophysiology. During childhood, risk of developing MS seems lower than in adolescence where it doubles (16, 81). Interestingly, adult obesity did not influence the risk of MS diagnosis (82). A large cohort based comprehensive study (n=1571 patients, n=3371 controls), amongst others, revealed a two-fold weight increase (3% on average) in MS cases compared to controls (OR 2.2 95% CI 1.6-3.0; p=1x10^-6^) (15). In addition, a prospective Danish investigation where they examined the body mass index (BMI) of 300,000 students revealed a significantly increased risk of MS in girls aged between 7-13 who were ≥95^th^ percentile of BMI (1.61-1.95-fold increase) and boys with a BMI ≥95^th^ percentile at age 7 (1.81-fold increase) (16). Mendelian randomization investigations demonstrated that high BMI is a genetic determinant that is strongly associated with increased risk of developing MS. A large BMI based genome-wide associated studies (GWAS) (n=322,105) in adults identified 70 distinct SNPs including SNPs of genetically elevated BMI as a risk to develop MS (17). Whereas pediatric mendelian randomization (MR) studies reported 11 overlapping/correlating BMI SNPs with the adult studies (p = 0.01) (83), which reinforces the idea that predisposition to genetically elevated BMI may be a causal factor in MS disease onset. Moreover, childhood obesity was significantly associated with a higher risk of pediatric-onset of MS and with clinically isolated syndrome (CIS) in girls (p=0.005 for trend) but not boys (p=0.93) (84). Specifically, extremely obese girls had over three times higher chances of developing the disease compared to children with a healthy weight, as the adjusted odds ratio and 95% CI for CIS/MS overweight girls was 1.58 (0.71-3.5) compared to healthy weight category (84). Obesity is accompanied with self-directed tissue inflammation whereby adipocytes are subjected to considerable expansion to be able to store lipids (85). Adipose tissue itself and the immune cell infiltrates through their ability to secrete pro-inflammatory cytokines are inflammation sources. In fact, fat cells are known to physiologically secrete hormones (leptin, adiponectin and resistin) and pro-inflammatory cytokines including IL-6 and TNF-α, which are recognized to be elevated during obesity, and in turn stimulate the recruitment of immune cell infiltrates (19, 86). To date, there is very little evidence regarding the specific role of adipose tissue in MS pathogenesis, and how these cells interact with the immune system contributing to relapses/disease progression.

At the cellular level, obesity is accompanied by a shift towards pro-inflammatory T cells (Th1/Th17) (87, 88) to the expense of the anti-inflammatory Th2 cells (88). More specifically, IL-17 and IL-23 producing Th17 cells frequency increases with obesity (89), which is accompanied by an altered IL-10 producing Treg frequency in adipose tissue (90). The latter immunophenotypic profiles are also reported in MS, and perhaps amplified in obese MS patients leading to an over activation of the immune system and a lack of regulation, as reviewed by Correlale et al. (91). The adiponectin hormone secreted by adipose tissue was found to be significantly abundant within the serum of children with MS (n=43, p<0.005) and appears to induce pro-inflammatory states of CD14^+^ monocytes through increased expression of CD80, CD86, TNF-α and IL-6, as well as the adaptive immune cells IFN-γ producing CD4^+^ and CD8^+^ T cells directly or *via* myeloid cells, and altering the quiescence profile of human microglia (92). Furthermore, adiponectin levels in the CSF and serum of MS patients appear to correlate with MS disease severity and progression and are higher compared to controls (93–96). Nonetheless, such observations remain subject to debate due to the lack of significant correlation between adiponectin levels and MS disease activity (93). Another hormone of interest is resistin, found within the CNS and significantly higher levels were found in the periphery (serum) of MS patients which positively correlates with a higher BMI and EDSS, as well as pro-inflammatory cytokines IL-1β and TNF-α (20, 21). High resistin levels correlate with lower regulatory T lymphocytes (Treg) in RRMS (97), although it remains unclear whether resistin directly exerts suppressive effects on Tregs.

### 2.5 Diet

The advent of industrial development revamped our lifestyles on many levels, including our diet, which has been linked to an increased prevalence of autoimmune conditions. Increased attention emerged over the influence dietary habits on MS disease onset and course, although how specific nutrients impact the immune system, and the interaction between CNS and immune cells, remains unclear. Compared to controls (n=146), MS patients (n=63) have lower levels of iron (p=0.04) (29, 98), while magnesium deficiency represent a risk factor for MS although the sample size was limited Yasui et al., report lower magnesium levels within post-mortem CNS tissue from MS patients (p<0.001) compared to controls (99). Cross-sectional MS-studies (n=69) indicated that 85.5% of patients did not meet nutrition guidelines (100). Despite the sparse data regarding dietary intervention to help patients with MS, several diets have been suggested to influence the MS disease course. Mediterranean-style diets are known to be low in saturated fats, while being high in polyunsaturated and monounsaturated fats and consist of a high intake of fruits, vegetables, grains. Fish, dairy products, and red meat are moderately consumed. Overall, the Mediterranean diet is thought to reduce inflammation in part due to phenols present in olive oil exerting anti-inflammatory properties that protect the CNS from oxidative stress, reported in MS (31, 101, 102). In contrast, the western diet is known to be enriched in processed food, high in saturated fats, sugar and salt and overall poor in whole grains, fruits, and vegetables and appears to negatively impact MS disease course (103). In an experimental autoimmune encephalomyelitis (EAE) animal model, mice fed with a western high-fat diet exhibited high T cell and macrophage infiltration, higher IL-6 and IFN-γ levels and worse clinical scores (104).

#### 2.5.1 Unsaturated fats

Polyunsaturated fatty acids (PUFAs) or monounsaturated (MSFA) fats are found in fish, flax seeds, walnuts, avocados, olive oil, nuts such as almonds, walnuts and peanuts. Typically, polyunsaturated fats like omega-3 and omega-6 fatty acids down-regulate inflammation. A recent study led by Dr. Kappos L demonstrated that PUFAs measured in the serum is in immune regulation, decreasing the risk of conversion from clinically isolated syndrome (CIS) to MS, as well as decreasing the risk of relapse (105). Observational studies utilizing a large cohort of over 90,000 women nurses over two distinct periods of time established a significantly better outcome for MS in response to a diet enriched in PUFAs (106). However, the mechanism by which unsaturated fatty acids reduce relapse rates, or influence conversion from CIS to MS remains to be discovered. At the cellular level, Omega-3 fatty acids modulate macrophages, neutrophils, T cells, B cells, NK cells and DCs, although their precise mode of action on immune cells in MS is yet to be discovered. *In vitro*, omega-3 fatty acids have been shown to increase macrophage phagocytic functions, perhaps through driving the anti-inflammatory phenotype of macrophages known to be phagocytosis competent phenotype (107, 108). Oxidative stress (OS) increases demyelination during MS, while *in vitro* it is diminished in response to Omega-3 treatment through the suppression of the pro-inflammatory profile of macrophages (lower IL-6 & TNF-α cytokine secretion) (109). Finally, omega-3 fatty acid inhibits the polarization towards pro-inflammatory Th1/Th17 cells (110), and promotes Tregs differentiation (110).

#### 2.5.2 Saturated fats

Western diets have elevated levels of saturated fats, known to increase LDL cholesterol are associated with disability and high MRI activity in MS (111, 112). At the cellular levels, saturated fatty acids have been reported to directly activate the pro-inflammatory signaling pathway through TLR-2 and TLR4 signaling pathways (112, 113). Both TLR-2 and TLR-4 are known to be involved in MS pathophysiology. TLR-2 is expressed by MS Tregs at a higher level compared to matching controls, and upon activation T cells shift towards pro-inflammatory Th17 cells (114), while TLR-4 upon activation leads to the NF*k*B pathway activation inducing pro-inflammatory cytokines (IL-6. IL-23 & IFN-γ), promoting T cell proliferation and survival in MS (115, 116).

#### 2.5.3 Grains, fruits and vegetables

A healthy diet is recommended for the general population as well as patients with autoimmune conditions, although little evidence exists to define the direct impact of fruits, vegetables, and grains on the immune system. In MS, a higher intake of fruits and vegetables is associated with lower disease activity based on the Health Outcomes and Lifestyle In a Sample of people with Multiple Sclerosis (HOLISM) study (n=2047), that consisted of a dietary questionnaire directed to MS patients (117). Moreover, pediatric MS patients demonstrated a decreased relapse rate in response to a higher consumption of vegetables (118). Recent findings deploying the north American research committee on MS (NAARCOMS) registry questionnaire suggest that high-quality diet enriched in fruits, vegetables, whole grains and low in sugars and red meat is associated with a better disease outcome, including lower disability rates in MS patients (119).

#### 2.5.4 Dairy

Based on the HOLISM study, a low dairy consumption diet is associated with lower disease activity and improved quality of life compared to MS patients that consume dairy products (117). Nonetheless, it may result in a decreased calcium consumption known to be low in MS. Although survey studies do not directly assess the precise mode of action of dairy derivates on immune cells, it opens new avenues for mechanistic based research.

#### 2.5.5 Sodium

The Western diet is enriched in salt. A decade ago, several groups closely examined the impact of higher sodium chloride (NaCl) concentrations similar to those found in the mice intestine and demonstrated that not only it induces the differentiation of T cells into pro-inflammatory Th17 cells, but exacerbates EAE (30, 120, 121). In MS, Th17 are recognized to be pathogenic T cells, and Kleinewirtfeld et al., further showed that *in vitro* 40nM of NaCl promotes the pro-inflammatory pathogenic Th17 IL-17^+^, through the phosphorylation of p38 mitogen-activated protein kinase (MAPK) and the nuclear factor of activated T cells 5 (NFAT 5) (120). Despite direct *in vivo* and *in vitro* evidence of the impact of NaCl on Th17 cells, it remains crucial to test NaCl concentrations comparable to those found in the human intestine, as well as using human blood/CSF samples from strictly controlled diet groups of patients (high salt diet, lower salt).

### 2.6 Dietary effects on the gut microbiome

Different diets have the potential to influence the composition and function of the gut microbiota distinctly, hence it is crucial to pay close attention to these interactions to help better control disease activity and prevent CNS autoimmunity through healthy diets. To date, there is no consistent evidence of a clear MS microbiome phenotype, but a panoply of microbial species has been described. Initial investigations shedding light on the importance of the gut microbiota in MS, constitute the surprising benefits of fecal microbial transplantation (FMT) in MS, and suggested as a therapeutic strategy (122). Recently, a report by the international MS Microbiome Study (iMSMS) based on 576 patients and 1,152 controls, highlighted that specific gut microbiome signatures as a risk for MS and may impact disease progression, while defined DMTs could shape functional profile of the gut microbiome (123). Significantly increased proportions of Akkermansia muciniphila (FDR<0.05), Ruthenibacterium lactatiformans (FDR<0.01), Hungatella hathewayi (FDR<0.001), and Eisen-bergiella tayi (FDR<0.001) and decreased Faecalibacterium prausnitzii (FDR<0.01) and Blautia (FDR<0.05) species were reported in MS (n=209), compared to controls (123). A vegetarian diet enriched in non-fermentable fiber in early life prevents CNS autoimmunity by altering the composition of gut microbiota and increasing long-chain fatty acids that support suppressive Th2 cells functions (124). Finally, recent focused investigations link IgA^+^ producing B cells to a specific gut microbial immune response, which constitutes an important gut-brain axis as they are recruited from the gut to the inflamed MS CNS during relapses and exert regulatory properties through IL-10 secretion (125, 126).

## 3 Systemic Lupus Erythematosus (SLE)

### 3.1 Pathogenesis

Systemic lupus erythematosus (SLE) is a multisystem autoimmune disease which can affect the joints, skin, nervous system, lungs, kidneys, and blood vessels. Its vast clinical heterogeneity compelled the development of 11 clinical and immunological criteria that are used in formal diagnosis (127, 128). The development of SLE is multifactorial, involving an innate susceptibility that interacts with epigenetic, environmental, lifestyle, and hormonal factors over an individual’s lifetime, which amplify an underlying dysregulated innate and adaptive immune response and trigger disease onset (129, 130). Aberrant innate and adaptive immune responses are thought to contribute tissue injury in SLE (131). Autoreactive B cells differentiate into pathogenic memory and plasma cells through germinal center responses, giving rise to increased autoantibody titers (132, 133). Active SLE is associated with naive CD19^+^CD27^-^ B cell lymphopenia, while transitional CD19^+^CD24^hi^CD38^hi^ B cells, switched memory CD19^+^CD27^+^IgD^-^ B cells, double negative CD19^+^CD27^-^IgD^-^ B cells, plasmablasts/plasma cells CD27^hi^CD38^+^CD19^+^sIg^low^CD20^-^CD138^+^ B cells are increased which correlate with SLE disease activity (134, 135). IL-10 producing transitional B cells suppress pro-inflammatory Th1 and Th17 cell differentiation, although they are impaired in SLE (136). In SLE, double negative CD4^-^CD8^-^ T cells are expanded, and reported to infiltrate kidneys and produce higher levels of IL-1 β and IL-17 (135). Cytotoxic CD8 functions are reportedly altered in SLE (137), while frequencies of CD4^+^Foxp3^+^ Tregs are low and present altered regulatory functions (138). While SLE’s exact pathogenesis is complex and unknown, likely due to its immense heterogeneity in clinical and molecular phenotypes (139), the breakdown of self-tolerance and sustained production of autoantibodies are central tenets in our understanding of SLE (129). However, what drives the sustained loss of self-tolerance and spread systemically is unknown. In the current model, genetic and environmental triggers are well-researched initiators of SLE. Genetic studies have identified many susceptibility loci, including alleles in the MHC, interferon, complement pathways, and many others in both the innate and adaptive immune system (3, 140, 141). Some genes relate to age of onset and clinical course, with what appears to be a stronger genetic component and more severe manifestations in pediatric SLE cases and what may be a more nuanced interplay between genetic and environmental factors in adults (141–143).

### 3.2 Influence of environmental factors during the lifespan

Almost 60% of SLE risk may be related to environmental exposures and gene-environment interactions (144), which accumulate over the lifespan. Preterm delivery (OR 28.9, 95% CI 2.9–287.8, p=.004) and exposure to inhalable particles or volatile components (OR 7.4, 95% CI 1.3–42.3, p=.03), secondhand smoke during pregnancy and after birth (OR 9.1, 95% CI 1.8–45.2, p=.007), and low-to-middle socioeconomic status (OR 2.8, 95% CI 0.5–16.6, p=.26) have been associated with an increased risk of childhood-onset SLE using multiple logistic regression models (145). Like adult-onset SLE, other environmental exposures associated with childhood-onset SLE include UV light, drugs, and viral infections (146). Environmental exposures associated with SLE development are cigarette smoking, crystalline silica, alcohol, oral contraceptives and hormone replacement therapy, EBV, dietary factors, and occupational exposures including solvents, pesticides, mercury and trichloroethylene; more details can be found in a recent review here (147). Gene-environment interactions over the lifespan can be mediated epigenetically, as indicated by data on SLE discordance in monozygotic twins is associated with widespread changes in methylation pattern, enriched in immune function associated regions (148). DNA hypomethylation in SLE cells is a dominant pattern and driving force towards autoimmunity and severity (149). The plethora of different SLE-associated stimuli may act cumulatively over the lifespan and synergistically through epigenetic modifications that promote autoreactive T cells (149, 150). Environmental factors which may dysregulate DNA methylation and contribute to cells switching to an autoreactive state include diet, drugs (i.e. procainamide and hydralazine), oxidative stress-inducing agents like infections, UV light, smoking, mercury, and pollution (151–154). Increasing evidence attributes neutrophil extracellular traps (NETosis), a specialized form of neutrophil apoptosis, as major source of auto-antigen in SLE, leading to the release of DAMPs that activate immune responses (155, 156). Pathways which usually prevent immune activation to this endogenous cellular material may also be altered in SLE patients, such as the degradation of chromatin by DNase I (157, 158). Increased apoptosis can be caused by environmental factors such as UV exposure, infections, toxins, and drugs, all factors which are well-associated with SLE and flares (36, 130). Environmental factors associated with SLE can also drive inflammatory cytokines which initiate downstream pathological processes including hyperactive B and T cells, loss of tolerance, and autoantibody production (130). The subsequent formation, deposition, and inadequate clearance of immune complexes in tissues ultimately leads to organ damage.

There is a striking sex difference in SLE; 90% of patients are female and most patients are diagnosed during reproductive years when hormonal levels are high (159). There is scarcity of research into sex differences in environmental exposures and sensitivity to environmental factors in SLE incidence, which is remarkable considering that estrogens and endocrine disruptors may contribute to SLE development and flares in a dose-dependent manner (150, 160, 161). Endocrine disruptors have been associated with autoimmune disease as well as SLE, which ties into the importance of natural estrogens in immune regulation and dysregulation (150, 162). Recently, early-life exposure to pesticides was associated to increased SLE risk (OR = 2.3; 95%CI 1.3-4.1) in a dose-dependent manner (163). Pesticides are endocrine disruptors and increase oxidative stress. Both personal use of and work-related exposure to pesticides (OR 7.4, 95% CI 1.4-40.0) and insecticides (HR 1.97, 95% CI 1.20-3.23) have previously been associated with SLE risk and autoimmunity with a greater risk conferred by longer duration and increased usage (164–167). Exposure to pesticides is also associated with increased mortality from SLE (168).

All together, these studies indicate that environmental agents that increase oxidative stress, apoptosis, and inhibit DNA methylation can contribute to lupus onset and flares. Focusing in on these agents, diet is one of the most modifiable risk factors, and a feasible and accessible approach to making a positive impact on SLE patients, perhaps in an individualized way.

### 3.3 Vitamin D

Low levels of vitamin D are frequently observed in SLE patients (~21.6ng/mL) (10) (and obese patients) in multiple studies and it is not known whether deficiencies are a cause or result of the disease; however, low levels are often attributed to avoidance of sunlight due to photosensitivity or renal insufficiency in patients with nephritis (11, 12, 169, 170). Vitamin D and the its metabolism gene allele CYP24A1 is related to the risk of developing SLE, as shown by prospective study cohort of at-risk family members (n=436) who were assessed for vitamin D level at baseline and through follow-up, vitamin D supplementation, and genotyped for SNPs (9). The impact of vitamin D on SLE risk was modified by the number of minor alleles in CYP24A1. With two copies of the minor allele, having higher vitamin D levels was associated with a decreased SLE risk (OR 0.91, 95% CI 0.84-0.98), deficiencies were associated with an increased risk of transitioning to SLE (OR 4.9, 95% CI 1.33-18.04) (9). In individuals with deficient vitamin D levels and two minor alleles, the incidence of SLE development attributable to deficiency and two minor alleles was a striking 68.1%. As the enzyme product of CYP24A1 initiates Vitamin D degradation, this study indicates a role of genetics in the pathogenic effect of low Vitamin D on SLE development (9).

VDRs are expressed in many immune cell lines and activation leads to functional changes, generally skewing cells towards anti-inflammatory states (171). While VDR polymorphisms have been reported in SLE and are possibly implicated in both in vitamin D’s effect on immune cells and in vitamin D serum status, results have been inconsistent and conclusions cannot yet be made (171–174). Deficiencies are associated with altered immune cell differentiation and some studies have shown an association with increased SLE progression and activity, while others do not (11, 169). *In vitro*, vitamin D (10 nM) has been shown to decrease neutrophil extracellular traps (NETs) and prevent endothelial damage in cultured neutrophiles derived from SLE patients, compared to controls (p<0.05) (175). NETs are net-like fibrous structures on activated neutrophils which play an important role in fighting infections but are also associated with autoimmune diseases, possibly through a dysregulation between creation and degradation of NETs and subsequent activation of inflammatory cascades due to prolonged exposure to NETs. The therapeutic use and impact of vitamin D *in vivo* within SLE patients has been investigated in different studies which are discussed in more detail below (155, 156). In one prospective study, vitamin D supplementation in patients preferentially increased naïve CD4^+^T cells frequency (p<0.01) and CD3^+^CD4^+^CD25^hi^CD127^-^FoxP3^+^ Tregs (p<0.01), decreased effector IFN-g^+^Th1 (p<0.05) and IL-17A^+^Th17 cells (P<0.01), and decreased memory IgD^-^CD27^+^B cells (p<0.001) as well as anti-DNA antibodies (176).

### 3.4 Obesity

Obesity is strongly associated with inflammation and inflammatory arthritis. Studies estimate that about a third of SLE patients are overweight and/or obese, which is associated with impaired functional capacity (177). Adults patients with SLE recruited longitudinally part of the multi-ethnic Southern California Lupus Registry (SCOLR) indicated that increased BMI (n=130 obese patients) had severe SLE disease activity index score (SLEDAI) (p=0.0026) (178). Obesity independently initiates a proinflammatory state and has been associated with SLE development; excess weight in women disrupts hormones involved in immune function (178, 179) and obese women followed over 20 years had a higher risk of SLE (22). Obesity during adolescence increased the risk of SLE (95% CI 1.26–4.51) even further (180). Concerningly, if patients are obese during childhood, later achieving a normal body weight in adulthood does not appear to reverse the conferred risk (179). Obesity in SLE is associated with increased inflammatory markers including C-reactive protein (CRP), IL-6 (24), IL-23 (181) and TNF-α (182); IL-23 is associated with nephritis and a hypercoagulable state. Several studies and a meta-analysis have found increased adiponectin levels in lupus patients (183, 184), like the findings in MS as described above. Higher levels have been associated with severity of disease (183), but a meta-analysis found no relation to SLEDAI score (184). Adiponectin concentration was higher in obese SLE patients and those with plaques (p=0.033) (184, 185). It may be that higher levels of adiponectin mitigate the inflammatory response, as adiponectin is thought to have anti-inflammatory and protective endothelial effects (184, 186). Obese SLE patients are also at a higher risk for metabolic syndrome compared to non-obese SLE patients, a cluster of metabolic abnormalities including high blood pressure, dyslipidemia, and high glucose levels (187) that further perpetuating the chronic inflammatory state and oxidative stress. Overall, obese SLE patients have higher disease activity, poorer outcomes, and worsened organ damage which ultimately reinforces the great need for intervention (178, 188–190). Interventions may include dietary and lifestyle changes. Contributing factors in obesity and connections to SLE development include a high-fat diet, gut dysbiosis, medications, and physical hypoactivity. A high-fat diet can lead to weight gain and gut dysbiosis, which may lead to impaired immune regulation and tolerance to beneficial intestinal microbes (191, 192). Losing weight may be difficult for SLE patients, as the small number of studies exploring lifestyle-based dietary and exercise regimens have been mostly ineffective at promoting weight loss. However, weight loss was not a primary outcome in most and corticosteroid use may be a confounding factor (193). Nonetheless, one study did achieve significant weight loss in SLE patients who were on either a low-calorie or low-glycemic index diet, with a reduction in fatigue as well (194). Obesity can lead to sedentary behavior and vice-versa, which has important consequences for immune function. Physical activity such as Tai Chi Chuan promotes a healthy immune state by significantly increasing the ratio T helper to suppressor cells (CD4:CD8) (p=0.05), and increasing CD4^+^CD35^+^ Tregs producing TGF-β and IL10 known to suppress the immune system (195, 196).Thus, physical hypoactivity can exacerbate adipose-related systemic inflammation (197) and exercise may be an area of intervention for SLE patients. Exercise can promote cardiovascular health (198), increases insulin sensitivity (198), helps prevent bone mineral loss (199), and may help with fatigue and depressive symptoms (200), as well as ameliorating the effects of corticosteroids, including weight gain and muscle weakness (201).

### 3.5 Diet

Over the past two decades, researchers have been investigating diet, nutrition, and dietary-related microbiome changes as complementary approaches to understanding and treating SLE. Current literature supports the idea that a balanced diet and nutrients confer anti-inflammatory, immunomodulatory and antioxidant effects which may offer benefits to SLE patients (202, 203). Alarmingly, a cross-sectional study showed that the majority of normal weight SLE patients (67.2%) have an inadequate nutritional intake (90% less) (177). Most patients had an inadequate intake of calcium, iron, vitamin B12, and fiber, possibly due to a low consumption of fruits and vegetables. While, there was an overconsumption of fats and oils, contributing to dyslipidemia and cardiovascular risk. Other studies have shown similar results alongside inadequate intakes of iodine, potassium, magnesium, folate, and vitamins E and D, and overconsumption of sugar, sodium, and phosphorus (32, 33). A recent large, prospective study using the Nurses’ Health Study (NHS) and NHSII population (n=185,962) identified healthy diet, body weight, regular exercise, never smoking or past smoking, and moderate alcohol intake as modifiable risk factors associated with a decreased risk of SLE development HR 0.42 (95% CI 0.25-0.70) (204). Remarkably, SLE risk was halved for those with the highest adherence to healthy behaviors compared to the lowest. The effects were additive for each additional behavior, suggesting that these modifications may act synergistically *via* common underlying mechanisms. Importantly, while these underlying mechanisms remain to be elucidated, these results emphasize the potential for SLE prevention with lifestyle changes, including adherence to a healthy diet and body weight.

#### 3.5.1 Mediterranean diet

The Mediterranean diet has recently been shown to positively impact SLE disease activity (SLEDAI ≥5) (OR: 0.13; 95% CI: 0.04 – (−0.50), p<0.001), such as using more than 2 vegetable servings per day, significantly reduced SLE damage (SLICC/ACR Damage Index (SDI) ≥1)) (OR: 0.04; 95% CI: 0.005 – (–0.352), p<0.001), as well as reducing CRP by 24%, IL-6 by 16% (205) and homocysteine levels, and was associated with lower obesity and cardiovascular risk (206, 207). Possible mechanisms underlying its benefit include a reduction in overall inflammation, oxidative stress, and modulation of the immune system and DNA methylation status. Olive oil, fruits, vegetables, and fish may be the most beneficial components. Fruits and vegetables are important sources of fiber. A diet low in fiber (like the westernized diet) can lead to a state of gut dysbiosis that contributes to the development of autoimmunity and other health disorders (208, 209). Inflammation and immune dysregulation may result from gut microbiome perturbation in that alters its production of short-chain fatty acids (SCFAs), a product of fiber fermentation (210, 211). Indeed, SLE patients often have gut dysbiosis which has been linked to a low consumption of fiber (212). Higher fiber intake also increases gut motility, resulting in lower uptake of potentially harmful compounds from the diet (213, 214). In turn, a diet high in fiber consumed by SLE patients is associated with reduced disease activity (214).

#### 3.5.2 Protein restriction and vegetarian diet

A relative abstinence from meats may also independently show benefit for SLE patients, as suggested by a recent study using the Adventist Health Study-2 cohort (n=77,795) to investigate the relationship between self-reported dietary patterns and diagnosed SLE (35). Most participants were already vegetarians or pesco-vegetarians and this dietary pattern was associated with lower odds of SLE (OR=0.75, 95% CI 0.56-1.02), and (OR 0.88, 95% CI 0.57-1.36), respectively (35). Conversely, there was increased SLE diagnosis in nonvegetarians, which trended upwards with greater consumption of meat (35).

Protein restriction is commonly applied in kidney disease. In lupus nephropathy, high protein intake directly worsens renal filtration, while limiting protein slows the decline in kidney function (215). Excessive protein in diet (75% higher) has also been shown to accelerate bone mineral loss in juvenile SLE (216). However, a moderate intake of protein is recommended for most due to its nutritional importance; therefore, only in patients with overt kidney disease is an avoidance of high protein recommended currently. One way that amino acids can modulate the immune response through mechanistic target of rapamycin (mTOR), a paramount regulator and sensor of nutrient status, intracellular metabolism, oxidative stress, and immune response that is implicated in the development of autoimmunity (217). Amino acid metabolites, like kynurenine from tryptophane, can promote mTOR activation. mTOR contributes to T cell dysfunction, apoptosis, and reduced CD4^+^/CD8^+^ ratio in SLE (0.72 ± 0.12%) patients, as well as a significant increase of CD8^+^ T cells (p<0.001) due to upstream depleted glutathione levels and mitochondrial hyperpolarization (218). Percentages of CD4 and CD8 T cell apoptosis in SLE patients were higher than controls, p<0.001 and p<0.01, respectively (219).

#### 3.5.3 Fatty acids

Fatty acids and PUFAs positively impact SLE patients in most RCTs (220). Mammals are unable to synthesize these essential nutrients and may obtain them from dietary sources like fish, meat, nuts, corn, olive oil, soybeans, and vegetable oil. Clinical trials in SLE patients have shown anti-inflammatory benefits and suppression of pro-inflammatory pathways from increased consumption of PUFAs and especially omega-3 PUFA (221–224). Fish oil omega-3 supplementation has been shown to improve endothelial function measured with flow-mediated dilatation by 5.9% (p<0.001), energy/fatigue (p=0.092), emotional well-being (p=0.07), quality of life, increase serum Vitamin D (p=0.005), reduce inflammatory cytokines (IL-17 p=0.001, IL-1 p=0.003, IL-6 p=0.001) increase antioxidant enzyme activities like glutathione peroxidase and catalase, and reduce anti–dsDNA (224–226). In SLE mouse model NZBWF1, fish oil reduced expression of IL-6 and TNF-α in the kidneys (3-5 fold), while high-oleic and corn oil diets had increased expression of IL-6 and TNF-α (6- and 14-fold, respectively; 6- and 15-fold, respectively) Splenic osteopontin cytokine mRNA expression was attenuated in fish oil diets, while corn and high-oleic safflower diets showed a 7- to 8-fold increase. Other pro-inflammatory cytokines were attenuated in the fish oil diet, such as CCL-5 chemokine (4-7 fold) and CXCR3 (3 fold) chemokine receptor, compared to increased expression in high-oleic safflower and corn oil diets (11- and 15-fold) (221). This suggests that different sources of fatty acids may not be equally beneficial and may promote SLE manifestations instead of being protective (221). Lastly, the polyphenols contained in extra virgin olive oil (EVOO) have also shown to have a beneficial immunomodulatory effect in SLE patients, as shown by a reduction in CD4^+^CD69^+^ T cells p<0.001 after *in vitro* PHA (10 µg/mL) treatment, decreased IFN-γ (p<0.001), TNF-α (p<0.001), IL-6 (p<0.001), IL-1β (p<0.01), and IL-10 (p<0.001) levels, and attenuated T cell activation (227).

#### 3.5.4 Flavonoids

Lycopene, an antioxidant found in guava, grapefruit, tomatoes, watermelon, and red carrots, may augment oxidative stress and inflammation in lupus. In a six-year study, higher levels of serum lycopene were shown to be protective against mortality in SLE patients (228). In cultured human T cells, another dietary flavonoid, apigenin (40uM), was shown to cause the chronically overactivated CD4^+^ T cells to undergo apoptosis through inhibition of NF-kB activity below basal level (p<0.01) (229). Apigenin is found in common herbs and vegetables, such as parsley, thyme, peppermint, olives, and chamomile. It was shown to suppress the anti-apoptotic pathways, including NF-kB, cFLIP, and COX-2 pathways, which contribute to autoimmunity and lupus through the maintenance of chronic immune activation and lymphoproliferation (229).

#### 3.5.5 Other vitamins

Antioxidants properties of Vitamin C and E may lower chronic inflammation in SLE (230). Vitamin E levels is lower in SLE patients, preceding diagnosis (231). Vitamin C intake was associated with a lower risk of active disease (p=0.005) in a prospective study of Japanese SLE patients, possibly through the decreased production of autoantibodies and reduced oxidative stress (232). B vitamin levels in SLE patients are lower ≤180 pg/mL, in particular, B2 (riboflavin) and B12 (cobalamin) (233). They help regulate the levels of cytokines and inflammatory markers, such as homocysteine, as Vitamins B6 (pyridoxine), B12, and B9 (folate) are important factors in homocysteine metabolism (234). The combination of B12 and folic acid supplementation reduces homocysteine levels by 18% (235). Vitamin B6 and B9 also act as coenzymes in antibody and cytokine metabolism, thus, deficiencies may contribute to dysregulated immune responses and risk of vascular events (234, 236, 237). Intake of B2 and B1 (thiamin) were inversely associated with carotid atherosclerotic plaque (238), and B6 showed a decreased risk of active SLE (p=0,04), possibly through decreased homocysteine levels, in a Japanese cohort of patients (232). There was an inverse, but insignificant association for folate and no association found for B12. Vitamin A is a well-known anti-inflammatory vitamin and is involved in immune system function, with potent anti-infective effects and contributions to immune cell maturation and regulation (239–241). Vitamin A is plays a central role in balancing Th17/Treg cells, through blocking IL-6, IL-21 and IL-23 signaling in naïve T cells inhibiting their differentiation into Th17 cells, while promoting Tregs *via* TGF-β (242).Vitamin A is not a single entity, but a group of related nutrients mostly composed of retinoids and carotenes and found in animal products and plants respectively. SLE patients with low vitamin A show an altered ratio of regulatory and helper T cells; that is, a higher percentage of proinflammatory Th17 cells (26.9 ± 62.2 *vs.* 3.5 ± 2.3, p=0.000) (243), which has been correlated to higher disease activity and organ damage (244, 245). *In vitro* treatment of CD4^+^ cultured cells from these patients with retinoic acid (0.3 µg/ml) showed an improved Th17:Treg balance, with decreased Th17 (p=0.000) and increased Treg (p=0.000) (243). However, the variable responses highlight the possibility that SLE T cells may have an underlying defective response to Vitamin A or may have reduced regulatory capacity (243).

#### 3.5.6 Sodium, potassium

Studies on the effect of sodium in SLE are limited, but recent work has highlighted that low sodium regimen diet resulted in a reduction of Th17 percentages (p=0.001), while Tregs percentages increased, therefore suggesting a link between salt intake and autoimmunity (p=0.02) (246). One study exploring high dietary intake of sodium and low potassium intake in SLE patients found an association with an increased risk of high-sensitivity CRP (p=0.004), a marker of disease activity and cardiovascular risk, supporting the potential for sodium to adversely affect inflammation (247). High sodium was also associated with anti-dsDNA (p=0.001) and complement C4 (p=0.039), while low potassium was associated with C3 level (247). These findings indicated that these disease activity biomarkers in lupus may be affected by consumption of sodium and potassium. Neither sodium nor potassium was associated with clinical markers, including SLEDAI and systemic lupus damage index score (SDI); however, this cohort was at an earlier stage of disease. As the majority of patients in this study were strikingly well over the recommended maximum daily intake of sodium, and most had inadequate intakes of potassium, patients may be advised follow a diet that is composed of potassium-rich foods and limited salt.

### 3.6 Dietary effects on the microbiome

In SLE, growing evidence suggests that perturbations in the gut microbiome may influence symptoms and progression (248), through mechanisms such as bacterial translocation (249), molecular mimicry (250), and microbial metabolites (251). Some species like *Bifidobacterium* might have protective effects, inducing Tregs and promoting mucosal homeostasis (252, 253). Other species, such as *Streptococcus* and *Ruminococcus gnavus*, are expanded in SLE and in lupus nephritis and are theorized to contribute to auto-antibody formation through molecular mimicry and the initial activation of B cells and CD4^+^ T cells (254–256). In a small group of hospitalized SLE patients (257), the microbiota of SLE patients were less diverse and more heterogenous than their healthy family members attributed to their diet and symptoms. The over abundant *Lactobacillus* negatively correlated with total energy, protein, zinc, and Vitamin B2, while the less abundant *Clostridium* [a phytonutrient-sensitive species that may promote Tregs cells and mucosal thickening (258, 259)] was positively correlated with total nutrient intake and negatively with SLE disease flares. An association of *Lactobacillus* to improved SLE symptoms and autoantibody production were seen in some studies (260, 261), but another reported negative effects including outgrowth to internal organs, activation of pDC/IFN pathways, and increased mortality (259). This suggests that *Lactobacillus* may be beneficial or harmful in SLE depending on underlying host and environmental conditions. Intriguingly, the negative effects in the later study were ameliorated by increasing resistant starch, which prevented both the overabundance (ileum p<0.001) and reduced the percent of SLE mice with translocation to internal organs from approximately 90% (n=9) to 30% (n=10) (0% in wild type mice; n=9) (259). This fiber-rich diet increased types of bacteria which ferment fiber into SCFAs, which are an important group of metabolites linked to Tregs, gut integrity, anti-inflammatory pathways and disease when absent. In turn, this suppressed growth of *Lactobacillus* locally and increased the abundance of *Clostridium*. This study highlights the importance of dietary fiber in controlling outgrowth of bionts and preventing activation of immune pathways in susceptible individuals and opens the possibility for personalized dietary interventions to promote gut microbiome homeostasis and reduce systemic inflammation.

## 4 Alopecia Areata (AA)

### 4.1 Pathogenesis

Although the clinical manifestations are different and more localized in comparison to SLE and MS, immune system mediated skin diseases such as alopecia areata (AA), psoriasis, and vitiligo share a similar background of chronic inflammation. AA is a common, non-scarring type of hair loss due to autoimmune attack and destruction of the hair follicle with a loss of immune privilege (262, 263). The amount of hair loss varies across patients, with some patients exhibiting well-defined patches of scalp hair loss (most common; “Patchy alopecia”) to complete scalp hair loss (“Alopecia totalis”), as well as entire body hair loss (“Alopecia universalis”) (264, 265). Most patients experience a disease course that is sudden in onset, and then relapsing and remitting (265). The pathogenesis of AA is multifactorial, with contributions from genetic, epigenetic, immunologic, gut and skin microbiome, allergy, and oxidative stress factors. Genetic studies have shown that AA is a complex, polygenic disease (266). GWAS and meta-analysis studies have identified multiple susceptibility loci which were linked to signaling pathways in hair follicle cycling and development, as well as immune function-related genes including interferon and T cell activation and proliferation regions, among others (4, 265–267).

The collapse of the hair follicle immune privilege is central to AA pathogenesis, but what causes its breakdown is not fully understood ^258^. Important roles have been attributed to a downregulation of local immunosuppressive molecules, increased secretion of IFN-γ, TNF-α around the hair follicle by NK or activated T cells (268), in turn, IFN-γ induces the expression of MHC-1, NKG2D^+^CD8^+^ T cells, NK cells and CXCL9, CXCL10 and CXCL11chemokines (269–271) that perpetuate a cascade of inflammation as reviewed by Bertolini et al., and Rajabi et al. (262, 268). It is notable that to date, a specific autoantigen in AA has not been clearly identified, but hair follicle autoantigens are suspected (272, 273). Some studies suggest that the initial triggers for antigen presentation are induced by a stressed hair follicle environment, while other studies indicate an infiltration by a dysregulated immune system as the inducing factor (271, 274).

### 4.2 Influence of environmental factors during the lifespan

AA can occur at any age and the prevalence of AA is slightly higher in children than in adults (275, 276), although mean age of onset has been between 25.3 and 36.3 years (277). Many adult and pediatric AA patients with limited hair loss spontaneously recover, but it often follows a relapsing and remitting course; in more severe forms it can progress to complete hair loss (278). A more chronic and relapsing course generally occurs in patients with a childhood onset, more severe presentation and a family history. AA onset is associated with triggers such as infections, trauma, hormones, and stress (279), leading to increased IFN- α/IFN-γ, CXCL10, IL-2, IL-13, IL-17 (280) that might initiate the immune privilege breakdown of the hair follicle at any point over the lifespan (281). Environmental factors may cause local disturbances in the hair follicle, through the buildup of ROS within keratinocytes (282, 283). This oxidative stress may contribute to the loss of immune privilege and autoimmune attack through an upregulation of activating ligands in the stressed follicle (i.e., NKG2D) (269). Indeed, oxidative stress has been linked to AA (284, 285) as patients’ blood and scalp samples show higher levels of serum nitric oxide, and total oxidant capacity as well as lower levels of superoxide dismutase (p<0.001), glutathione peroxidase (p<0.001), and total antioxidant capacity. The level of imbalance was also correlated with disease severity, with higher levels of oxidative stress in more severe cases (95% CI 1.43-0.71) (284, 286, 287). Furthermore, GWAS studies looking at the underling genes involved in AA have found a link to the antioxidant enzyme PRDX5, which has also been associated with MS (4). Therefore, an cumulative exposure to environmental factors throughout the years which cause oxidative stress to the skin, like UV light and chemical pollutants, may lead to excess ROS production and a background of chronic inflammation, which ultimately may contribute to the development of AA in predisposed individuals. Mercury, a toxin which increases oxidative stress in exposed individuals, has been thought to cause AA through overconsumption of high-mercury fish in a case study and was reversible with an altered diet (288). Others have also linked AA cases to toxic metals, including thallium, mercury, non-metal selenium, and arsenic, and postulate that these substances may induce AA pathology through imbalanced zinc homeostasis and blocked cross-linking in keratin (289).

Interestingly, AA is commonly comorbid with other autoimmune conditions, including lupus (290) and inflammatory bowel disease (291), implying a potential common link between gut health and chronic inflammation in these disease states. Like in other autoimmune conditions, a dysbiosis in the gut microbiome has been suggested in AA (292), with enrichment in certain species that may be useful as biomarkers. A case report demonstrated regrowth in two patients after undergoing a fecal microbiota transplant (293). However, a clear link has not been established as there is very limited research. Other potential environmental factors in the development of AA are discussed in detail in a recent review, and include allergy, skin microbiota, and epigenetic changes, such as DNA methylation (265).

As these authors note, a promising feature to treating AA is the fact that the hair follicle’s epithelial stem cells typically withstand the autoimmune attack and therefore in most cases AA can be reversed (279). Decreasing the background inflammation that deregulates the hair follicle growth pattern and restoring the immune privilege of the hair follicle to prevent future attack are important strategies that may lead to remission for patients (268, 294).

### 4.3 Vitamin D

The role of Vitamin D and its receptor (VDR) in the hair cycle have been widely explored but remains poorly understood (295). Without proper VDR function hair follicles are born but are unable to maintain themselves and loss occurs, as shown by *in vitro* and mice studies prompted by observations from patients with a rare genetic disorder called type II vitamin D-dependent rickets, whose hair is normal at birth and then lost completely (296, 297).

However, while lower VDR amounts (p=0.000) are noted in AA patients’ serum (9.99 ± 1.69 ng/mL) and scalp tissue (199.7 ± 33.38ng/mL) (14, 298), genetic VDR polymorphisms that may contribute to risk of developing AA have not been shown, albeit in a limited sample of patients (14, 299). Nonetheless, low levels of serum Vitamin D (11.84 ± 6.18 ng/mL) have been reported by several groups in AA patients compared to controls p<0.001and are thought to contribute to pathogenesis, as lower levels are correlated with increased disease severity (13, 300, 301).

### 4.4 Obesity

Like other autoimmune conditions, AA has been associated with obesity and a higher BMI odds (ratio, 1.15; 95% confidence interval, 1.02–1.29; *p* = 0.0207) were reported in AA cases (26), likely due to the pro-inflammatory effects of obesity. In addition, the association may also be related to a decrease in immune modulating adipokines, such as adiponectin, which has been shown to be lower in AA patients and correlates with disease severity (28). More recently, high fat diet was shown to increase hair thinning by depleting hair follicle stem cells (HFSCs) through epidermal keratinization that activates the NF-kB pathway and/or autocrine and paracrine IL-1R to generate an excess of reactive oxygen (302).

### 4.5 Diet

Many patients with these skin conditions attribute a significant role of diet in their disease management, both in terms of inflammatory triggers and anti-inflammatory mechanisms. In several cases of AA patients with comorbid Coeliac’s disease, a gluten-free diet resulted in hair growth (303, 304). Other possible triggers shown in different subtypes of alopecia include caloric deprivation, buckwheat, and millet groats, however, these have not been explored in AA per se to our knowledge (40).

A restricted clinical study on dietary protein deficiency demonstrated that all AA patients with an adequate protein intake (>30g/day) and early morning breakfast habits prevent deregulation of autophagy, which may in turn be protective for autoimmune conditions including hair loss disorders (305). The authors postulated that protein adequacy and early breakfast may play a role in preventing follicle destruction or deregulated autophagy, as the body may redirect the limited protein intake to more critical organs and/or subsequently these non-renewed senescent collagens may release antigens that contribute to autoimmunity in predisposed individuals. However, AA patients were also found to have significantly lower folate (Vitamin B9) (p<0.001) (37) and Vitamin D (300), and hypothyroidism (306) and thus conclusions regarding a single factor cannot be made (307).

On the other hand, diets associated with improvement include the Mediterranean diet, rich in vegetables, herbs, and fruits that contain high amounts of anti-inflammatory and antioxidant substances as well as sufficient protein, necessary for hair health (40). A reduced risk for developing AA is associated with soya-based eastern diets compared to western diets (308, 309). The protective effect may be mediated by soy isoflavones; phytoestrogens which have estrogen-mimicking, antioxidant effects, and stimulates hair growth through increased insulin growth factor-1 (310, 311). A diverse diet may be needed to obtain adequate amounts of the vitamins and minerals necessary to support the high metabolism of the rapidly dividing hair follicle cells. These micronutrients are also important in lowering oxidative stress, which is implicated in the pathology of AA (312).

#### 4.5.1 Vitamins in AA

Micronutrients have been explored in AA as modifiable risk factors for disease development and progression, including vitamins and minerals, but few studies have provided sufficient evidence to make recommendations from and more research is warranted. Kantor et al. (313), proposed a “threshold hypothesis”, in which patients with mild hereditary susceptibility might have a threshold micronutrient level under which disease could develop while patients with high hereditary susceptibility may develop AA regardless of micronutrient status. Sub-optimal micronutrients in the mild predisposition group may lead to disease through aberrant immune cell function, DNA synthesis, and oxidative stress. Deficiencies in biotin (B7) (<100ng/L) and folate (B9) (110.62-243.75 ng/mL/cells) are associated with hair loss and AA (36, 37). To date, it appears that vitamin B application has not been tested in AA. The sparse, contradictory studies preclude a strong conclusion regarding B vitamins in general (307), however, a study suggested low red blood cell folate may play a role in the risk for AA and progression (37). Interestingly, AA patients have been shown to have higher levels of genetic polymorphism in the enzyme methylenetetrahydrofolate reductase (MTHFR), a regulator of folate metabolism and homocysteine levels (314). Polymorphisms in this gene and increased homocysteine levels have also been associated with MS (315) and SLE (316), suggesting a common link.

Other micronutrients, such as vitamin D (301), zinc (317), and folate (37) have been found to be lower in patients with AA, while the evidence is currently insufficient in terms of iron, vitamin B12, copper, magnesium, and selenium. Iron deficiency can cause diffuse hair loss (318) and is therefore currently recommended to screen for and treat in AA management. The primary measure of iron status used is ferritin, which has been shown to be lower in 56% of AA patients(<40ng/mL) (38), but other studies have not found an association (319) and has been reviewed by Tompson and colleagues (307). Many studies used only female participants which may be a factor (319–321). Vitamin A is important in hair follicle cycle and in immune privilege, as a regulator of antigen-presenting (APC) cells (322). APCs express STAR6 which binds to retinol binding protein, inducing retinaldehyde dehydrogenase (RALDH) 2 that metabolizes retinol to atRA, and upregulates the transcription of MMP-9 and CD1, inducing iTregs and stimulates IgA isotype switching by B cells (322). Retinoic acid has been shown to increase T-cell proliferation (323), antigen-presenting capacity of dendritic cells (324), while decreasing B-cell proliferation (325). Lower levels of beta-carotene, a precursor to Vitamin A, were found in some AA patients (326). Vitamin A toxicity can also cause alopecia (327). A study of AA patients and model animals identified a dysregulation in retinoid synthesis genes, with an increase in synthesis and decrease in breakdown, which may make patients more sensitive to exogenous Vitamin A (328).

### 4.6 Dietary effects on the microbiome

To date, few studies have explored the gut microbiome in AA, but preliminary evidence of gut dysbiosis exists (292),including in pediatric patients as compared to their siblings (329). The authors in the pediatric study noted that the transition from the early childhood microbiota to a more adult-like microbiota occurs near the timing of the initial presentation of AA and may be influenced by diet. Notably, the predominant diet in this study was Westernized. In a recent review, the potential for our modern diet and its impact on gut integrity is thought to be driving factors towards AA development susceptible individuals (330). Although this is a single paper, it is promising and in line with other autoimmune research exploring the recent rise of autoimmune disease alongside the rapid growth of industrial food processing and additives (331). One mechanism frequently proposed is an altered equilibrium between the gut and the immune system, through dysfunctional “leaky” intercellular tight junctions in the intestinal epithelial barrier. Common additives used in food which can affect intestinal permeability include glucose, salt, emulsifiers, organic solvents, gluten, microbial transglutaminase, and nanoparticles. Once the intestinal integrity is breached, immunogenic antigens on these molecules may activate the autoimmune cascade. Exposure to organic solvents has been associated with MS and other autoimmune diseases (332), and individuals with risk factors are advised to avoid consuming these. Ultra-processed food has low nutritional value, low fiber and protein, with added salt, sugars, oils, as well as additives like coloring, flavoring, stabilizers, and preservatives which extend its shelf life. Chemical additives including phthalates and bisphenols are known to disrupt the endocrine system, and higher dietary consumption is associated with high levels of urinary phthalates (333). In addition to a shortened lifespan, ultra-processed food consumption has been associated with a plethora of deleterious health effects, including cancer, chronic disease, and obesity (255, 256).

## 5 Genetic susceptibility to environmental factors

Over the past years, growing evidence suggests that genetic architecture and susceptibility to environmental factors increases the odds to develop autoimmunity. The international MS Genetics Consortium identified 233 genome variations as genetic risk factors on the x chromosome, and overall influencing different immune-cell types and tissues (2), while in SLE over 60 locis are reported to be associated with innate and adaptive immune function dysregulations (3, 334, 335). In AA, 139 SNPs were identified as genetic risk factors, mainly controlling Treg functions and HLA (4). The autoimmune conditions discussed in this review are complex diseases due to their multifactorial nature, including the ambiguous interplay between genes and environmental factors that may trigger and exacerbate disease. It is hence challenging to point to a single genetic or environmental factor, and in addition, the differential impact of environmental factors on immune function may be explained by the timing of exposures during development. It is becoming clearer that in MS and SLE the preclinical phase starts prior the appearance of clinical manifestations, given the accelerated brain atrophy of patients with MS after the first clinical presentation (336). While in SLE, early longitudinal studies suggest the detection of auto-antibodies in healthy individuals transitioning to SLE as a potential biomarker, although more studies are needed (337, 338). In an effort to identify effective means to prevent onset of disease or delay progression, a great deal of effort is focusing on the identification of genetic, immune and environmental profiles that may enhance the risk of an individual to develop autoimmune conditions. To efficiently accomplish this goal, the ideal cohort represents first degree family members of patients with MS, SLE or AA considering the genetic pre-disposition they have at least 30 times greater chances than sporadic cases to develop the disease (339–341). Our group deployed a large cohort of first-degree family members of patients with MS (the genes and environment in MS (GEMS) cohort, n=2,632 participants) (342, 343) to establish a tool that successfully predicts an MS risk score based on a mathematical model that accounts for the most robust environmental and genetic factors known so far (sex, BMI, smoking status, mononucleosis infection status, and HLA SNP allele) (342, 344). Overall, these studies provided foundations to leverage existing findings and develop a personalized tool to calculate MS risk scores and help identify high-risk individuals (such as family members) prior autoimmune disease onset. Such tools are crucial to develop improved preventive care strategies and to shed light on autoimmune dysfunctions. The initial prospective studies of high-risk siblings were completed in type 1 diabetes (DAISY) (345). Similar studies have been conducted in potential at-risk SLE populations, including family members (346) and women in the prospective Nurses’ Health Studies (347). In at-risk family members, researchers found preclinical alterations in levels of inflammatory mediators that may predict transition (348), as well as a greater SLE risk when genetic susceptibility is combined with vitamin D status and smoking (9, 349), suggesting that environment may influence specific pathogenic SLE genes and is a useful component in estimating risk. Future efforts to develop predictive tools using gene-environment interactions in SLE and in AA could be inspired by those developed in the DAISY and GEMS cohorts.

## 6 Perspectives: therapeutic interventions

In addition to the established treatments and DMTs to treat MS, SLE and AA, diets and supplements known to confer anti-inflammatory benefits are of increasing interest as a potential way to reduce chronic inflammation. Patients often seek medical guidance to adopt a suitable lifestyle to cope with relapses, flares, or control disease progression. However, inadequate guidance is provided in this area despite emergent bodies of research demonstrating the benefits of complementary therapeutic options, such as vitamin supplementation, diet and overall, a healthier lifestyle. The latter suggests that further investigations to assess the precise impact of complementary therapeutic interventions along with approved DMTs should be explored to understand their impact on the immune system and on the disease course.

### 6.1 Add-on therapies: vitamin D

As discussed above, vitamin D is a common risk factor for MS, SLE and AA associated with greater disease activity and duration. Hence a number of clinical trials attempted to use vitamin D as a therapy, although conflicting results emerged.

A new, large-scale, double-blind randomized controlled trial from Brigham and Women’s Hospital found that participants who took vitamin D, or vitamin D and omega-3 fatty acids, had a significantly lower rate of autoimmune disease including MS, SLE, and AA (350). The strongest effects were found after two years of supplementation and for participants with a lower body mass. This study suggests that vitamin D could be used as a primary preventative measure to lower incidence of autoimmunity in older adults. Future research could extend these findings to young adults and high-risk family members. These findings along with the associations between low vitamin D and disease activity in autoimmunity suggests that the use of vitamin D as an add-on therapy or as supplements in patients with MS, SLE or AA could be beneficial to control autoimmune disease activity and progression.

Add-on Vitamin D therapy for MS in addition to IFN-β therapy was tested in the context of phase 3 clinical trials, and suggested a beneficial additive effect on disease activity (350, 351). In contrast, a meta-analysis based on several clinical trials using vitamin D supplementation as an MS add-on therapy suggests there is no strong therapeutic effect on disability, nor on relapse rate (352). In lupus, cross-sectional studies exploring the impact of supplementation on fatigue and clinical disease activity measures have failed to show a benefit of supplementation, while prospective studies and RCTs have shown a benefit, possibly through a suppression of interferon signature gene expression which is elevated in SLE patients and correlated to disease activity (353–358). A meta-analysis of available RCTs showed a significant improvement in SLEDAI scores, fatigue (when assessed in two studies), and serum C3; in contrast, serum C4 and anti-dsDNA changes were insignificant (359). In AA, application of topical calcipotriol, a vitamin D analogue, has been shown to be an effective treatment in most mild-to-moderate patients (298, 360, 361), particularly those with deficiencies (362). However, these studies in AA often lack placebo arms and randomized, clinical trials with larger groups of patients are greatly needed.

Moreover, PUFAs are protective in MS, perhaps through their anti-inflammatory properties clinical trials aimed to test the efficacy of PUFAs supplementation in patients with MS. Although they obtained mixed results (363) suggesting PUFAs should be tested in combination with other add-on therapies like Vitamin D, as shown to be efficient to reduce autoimmunity rates (350).

Overall, the plethora of research papers and conflicting results citied in this review point to the need for robust randomized controlled trials to determine the dosage of vitamin D supplementation that will offer long-term benefits for MS, SLE, and AA patients. Future studies should enroll large numbers of patients from diverse demographics, should be cross-sectional, and control for the dose of vitamin D administered. Although challenging, vitamin D supplementation should be compared in conjunction with DMTs, other vitamins, and presence or avoidance of ultraviolet radiation which is crucial for the conversion of vitamin D. Finally, future therapeutic strategies should aim to move towards personalized therapeutic strategies considering the genetic background of each enrolled patient. Emerging studies may help resolve the ambiguity between vitamin D deficiency, and autoimmune risk/progression.

### 6.2 Other add-on therapies

Other vitamins have been explored in autoimmune disease for their potential anti-inflammatory and immunomodulatory qualities. Vitamin E decreases autoantibodies in SLE (364) and when combined with *Nigella sativa* (aka black cumin, antioxidant), patients had improved SLEDAI score, inflammatory markers, and increased GSH and superoxide dismutase antioxidant levels (365). In MS, vitamin E improved oxidation markers and telomere length maintenance (366). In an RCT in SLE patients, combined Vitamin C and E administration decreased lipid peroxidation but did not affect other markers of oxidative stress or endothelial function involved in CAD risk. However, at baseline these patients were not deficient and studies with a longer duration of treatment or higher doses could be explored (230). No studies exploring isolated Vitamin C in MS were found, nor vitamin C or E in AA. In SLE patients, Vitamin A treatment increased antibody-dependent cellular cytotoxicity (ADCC) functions in effector to target cells (p<0.001), NK cell cytotoxicity measured using Cr-labeled K-562 target cells (p<0.001), and IL-2 activities measured in PBMC treated with concanavalin A (p<0.001), while decreasing anti-dsDNA and proteinuria in lupus nephritis (367, 368). *In vitro* treatment of CD4^+^ T cells with vitamin A (0.03 µg/ml) modulated the Th17/Treg balance towards Treg (p=0.000) (243). In MS RCTs, vitamin A decreased IL-17, IFN-γ, retinoic acid–related orphan receptor γτ and T-bet expression, fatigue and depression (369, 370). In AA, topical retinoids have shown regrowth in some patients. A randomized, ‘half-head’ trial using Bexarotene, a selective retinoid which induces T-cell apoptosis, showed regrowth in some patients (371). However, these studies could not differentiate this from spontaneous regrowth or growth in Vitamin A deficient patients, and the aforementioned duality remains in which too much vitamin A can cause alopecia (327).

Case reports in MS have showed an improvement in fatigue with high-dose thiamine (B1) supplementation (372). Biotin (B7) improved visual acuity, muscle strength, VEPs, fatigue, coordination, and mood symptoms in a pilot study, and an RCT showed modest motor score improvement in MS; however, follow-up RCTs have not shown an effect on disability or walking speed (373). Biotin in AA has been explored as a combination with zinc and topical clobetasol, which did help regrowth in some patients, but no conclusions can be made about it as a single agent (374). In MS, zinc sulfate supplementation significantly improved depression in compared to placebo (375). The few conflicting studies on oral zinc in AA highlight the possibility of subgroups of responsive and unresponsive patients (376–378). Zinc has not been explored in lupus, likely due to animal studies suggesting a potential negative effect (379).

#### 6.2.1 N-acetyl cysteine, curcumin, and Royal Jelly

Ameliorating oxidative stress by raising levels of depleted GSH is an interesting area of research for therapeutic intervention. Direct administration of GSH has failed due to bioavailability constraints (380), therefore, repletion of GSH with its precursor and antioxidant, N-acetylcysteine (NAC) is a potential way to overcome this challenge. In SLE, NAC safely and significantly blocked the mTOR activation underlying dysfunction in T cells, reduced anti-DNA production, and improved disease activity in a double-blinded RCT (381). Moreover, it raised levels of CD4^+^CD25^+^FoxP3^+^ Treg population, deficient in active SLE (381–383). NAC reduced kynurenine levels, a tryptophan metabolite that is abnormally elevated in and specific to the SLE metabolic profile that may contribute to mTOR activation (384). In two cases of early lupus nephritis, NAC in addition to standard therapy improved GSH levels, lipid peroxidation, blood counts, 24-h urine protein, erythrocyte sedimentation rate, and SLEDAI (385). In MS, a clinical trial evaluating the potential neuroprotective effects of NAC is ongoing (386). A previous study found an increase in cerebral blood flow and qualitative improvements in cognition and attention with NAC alongside standard of care in patients (387), as it is thought that decreasing oxidative injury may lessen brain degeneration (388). To our knowledge, NAC has not been explored in AA. However, one study found topical crude onion juice significantly regrew hair compared to tap water (389) which is intriguing onions contain GSH and may stimulate GSH production systemically and epidermally (390). Finally, diet may also be a means by which to simulate GSH synthesis as increased intake of cruciferous vegetables improves GSH levels and reduces oxidative stress (391–393). Therefore, quality, and quantity could affect sensitivity to oxidative stress within each day and should be considered when designing future studies.

Curcumin is an anti-inflammatory molecule that positively impacts autoimmune patients, a polyphenol contained in turmeric spice. In SLE, curcumin inhibited cell proliferation, modulated Th17/Treg balance, and reduced proinflammatory cytokines (394, 395). A RCT demonstrated decreased proteinuria, hematuria, and systolic blood pressure in relapsing or refractory SLE nephritis with no adverse effects (396). In MS, a curcumin nano formulation improved EDSS score, reduced inflammatory mediators, miRNAs, IFN-γ, CCL2, and CCL5, and increased Sox2, Sirtuin-1, Foxp3, PDCD1 (397). Other studies found decreased Th17 frequency, and IL-17 and RORγt alterations (398), as well as restored regulatory T cell frequency and function (399). In AA, curcumin has not been tested individually, but was effective in a mixed preparation with piperine and capsaicin, albeit not superiorly to minoxidil (400). Finally, Royal Jelly (RJ) is a milky secretion of water, proteins, carbohydrates, fatty acids, and other compounds, with pleotropic functions including control of honeybee development epigenetically by DNA methylation (401). RJ has been explored for its antioxidant, anti-inflammatory and immunomodulatory properties. A 2016 open-label study on pediatric SLE found RJ improved SLEDAI score, increased C3 and C4 levels, and increased regulatory CD4^+^ and CD8^+^ T cells (402). However, further research in larger cohorts is needed, as well as trials in AA.

#### 6.2.2 Diet

Few clinical trials have explored whole-diet interventions in autoimmune disease, despite evidence that broad dietary changes can be synergistic and may be more effective than isolated nutrient or food administration as reviewed in (403, 404). A pilot RCT in MS found a modified Paleolithic diet improved fatigue, quality of life, exercise capacity, hand/leg function, and vitamin K levels (405). A balanced diet with high fiber intake, polyunsaturated fatty acids, polyphenols, vitamins, minerals, antioxidants, with a relatively lower but adequate consumption of calories, proteins, and carbohydrates throughout the lifespan, may enable prevention of disease and is overall beneficial towards a healthier lifestyle, improving autoimmune disease progression. According to the work discussed in this review, the Mediterranean diet may have the strongest potential to improve immune system function in health and disease, with its high proportion of antioxidant and anti-inflammatory components that operate directly and indirectly through the microbiome to support efficient immune functions. A clinical trial comparing the Mediterranean and high-fermented food diet in SLE is ongoing (406).

In AA, clinical trials using dietary intervention are needed. One pediatric AA case study highlights the potential benefit, as complete remission was achieved over 5 months with a diet of unrefined foods, rich in vitamins A and D, and zinc as well as supplements like zinc sulfate, fish oil, and vitamin D (407). This warrants further investigation in clinical trials, including in adults as the timing of intervention may be of importance.

A potential mode of action of diet includes its impact on the epigenome, as it has been shown that poor dietary habits may precipitate epigenetic changes leading to chronic inflammatory disorders over time; hence changes to daily diet may help to prevent or reverse these epigenetic aberrations (408). Recent work has demonstrated that DNA hypomethylation in SLE cells is a dominant pattern and driving force towards autoimmunity and severity (409). Altered methylation patterns have also been identified in AA (410, 411) as well as MS (412, 413), showing an even more prominent alternation on PPMS than RRMS which may contribute to the distinct progression patterns. The mechanisms causing aberrant DNA methylation patterns in SLE, AA, and MS are poorly understood, however, molecules important to maintaining DNA methylation patterns, including S-adenosylmethionine (SAM), require dietary nutrients like methionine, choline, and B vitamins to function properly. In SLE, it has been suggested that low nutrient levels might precipitate T cell epigenetic changes caused by oxidative stress after CD4^+^ T cells cultured in low methionine showed a greater overexpression of methylation sensitive genes (414). Furthermore, patients with active SLE have been shown to have deficiencies in transmethylation nutrients; therefore, a methyl-donor poor diet may worsen disease activity through dysregulated methylation patterns (414, 415). In MS, reduced methionine levels and dysregulated B12-dependent methionine metabolism peripherally and centrally has been observed and is associated with altered methylation patterns as well as neural mitochondrial abnormalities (416, 417). This was consistent with a prior report showing lower levels of methionine, SAM, and vitamin B12 in MS patients at different disease stages (418).

#### 6.2.3 Dietary effects on the immunomodulatory gut microbiome

Gut dysbiosis is increasingly implicated in autoimmune disease, including MS (123, 125, 126), SLE (212, 248, 254, 256, 257), and AA (265, 292). Fecal transplants have been shown to ameliorate symptoms of MS (122) and SLE and resolve AA (293) in several cases. However, changes to diet may be a more feasible long-term approach in both treatment and prevention, as dietary patterns have been shown to profoundly and rapidly affect the human gut microbiome (419). Overall, a diet that is rich in fiber will have a beneficial effect on immune homeostasis through the gut microbiome. Fiber is metabolized by colonic bacteria and increases the growth and diversity of gut bacteria. Consumption of fiber is low in westernized diets and stimulates pathogenetic bacterial growth which may provide a stimulus for autoimmunity (208, 420, 421).

## 7 Limitations and future directions

Autoimmunity is thought to emerge from the complex interplay between a multitude of environmental and genetic risk factors, which renders the chase for causation factors difficult. Studies discussed in this review thus far are mostly restricted to a retrospective approach, therefore one cannot determine if these alterations in diet, microbiome, or past infections are contributing to autoimmune development or as a result of disease per se. Hence, to further our knowledge about the interaction between environmental factors and genes in the context of autoimmunity, future studies should aim to deploy larger cohorts and with a prospective design. In addition, the choice of cohort is crucial to collect reliable data. For instance, if the goal is to investigate the influence of a given environmental factor prior disease onset, it is imperative to deploy a large cohort of first-degree family members of autoimmune patients. An excellent example of a successful retrospective study design represents the association between EBV infection with MS disease onset using a massive longitudinal cohort (10 million participants) (420). Whereas, if the goal is to assess the impact of add-on therapies on disease progression, the desired cohort would be patients diagnosed with early-stage MS, SLE or AA. Other factors that may influence cohort inclusion criteria should account for the ethnic background and the age of participants, given the growing evidence of the impact of interindividual immune variations on the immune system; such variables should also be corrected for during data analysis stage (422). Unfortunately, many publications use inconsistent inclusion criteria for assessing the effectiveness of the same nutrition or supplementation on patients to evaluate their impact on the same disease. All three autoimmune conditions discussed here can follow a waxing and waning course, therefore the stage at the time of intervention may alter the effectiveness, especially on clinical measures. Overall, studies cited in this review conducting meta-analysis based on observational epidemiologic studies must account for systematic error biases while evaluating the data, as it may distort findings interpretation. As a result, interpretation can include reverse causation: the disease itself causes the association; or omitting confounding factors during data analysis such as: stratification of cohorts based on disease stage, forms of disease and self-reported ethnicities *versus.* genetic ancestries.

## Author contributions

HT designed the review. HT and KM wrote the manuscript and designed figures. All authors contributed to the review and editing of the final manuscript. All authors contributed to the article and approved the submitted version.

## References

[1] Faissner S Gold R. Efficacy and safety of multiple sclerosis drugs approved since 2018 and future developments. CNS Drugs (2022) 36(8):803–17. 10.1007/s40263-022-00939-9 PMC9307218 35869335

[2] International Multiple Sclerosis Genetics, C. Multiple sclerosis genomic map implicates peripheral immune cells and microglia in susceptibility. Science (2019) 365(6460). doi: 10.1126/science.aav7188 PMC7241648 31604244

[3] Bentham J Morris DL Graham DSC Pinder CL Tombleson P Behrens TW. Genetic association analyses implicate aberrant regulation of innate and adaptive immunity genes in the pathogenesis of systemic lupus erythematosus. Nat Genet (2015) 47(12):1457–64. 10.1038/ng.3434 PMC4668589 26502338

[4] Petukhova L Duvic M Hordinsky M Norris D Price V Shimomura Y. Genome-wide association study in alopecia areata implicates both innate and adaptive immunity. Nature (2010) 466(7302):113–7. 10.1038/nature09114 PMC2921172 20596022

[5] Soldan SS Lieberman PM. Epstein-Barr Virus and multiple sclerosis. Nat Rev Microbiol (2023) 21(1):51–64. 35931816 10.1038/s41579-022-00770-5 PMC9362539

[6] Holick MF. The vitamin d deficiency pandemic: approaches for diagnosis, treatment and prevention. Rev Endocr Metab Disord (2017) 18(2):153–65. 10.1007/s11154-017-9424-1 28516265

[7] Orton SM Wald L Confavreux C Vukusic S Krohn JP Ramagopalan SV. Association of UV radiation with multiple sclerosis prevalence and sex ratio in France. Neurology (2011) 76(5):425–31. 10.1212/WNL.0b013e31820a0a9f PMC3034408 21282589

[8] Cancela Diez B Pérez-Ramírez C Maldonado-Montoro MDM Carrasco-Campos MI Sánchez Martín A Lancheros LEP. Association between polymorphisms in the vitamin d receptor and susceptibility to multiple sclerosis. Pharmacogenet Genomics (2021) 31(2):40–7. 10.1097/FPC.0000000000000420 33044390

[9] Young KA Munroe ME Guthridge JM Kamen DL Niewold TB Gilkeson GS. Combined role of vitamin d status and CYP24A1 in the transition to systemic lupus erythematosus. Ann Rheum Dis (2017) 76(1):153–8. 10.1136/annrheumdis-2016-209157 PMC5360632 27283331

[10] Kamen DL Attar SM Siddiqui AM. Vitamin d deficiency in systemic lupus erythematosus. Autoimmun Rev (2006) 5(2):114–7. 10.1016/j.autrev.2005.05.009 16431339

[11] Kim HA Sung J-M Jeon J-Y Yoon J-M Suh C-H. Vitamin d may not be a good marker of disease activity in Korean patients with systemic lupus erythematosus. Rheumatol Int (2011) 31(9):1189–94. 10.1007/s00296-010-1442-1 20352222

[12] Bilgin M Keskin A Aci R Baklacioglu HS Erdem MA. Darkness hormone or daylight hormone in women with systemic lupus erythematosus? Clin Rheumatol (2023) 42(1):93–9. 10.1007/s10067-022-06379-6 36125575

[13] Tamer F Yuksel ME Karabag Y. Serum ferritin and vitamin d levels should be evaluated in patients with diffuse hair loss prior to treatment. Postepy Dermatol Alergol (2020) 37(3):407–11. 10.5114/ada.2020.96251 PMC7394174 32792884

[14] Fawzi MM Mahmoud SB Ahmed SF Shaker OG. Assessment of vitamin d receptors in alopecia areata and androgenetic alopecia. J Cosmet Dermatol (2016) 15(4):318–23. 10.1111/jocd.12224 27151518

[15] Hedstrom AK Olsson T Alfredsson L. High body mass index before age 20 is associated with increased risk for multiple sclerosis in both men and women. Mult Scler (2012) 18(9):1334–6. 10.1177/1352458512436596 22328681

[16] Munger KL Bentzen J Laursen B Stenager E Koch-Henriksen N Sørensen TIA. Childhood body mass index and multiple sclerosis risk: a long-term cohort study. Mult Scler (2013) 19(10):1323–9. 10.1177/1352458513483889 PMC4418015 23549432

[17] Mokry LE Timpson NJ Sawcer S Smith GD Richards J. Obesity and multiple sclerosis: a mendelian randomization study. PloS Med (2016) 13(6):e1002053. 27351487 10.1371/journal.pmed.1002053 PMC4924848

[18] Ahmed M Gaffen SL. IL-17 in obesity and adipogenesis. Cytokine Growth Factor Rev (2010) 21(6):449–53. 10.1016/j.cytogfr.2010.10.005 PMC3259710 21084215

[19] Asghar A Sheikh N. Role of immune cells in obesity induced low grade inflammation and insulin resistance. Cell Immunol (2017) 315:18–26. 28285710 10.1016/j.cellimm.2017.03.001

[20] Stampanoni Bassi M Iezzi E Buttari F Gilio L Simonelli I Carbone F. Obesity worsens central inflammation and disability in multiple sclerosis. Mult Scler (2020) 26(10):1237–46. 10.1177/1352458519853473 31161863

[21] Hossein-Nezhad A Mirzaei K Keshavarz SA Ansar H Saboori S Tootee A. Evidences of dual role of vitamin d through cellular energy homeostasis and inflammation pathway in risk of cancer in obese subjects. Minerva Med (2013) 104(3):295–307. 23748283

[22] Tedeschi SK Barbhaiya M Malspeis S Lu B Sparks JA Karlson EW. Obesity and the risk of systemic lupus erythematosus among women in the nurses' health studies. Semin Arthritis Rheum (2017) 47(3):376–83. 10.1016/j.semarthrit.2017.05.011 PMC5675759 28688713

[23] Cozier YC Barbhaiya M Castro-Webb N Conte C Tedeschi S Leatherwood C. A prospective study of obesity and risk of systemic lupus erythematosus (SLE) among black women. Semin Arthritis Rheum (2019) 48(6):1030–4. 10.1016/j.semarthrit.2018.10.004 PMC6459733 30424973

[24] Oeser A Chung CP Asanuma Y Avalos I Stein C. Obesity is an independent contributor to functional capacity and inflammation in systemic lupus erythematosus. Arthritis Rheum (2005) 52(11):3651–9. 10.1002/art.21400 16258902

[25] Baker JF Morales M Qatanani M Cucchiara A Nackos E Lazar MA. Resistin levels in lupus and associations with disease-specific measures, insulin resistance, and coronary calcification. J Rheumatol (2011) 38(11):2369–75. 10.3899/jrheum.110237 PMC5702914 21885493

[26] Hagino T Okazaki S Serizawa N Suzuki K Kaga M Otsuka Y. Dietary habits in Japanese patients with alopecia areata. Clin Cosmet Investig Dermatol (2021) 14:1579–91. 10.2147/CCID.S335440 PMC8560057 34737597

[27] Yang CC Hsieh F-N Lin L-Y Hsu C-K Sheu H-M Chen W. Higher body mass index is associated with greater severity of alopecia in men with male-pattern androgenetic alopecia in Taiwan: a cross-sectional study. J Am Acad Dermatol (2014) 70(2):297–302.e1. 24184140 10.1016/j.jaad.2013.09.036

[28] Stochmal A Waśkiel-Burnat A Chrostowska S Zaremba M Rakowska A Czuwara J. Adiponectin as a novel biomarker of disease severity in alopecia areata. Sci Rep (2021) 11(1):13809. 34226603 10.1038/s41598-021-92853-1 PMC8257783

[29] Armon-Omer A Waldman C Simaan N Neuman H Tamir S Shahien R. New insights on the nutrition status and antioxidant capacity in multiple sclerosis patients. Nutrients (2019) 11(2). 10.3390/nu11020427 PMC6413226 30781687

[30] Yosef N Shalek AK Gaublomme JT Jin H Lee Y Awasthi A. Dynamic regulatory network controlling TH17 cell differentiation. Nature (2013) 496(7446):461–8. 10.1038/nature11981 PMC3637864 23467089

[31] Ertas Ozturk Y Helvaci EM Kaya PS Terzi M. Is Mediterranean diet associated with multiple sclerosis related symptoms and fatigue severity? Nutr Neurosci (2023) 26(3):228–34. 10.1080/1028415X.2022.2034241 35143375

[32] Pocovi-Gerardino G Correa-Rodríguez M Callejas-Rubio J Ríos-Fernández R Ortego-Centeno N Rueda-Medina B. Dietary intake and nutritional status in patients with systemic lupus erythematosus. Endocrinol Diabetes Nutr (Engl Ed) (2018) 65(9):533–9. doi: 10.1016/j.endinu.2018.05.009 29997049

[33] Levy-Costa RB Sichieri R dos Santos Pontes N Monteiro CA. [Household food availability in Brazil: distribution and trends (1974-2003)]. Rev Saude Publica (2005) 39(4):530–40. 10.1590/s0034-89102005000400003 16113900

[34] Minami Y Hirabayashi Y Nagata C Ishii T Harigae H Sasaki T. Intakes of vitamin B6 and dietary fiber and clinical course of systemic lupus erythematosus: a prospective study of Japanese female patients. J Epidemiol (2011) 21(4):246–54. 10.2188/jea.JE20100157 PMC3899416 21515941

[35] Oh J Oda K Brash M Beeson WL Sabaté J Fraser GE. The association between dietary patterns and a doctor diagnosis of systemic lupus erythematosus: the adventist health study-2. Lupus (2022) 31(11):1373–8. 10.1177/09612033221112522 PMC9547829 35786051

[36] Trueb RM. Serum biotin levels in women complaining of hair loss. Int J Trichology (2016) 8(2):73–7. 10.4103/0974-7753.188040 PMC4989391 27601860

[37] Yousefi M Namazi MR Rahimi H Younespour S Ehsani AH Shakoei S. Evaluation of serum homocysteine, high-sensitivity CRP, and RBC folate in patients with alopecia areata. Indian J Dermatol (2014) 59(6):630. doi: 10.4103/0019-5154.143567 PMC4248520 25484412

[38] Rushton DH Norris MJ Dover R Busuttil N. Causes of hair loss and the developments in hair rejuvenation. Int J Cosmet Sci (2002) 24(1):17–23. 18498491 10.1046/j.0412-5463.2001.00110.x

[39] Fortes C Mastroeni S Mannooranparampil T Abeni D Panebianco A. Mediterranean Diet: fresh herbs and fresh vegetables decrease the risk of androgenetic alopecia in males. Arch Dermatol Res (2018) 310(1):71–6. 10.1007/s00403-017-1799-z 29181579

[40] Pham CT Romero K Almohanna HM Griggs J Ahmed A Tosti A. The role of diet as an adjuvant treatment in scarring and nonscarring alopecia. Skin Appendage Disord (2020) 6(2):88–96. 32258051 10.1159/000504786 PMC7109385

[41] Walton C King R Rechtman L Kaye W Leray E Marrie RA. Rising prevalence of multiple sclerosis worldwide: insights from the atlas of MS, third edition. Mult Scler (2020) 26(14):1816–21. 10.1177/1352458520970841 PMC7720355 33174475

[42] Montalban X Hauser SL Kappos L Arnold DL Bar-Or A Comi G. Ocrelizumab versus placebo in primary progressive multiple sclerosis. N Engl J Med (2017) 376(3):209–20. 10.1056/NEJMoa1606468 28002688

[43] Hauser SL Bar-Or A Comi G Giovannoni G Hartung H-P Hemmer B. Ocrelizumab versus interferon beta-1a in relapsing multiple sclerosis. N Engl J Med (2017) 376(3):221–34. 10.1056/NEJMoa1601277 28002679

[44] Bar-Or A Li R. Cellular immunology of relapsing multiple sclerosis: interactions, checks, and balances. Lancet Neurol (2021) 20(6):470–83. 10.1016/S1474-4422(21)00063-6 33930317

[45] Obermeier B Mentele R Malotka J Kellermann J Kümpfel T Wekerle H. Matching of oligoclonal immunoglobulin transcriptomes and proteomes of cerebrospinal fluid in multiple sclerosis. Nat Med (2008) 14(6):688–93. 10.1038/nm1714 18488038

[46] Bar-Or A Grove RA Austin DJ Tolson JM VanMeter SA Lewis EW. Subcutaneous ofatumumab in patients with relapsing-remitting multiple sclerosis: the MIRROR study. Neurology (2018) 90(20):e1805–14. 10.1212/WNL.0000000000005516 PMC5957306 29695594

[47] Hauser SL Bar-Or A Cohen JA Comi G Correale J Coyle PK. Ofatumumab versus teriflunomide in multiple sclerosis. N Engl J Med (2020) 383(6):546–57. 10.1056/NEJMoa1917246 32757523

[48] Duddy M Niino M Adatia F Hebert S Freedman M Atkins H. Distinct effector cytokine profiles of memory and naive human b cell subsets and implication in multiple sclerosis. J Immunol (2007) 178(10):6092–9. 10.4049/jimmunol.178.10.6092 17475834

[49] Li R Rezk A Miyazaki Y Hilgenberg E Touil H Shen P. Proinflammatory GM-CSF-producing b cells in multiple sclerosis and b cell depletion therapy. Sci Transl Med (2015) 7(310):310ra166. 10.1126/scitranslmed.aab4176 26491076

[50] Bar-Or A Fawaz L Fan B Darlington PJ Rieger A Ghorayeb C. Abnormal b-cell cytokine responses a trigger of T-cell-mediated disease in MS? Ann Neurol (2010) 67(4):452–61. 10.1002/ana.21939 20437580

[51] Pugliatti M Ferri C. Migration - a route to multiple sclerosis risk globalization? Nat Rev Neurol (2020) 16(2):67–8. 10.1038/s41582-019-0308-8 31907418

[52] Yamout BI Assaad W Tamim H Mrabet S Goueider R. Epidemiology and phenotypes of multiple sclerosis in the middle East north Africa (MENA) region. Mult Scler J Exp Transl Clin (2020) 6(1):2055217319841881. 31984137 10.1177/2055217319841881 PMC6961141

[53] Ghezzi A Deplano V Faroni J Grasso MG Liguori M Marrosu G. Multiple sclerosis in childhood: clinical features of 149 cases. Mult Scler (1997) 3(1):43–6. 10.1177/135245859700300105 9160345

[54] Banwell BL. Pediatric multiple sclerosis. Curr Neurol Neurosci Rep (2004) 4(3):245–52. 10.1007/s11910-004-0045-1 15102351

[55] Fadda G Brown RA Longoni G Castro DA O'Mahony J Verhey LH. MRI And laboratory features and the performance of international criteria in the diagnosis of multiple sclerosis in children and adolescents: a prospective cohort study. Lancet Child Adolesc Health (2018) 2(3):191–204. 30169254 10.1016/S2352-4642(18)30026-9

[56] Berg-Hansen P Celius EG. Socio-economic factors and immigrant population studies of multiple sclerosis. Acta Neurol Scand (2015) 132(199):37–41. 10.1111/ane.12429 26046557

[57] Rotstein DL Marrie RA Maxwell C Gandhi S Schultz SE Fung K. MS risk in immigrants in the McDonald era: a population-based study in Ontario, Canada. Neurology (2019) 93(24):e2203–15. 10.1212/WNL.0000000000008611 PMC6937488 31690681

[58] Mirzaei F. Gestational vitamin d and the risk of multiple sclerosis in offspring. Ann Neurol (2011) 70(1):30–40. 21786297 10.1002/ana.22456 PMC3205990

[59] Goyal MK Johnson TJ Chamberlain JM Casper TC Simmons T Alessandrini EA. Racial and ethnic differences in antibiotic use for viral illness in emergency departments. Pediatrics (2017) 140(4). 10.1542/peds.2017-0203 PMC5613999 28872046

[60] Munger KL Åivo J Hongell K Soilu-Hänninen M Surcel H-M Ascherio A. Vitamin d status during pregnancy and risk of multiple sclerosis in offspring of women in the Finnish maternity cohort. JAMA Neurol (2016) 73(5):515–9. 10.1001/jamaneurol.2015.4800 PMC4861670 26953778

[61] Ahlgren C Lycke J Odén A Andersen O. High risk of MS in Iranian immigrants in gothenburg, Sweden. Mult Scler (2010) 16(9):1079–82. 10.1177/1352458510376777 20670984

[62] Graves JS Chitnis T Weinstock-Guttman B Rubin J Zelikovitch AS Nourbakhsh B. Maternal and perinatal exposures are associated with risk for pediatric-onset multiple sclerosis. Pediatrics (2017) 139(4). 10.1542/peds.2016-2838 PMC5369674 28562303

[63] Nielsen NM Bager P Stenager E Pedersen B Koch-Henriksen N Hjalgrim H. Cesarean section and offspring's risk of multiple sclerosis: a Danish nationwide cohort study. Mult Scler (2013) 19(11):1473–7. 10.1177/1352458513480010 23466398

[64] Maghzi AH Etemadifar M Heshmat-Ghahdarijani K Nonahal S Minagar A Moradi V. Cesarean delivery may increase the risk of multiple sclerosis. Mult Scler (2012) 18(4):468–71. 10.1177/1352458511424904 21982872

[65] Grytten N Torkildsen Ø Aarseth JH Benjaminsen E Celius EG Dahl OP. Month of birth as a latitude-dependent risk factor for multiple sclerosis in Norway. Mult Scler (2013) 19(8):1028–34. 10.1177/1352458512471094 23257620

[66] Torkildsen O Aarseth J Celius EG Holmøy T Kampman MT Løken-Amsrud KI. Reply to comment: month of birth and risk of multiple sclerosis: confounding and adjustments. Ann Clin Transl Neurol (2014) 1(5):376–7. 10.1002/acn3.56 PMC4184690 25356407

[67] Fiddes B Wason J Kemppinen A Ban M Compston A Sawcer S. Confounding underlies the apparent month of birth effect in multiple sclerosis. Ann Neurol (2013) 73(6):714–20. 10.1002/ana.23925 PMC3748787 23744589

[68] Langer-Gould A Smith JB Hellwig K Gonzales E Haraszti S Koebnick C. Breastfeeding, ovulatory years, and risk of multiple sclerosis. Neurology (2017) 89(6):563–9. 10.1212/WNL.0000000000004207 PMC5562955 28701499

[69] Lulu S Graves J Waubant E. Menarche increases relapse risk in pediatric multiple sclerosis. Mult Scler (2016) 22(2):193–200. 25948626 10.1177/1352458515581873 PMC4636485

[70] Munger KL Levin LI O'Reilly EJ Falk KI Ascherio A. Anti-Epstein-Barr virus antibodies as serological markers of multiple sclerosis: a prospective study among united states military personnel. Mult Scler (2011) 17(10):1185–93. 10.1177/1352458511408991 PMC3179777 21685232

[71] Riccio P Rossano R Larocca M Trotta V Mennella I Vitaglione P. Anti-inflammatory nutritional intervention in patients with relapsing-remitting and primary-progressive multiple sclerosis: a pilot study. Exp Biol Med (Maywood) (2016) 241(6):620–35. 10.1177/1535370215618462 PMC4950325 26785711

[72] Fragoso YD Stoney PN McCaffery PJ. The evidence for a beneficial role of vitamin a in multiple sclerosis. CNS Drugs (2014) 28(4):291–9. 10.1007/s40263-014-0148-4 24557746

[73] Munger KL Ascherio A. Prevention and treatment of MS: studying the effects of vitamin d. Mult Scler (2011) 17(12):1405–11. 10.1177/1352458511425366 PMC3351202 21998006

[74] Colotta F Jansson B Bonelli F. Modulation of inflammatory and immune responses by vitamin d. J Autoimmun (2017) 85:78–97. 28733125 10.1016/j.jaut.2017.07.007

[75] Staeva-Vieira TP Freedman LP. 1,25-dihydroxyvitamin D3 inhibits IFN-gamma and IL-4 levels during in vitro polarization of primary murine CD4+ T cells. J Immunol (2002) 168(3):1181–9. 10.4049/jimmunol.168.3.1181 11801653

[76] Lemire JM Adams JS Sakai R Jordan SC. 1 alpha,25-dihydroxyvitamin D3 suppresses proliferation and immunoglobulin production by normal human peripheral blood mononuclear cells. J Clin Invest (1984) 74(2):657–61. 10.1172/JCI111465 PMC370520 6611355

[77] Chen S Sims GP Chen XX Gu YY Chen S Lipsky PE. Modulatory effects of 1,25-dihydroxyvitamin D3 on human b cell differentiation. J Immunol (2007) 179(3):1634–47. 10.4049/jimmunol.179.3.1634 17641030

[78] Lee KN Kang H-S Jeon J-H Kim E-M Yoon S-R Song H. VDUP1 is required for the development of natural killer cells. Immunity (2005) 22(2):195–208. 15723808 10.1016/j.immuni.2004.12.012

[79] Zanatta L Goulart PB Gonçalves R Pierozan P Winkelmann-Duarte EC Woehl VM. 1alpha,25-dihydroxyvitamin D(3) mechanism of action: modulation of l-type calcium channels leading to calcium uptake and intermediate filament phosphorylation in cerebral cortex of young rats. Biochim Biophys Acta (2012) 1823(10):1708–19. doi: 10.1016/j.bbamcr.2012.06.023 22743040

[80] Neveu I Naveilhan P Baudet C Brachet P Metsis M. 1,25-dihydroxyvitamin D3 regulates NT-3, NT-4 but not BDNF mRNA in astrocytes. Neuroreport (1994) 6(1):124–6. 10.1097/00001756-199412300-00032 7703399

[81] Alfredsson L Olsson T. Lifestyle and environmental factors in multiple sclerosis. Cold Spring Harb Perspect Med (2019) 9(4). 10.1101/cshperspect.a028944 PMC6444694 29735578

[82] Hedstrom AK Olsson T Alfredsson L. Body mass index during adolescence, rather than childhood, is critical in determining MS risk. Mult Scler (2016) 22(7):878–83. 10.1177/1352458515603798 26362895

[83] Gianfrancesco MA Stridh P Rhead B Shao X Xu E Graves JS. Evidence for a causal relationship between low vitamin d, high BMI, and pediatric-onset MS. Neurology (2017) 88(17):1623–9. 10.1212/WNL.0000000000003849 PMC5405763 28356466

[84] Langer-Gould A Brara SM Beaber BE Koebnick C. Childhood obesity and risk of pediatric multiple sclerosis and clinically isolated syndrome. Neurology (2013) 80(6):548–52. 10.1212/WNL.0b013e31828154f3 PMC3589288 23365063

[85] Shoelson SE Lee J Goldfine AB. Inflammation and insulin resistance. J Clin Invest (2006) 116(7):1793–801. 10.1172/JCI29069 PMC1483173 16823477

[86] Schreiner TG Genes TM. Obesity and multiple sclerosis-a multifaceted association. J Clin Med (2021) 10(12). 10.3390/jcm10122689 PMC8234028 34207197

[87] Zeyda M Huber J Prager G Stulnig TM. Inflammation correlates with markers of T-cell subsets including regulatory T cells in adipose tissue from obese patients. Obes (Silver Spring) (2011) 19(4):743–8. 10.1038/oby.2010.123 20508627

[88] McLaughlin T Liu L-F Lamendola C Shen L Morton J Rivas H. T-Cell profile in adipose tissue is associated with insulin resistance and systemic inflammation in humans. Arterioscler Thromb Vasc Biol (2014) 34(12):2637–43. 10.1161/ATVBAHA.114.304636 PMC4445971 25341798

[89] Endo Y Asou HK Matsugae N Hirahara K Shinoda K Tumes DJ. Obesity drives Th17 cell differentiation by inducing the lipid metabolic kinase, ACC1. Cell Rep (2015) 12(6):1042–55. 10.1016/j.celrep.2015.07.014 26235623

[90] Deiuliis J Shah Z Shah N Needleman B Mikami D Narula V. Visceral adipose inflammation in obesity is associated with critical alterations in tregulatory cell numbers. PloS One (2011) 6(1):e16376. 21298111 10.1371/journal.pone.0016376 PMC3027666

[91] Correale J Marrodan M. Multiple sclerosis and obesity: the role of adipokines. Front Immunol (2022) 13:1038393. 36457996 10.3389/fimmu.2022.1038393 PMC9705772

[92] Nyirenda MH Fadda G Healy LM Mexhitaj I Poliquin-Lasnier L Hanwell H. Pro-inflammatory adiponectin in pediatric-onset multiple sclerosis. Mult Scler (2021) 27(12):1948–59. 10.1177/1352458521989090 33522403

[93] Signoriello E Lus G Polito R Casertano S Scudiero O Coletta M. Adiponectin profile at baseline is correlated to progression and severity of multiple sclerosis. Eur J Neurol (2019) 26(2):348–55. 10.1111/ene.13822 30300462

[94] Signoriello E Mallardo M Nigro E Polito R Casertano S Di Pietro A. Correction to: adiponectin in cerebrospinal fluid from patients affected by multiple sclerosis is correlated with the progression and severity of disease. Mol Neurobiol (2021) 58(6):2671. 33599955 10.1007/s12035-021-02331-y PMC8496577

[95] Hietaharju A Kuusisto H Nieminen R Vuolteenaho K Elovaara I Moilanen E. Elevated cerebrospinal fluid adiponectin and adipsin levels in patients with multiple sclerosis: a Finnish co-twin study. Eur J Neurol (2010) 17(2):332–4. 10.1111/j.1468-1331.2009.02701.x 19538214

[96] Neumeier M Weigert J Buettner R Wanninger J Schäffler A Müller AM. Detection of adiponectin in cerebrospinal fluid in humans. Am J Physiol Endocrinol Metab (2007) 293(4):E965–9. 10.1152/ajpendo.00119.2007 17623750

[97] Kraszula L Jasińska A Eusebio M-O Kuna P Głąbiński A Pietruczuk M. Evaluation of the relationship between leptin, resistin, adiponectin and natural regulatory T cells in relapsing-remitting multiple sclerosis. Neurol Neurochir Pol (2012) 46(1):22–8. 10.5114/ninp.2012.27211 22426759

[98] Yasui M Ota K. Experimental and clinical studies on dysregulation of magnesium metabolism and the aetiopathogenesis of multiple sclerosis. Magnes Res (1992) 5(4):295–302. 1296766

[99] Hnilicova P Štrbák O Kolisek M Kurča E Zeleňák K Sivák Š. Current methods of magnetic resonance for noninvasive assessment of molecular aspects of pathoetiology in multiple sclerosis. Int J Mol Sci (2020) 21(17). 10.3390/ijms21176117 PMC7504207 32854318

[100] Balto JM Ensari I Hubbard EA Khan N Barnes JL Motl RW. Individual and Co-occurring SNAP risk factors: smoking, nutrition, alcohol consumption, and physical activity in people with multiple sclerosis. Int J MS Care (2016) 18(6):298–304. 27999524 10.7224/1537-2073.2016-040 PMC5166596

[101] Mahad D Lassmann H Turnbull D. Review: mitochondria and disease progression in multiple sclerosis. Neuropathol Appl Neurobiol (2008) 34(6):577–89. 10.1111/j.1365-2990.2008.00987.x PMC2981078 19076696

[102] Altowaijri G Fryman A Yadav V. Correction to: dietary interventions and multiple sclerosis. Curr Neurol Neurosci Rep (2017) 17(12):93. 29038900 10.1007/s11910-017-0804-4

[103] Matveeva O Bogie JFJ Hendriks JJA Linker RA Haghikia A Kleinewietfeld M. Western Lifestyle and immunopathology of multiple sclerosis. Ann N Y Acad Sci (2018) 1417(1):71–86. 29377214 10.1111/nyas.13583 PMC5947729

[104] Timmermans S Bogie JFJ Vanmierlo T Lütjohann D Stinissen P Hellings N. High fat diet exacerbates neuroinflammation in an animal model of multiple sclerosis by activation of the renin angiotensin system. J Neuroimmune Pharmacol (2014) 9(2):209–17. 10.1007/s11481-013-9502-4 24068577

[105] Munger K. A prospective study of serum levels of polyunsaturated fatty acids and effects on multiple sclerosis disease activity and progression. Neurology (2022) 98(18 Supplement).

[106] Bjornevik K Chitnis T Ascherio A Munger KL. Polyunsaturated fatty acids and the risk of multiple sclerosis. Mult Scler (2017) 23(14):1830–8. 10.1177/1352458517691150 PMC5494026 28156186

[107] Chang HY Lee H-N Kim W Surh Y-J. Docosahexaenoic acid induces M2 macrophage polarization through peroxisome proliferator-activated receptor gamma activation. Life Sci (2015) 120:39–47. 25445227 10.1016/j.lfs.2014.10.014

[108] Adolph S Fuhrmann H Schumann J. Unsaturated fatty acids promote the phagocytosis of p. aeruginosa and r. equi by RAW264.7 macrophages. Curr Microbiol (2012) 65(6):649–55. doi: 10.1007/s00284-012-0207-3 22903555

[109] Meital LT Windsor MT Perissiou M Schulze K Magee R Kuballa A. Omega-3 fatty acids decrease oxidative stress and inflammation in macrophages from patients with small abdominal aortic aneurysm. Sci Rep (2019) 9(1):12978. 31506475 10.1038/s41598-019-49362-z PMC6736886

[110] Chiurchiu V Leuti A Dalli J Jacobsson A Battistini L Maccarrone M. Proresolving lipid mediators resolvin D1, resolvin D2, and maresin 1 are critical in modulating T cell responses. Sci Transl Med (2016) 8(353):353ra111. 10.1126/scitranslmed.aaf7483 PMC5149396 27559094

[111] Weinstock-Guttman B Zivadinov R Mahfooz N Carl E Drake A Schneider J. Serum lipid profiles are associated with disability and MRI outcomes in multiple sclerosis. J Neuroinflamm (2011) 8:127. 10.1186/1742-2094-8-127 PMC3228782 21970791

[112] Tettey P Simpson S Taylor B Ponsonby A-L Lucas RM Dwyer T. An adverse lipid profile and increased levels of adiposity significantly predict clinical course after a first demyelinating event. J Neurol Neurosurg Psychiatry (2017) 88(5):395–401. 28320766 10.1136/jnnp-2016-315037

[113] Huang S Rutkowsky JM Snodgrass RG Ono-Moore KD Schneider DA Newman JW. Saturated fatty acids activate TLR-mediated proinflammatory signaling pathways. J Lipid Res (2012) 53(9):2002–13. 10.1194/jlr.D029546 PMC3413240 22766885

[114] Nyirenda MH Morandi E Vinkemeier U Constantin-Teodosiu D Drinkwater S Mee M. TLR2 stimulation regulates the balance between regulatory T cell and Th17 function: a novel mechanism of reduced regulatory T cell function in multiple sclerosis. J Immunol (2015) 194(12):5761–74. 10.4049/jimmunol.1400472 25980006

[115] Reynolds JM Martinez GJ Chung Y Dong C. Toll-like receptor 4 signaling in T cells promotes autoimmune inflammation. Proc Natl Acad Sci U.S.A. (2012) 109(32):13064–9. 10.1073/pnas.1120585109 PMC3420161 22826216

[116] Trotta T Porro C Calvello R Panaro MA. Biological role of toll-like receptor-4 in the brain. J Neuroimmunol (2014) 268(1-2):1–12. 24529856 10.1016/j.jneuroim.2014.01.014

[117] Hadgkiss EJ Jelinek GA Weiland TJ Pereira NG Marck CH Meer DMvd. The association of diet with quality of life, disability, and relapse rate in an international sample of people with multiple sclerosis. Nutr Neurosci (2015) 18(3):125–36. 10.1179/1476830514Y.0000000117 PMC4485697 24628020

[118] Azary S Schreiner T Graves J Waldman A Belman A Guttman BW. Contribution of dietary intake to relapse rate in early paediatric multiple sclerosis. J Neurol Neurosurg Psychiatry (2018) 89(1):28–33. 28993476 10.1136/jnnp-2017-315936 PMC5732893

[119] Fitzgerald KC Tyry T Salter A Cofield SS Cutter G Fox R. Diet quality is associated with disability and symptom severity in multiple sclerosis. Neurology (2018) 90(1):e1–e11. 29212827 10.1212/WNL.0000000000004768

[120] Kleinewietfeld M Manzel A Titze J Kvakan H Yosef N Linker RA. Sodium chloride drives autoimmune disease by the induction of pathogenic TH17 cells. Nature (2013) 496(7446):518–22. 10.1038/nature11868 PMC3746493 23467095

[121] Wu C Yosef N Thalhamer T Zhu C Xiao S Kishi Y. Induction of pathogenic TH17 cells by inducible salt-sensing kinase SGK1. Nature (2013) 496(7446):513–7. 10.1038/nature11984 PMC3637879 23467085

[122] Al KF Craven LJ Gibbons S Parvathy SN Wing AC Graf C. Fecal microbiota transplantation is safe and tolerable in patients with multiple sclerosis: a pilot randomized controlled trial. Mult Scler J Exp Transl Clin (2022) 8(2):20552173221086662. 35571974 10.1177/20552173221086662 PMC9102167

[123] i, M.C.E.a.s.b.u.e M.C. i. Gut microbiome of multiple sclerosis patients and paired household healthy controls reveal associations with disease risk and course. Cell (2022) 185(19):3467–3486.e16. doi: 10.1016/j.cell.2022.08.021 36113426 PMC10143502

[124] Berer K Martínez I Walker A Kunkel B Schmitt-Kopplin P Walter J. Dietary non-fermentable fiber prevents autoimmune neurological disease by changing gut metabolic and immune status. Sci Rep (2018) 8(1):10431. 29993025 10.1038/s41598-018-28839-3 PMC6041322

[125] Rojas OL Pröbstel A-K Porfilio EA Wang AA Charabati M Sun T. Recirculating intestinal IgA-producing cells regulate neuroinflammation via IL-10. Cell (2019) 177(2):492–3. 10.1016/j.cell.2019.03.037 30951673

[126] Probstel AK Zhou X Baumann R Wischnewski S Kutza M Rojas OL. Gut microbiota-specific IgA(+) b cells traffic to the CNS in active multiple sclerosis. Sci Immunol (2020) 5(53). 10.1126/sciimmunol.abc7191 PMC8043673 33219152

[127] Aringer M Costenbader KH Daikh DI Brinks R Mosca M Ramsey-Goldman R. 2019 European league against Rheumatism/American college of rheumatology classification criteria for systemic lupus erythematosus. Arthritis Rheumatol (2019) 71(9):1400–12. 10.1002/art.40930 PMC6827566 31385462

[128] Petri M Orbai A-M Alarcón GS Gordon C Merrill JT Fortin PR. Derivation and validation of the systemic lupus international collaborating clinics classification criteria for systemic lupus erythematosus. Arthritis Rheum (2012) 64(8):2677–86. 10.1002/art.34473 PMC3409311 22553077

[129] Pan L Lu M-P Wang J-H Xu M Yang S-R. Immunological pathogenesis and treatment of systemic lupus erythematosus. World J Pediatr (2020) 16(1):19–30. 30796732 10.1007/s12519-019-00229-3 PMC7040062

[130] Tsokos GC. Systemic lupus erythematosus. N Engl J Med (2011) 365(22):2110–21. 10.1056/NEJMra1100359 22129255

[131] Moulton VR Suarez-Fueyo A Meidan E Li H Mizui M Tsokos GC. Pathogenesis of human systemic lupus erythematosus: a cellular perspective. Trends Mol Med (2017) 23(7):615–35. 10.1016/j.molmed.2017.05.006 PMC5650102 28623084

[132] Cappione A 3rd Anolik JH Pugh-Bernard A Barnard J Dutcher P Silverman G. Germinal center exclusion of autoreactive b cells is defective in human systemic lupus erythematosus. J Clin Invest (2005) 115(11):3205–16. 10.1172/JCI24179 PMC1242189 16211091

[133] Grammer AC Slota R Fischer R Gur H Girschick H Yarboro C. Abnormal germinal center reactions in systemic lupus erythematosus demonstrated by blockade of CD154-CD40 interactions. J Clin Invest (2003) 112(10):1506–20. 10.1172/JCI19301 PMC259134 14617752

[134] Wei C Anolik J Cappione A Zheng B Pugh-Bernard A Brooks J. A new population of cells lacking expression of CD27 represents a notable component of the b cell memory compartment in systemic lupus erythematosus. J Immunol (2007) 178(10):6624–33. 10.4049/jimmunol.178.10.6624 17475894

[135] Crispin JC Liossis SN Kis-Toth K Lieberman LA Kyttaris VC Juang YT. Pathogenesis of human systemic lupus erythematosus: recent advances. Trends Mol Med (2010) 16(2):47–57. 20138006 10.1016/j.molmed.2009.12.005 PMC2823952

[136] Blair PA. CD19(+)CD24(hi)CD38(hi) b cells exhibit regulatory capacity in healthy individuals but are functionally impaired in systemic lupus erythematosus patients. Immunity (2010) 32(1):129–40. 10.1016/j.immuni.2009.11.009 20079667

[137] Puliaeva I Puliaev R Via CS. Therapeutic potential of CD8+ cytotoxic T lymphocytes in SLE. Autoimmun Rev (2009) 8(3):219–23. 10.1016/j.autrev.2008.07.045 PMC3215296 18725326

[138] Crispin JC Vargas MI Alcocer-Varela J. Immunoregulatory T cells in autoimmunity. Autoimmun Rev (2004) 3(2):45–51. 15003187 10.1016/S1568-9972(03)00086-7

[139] Tsokos GC Lo MS Costa Reis P Sullivan KE. New insights into the immunopathogenesis of systemic lupus erythematosus. Nat Rev Rheumatol (2016) 12(12):716–30. 10.1038/nrrheum.2016.186 27872476

[140] Owen KA Price A Ainsworth H Aidukaitis BN Bachali P Catalina MD. Analysis of trans-ancestral SLE risk loci identifies unique biologic networks and drug targets in African and European ancestries. Am J Hum Genet (2020) 107(5):864–81. 10.1016/j.ajhg.2020.09.007 PMC7675009 33031749

[141] Choi MY Costenbader KH. Understanding the concept of pre-clinical autoimmunity: prediction and prevention of systemic lupus erythematosus: identifying risk factors and developing strategies against disease development. Front Immunol (2022) 13:890522. 35720390 10.3389/fimmu.2022.890522 PMC9203849

[142] Webb R Kelly JA Somers EC Hughes T Kaufman KM Sanchez E. Early disease onset is predicted by a higher genetic risk for lupus and is associated with a more severe phenotype in lupus patients. Ann Rheum Dis (2011) 70(1):151–6. 10.1136/ard.2010.141697 PMC3034281 20881011

[143] Dominguez D Kamphuis S Beyene J Wither J Harley JB Blanco I. Relationship between genetic risk and age of diagnosis in systemic lupus erythematosus. J Rheumatol (2021) 48(6):852–8. 10.3899/jrheum.200002 33060314

[144] Kuo CF Grainge MJ Valdes AM See LC Luo SF Yu KH. Familial aggregation of systemic lupus erythematosus and coaggregation of autoimmune diseases in affected families. JAMA Intern Med (2015) 175(9):1518–26. 10.1001/jamainternmed.2015.3528 26193127

[145] Conde PG Farhat LC Braga ALF Sallum AEM Farhat SCL Silva CA. Are prematurity and environmental factors determinants for developing childhood-onset systemic lupus erythematosus? Mod Rheumatol (2018) 28(1):156–60. 10.1080/14397595.2017.1332508 28696177

[146] Trindade VC Carneiro-Sampaio M Bonfa E Silva CA. An update on the management of childhood-onset systemic lupus erythematosus. Paediatr Drugs (2021) 23(4):331–47. 10.1007/s40272-021-00457-z PMC8270778 34244988

[147] Barbhaiya M Costenbader KH. Environmental exposures and the development of systemic lupus erythematosus. Curr Opin Rheumatol (2016) 28(5):497–505. 27428889 10.1097/BOR.0000000000000318 PMC4965307

[148] Javierre BM Fernandez F Richter J Al-Shahrour F Martin-Subero JI Rodriguez-Ubreva J. Changes in the pattern of DNA methylation associate with twin discordance in systemic lupus erythematosus. Genome Res (2010) 20(2):170–9. 10.1101/gr.100289.109 PMC2813473 20028698

[149] Aslani S Mahmoudi M Karami J Jamshidi AR Malekshahi Z Nicknam MH. Epigenetic alterations underlying autoimmune diseases. Autoimmunity (2016) 49(2):69–83. 26761426 10.3109/08916934.2015.1134511

[150] Somers EC Richardson BC. Environmental exposures, epigenetic changes and the risk of lupus. Lupus (2014) 23(6):568–76. 10.1177/0961203313499419 PMC4000546 24763540

[151] Costenbader KH Kim DJ Peerzada J Lockman S Nobles-Knight D Petri M. Cigarette smoking and the risk of systemic lupus erythematosus: a meta-analysis. Arthritis Rheum (2004) 50(3):849–57. 10.1002/art.20049 15022327

[152] Lehmann P Holzle E Kind P Goerz G Plewig G. Experimental reproduction of skin lesions in lupus erythematosus by UVA and UVB radiation. J Am Acad Dermatol (1990) 22(2 Pt 1):181–7. 10.1016/0190-9622(90)70020-i 2179293

[153] Draborg AH Duus K Houen G. Epstein-Barr Virus and systemic lupus erythematosus. Clin Dev Immunol (2012) 2012:370516. 22811739 10.1155/2012/370516 PMC3395176

[154] Schoonen WM Thomas SL Somers EC Smeeth L Kim J Evans S. Do selected drugs increase the risk of lupus? a matched case-control study. Br J Clin Pharmacol (2010) 70(4):588–96. 10.1111/j.1365-2125.2010.03733.x PMC2950993 20840450

[155] Frangou E Chrysanthopoulou A Mitsios A Kambas K Arelaki S Angelidou I. REDD1/autophagy pathway promotes thromboinflammation and fibrosis in human systemic lupus erythematosus (SLE) through NETs decorated with tissue factor (TF) and interleukin-17A (IL-17A). Ann Rheum Dis (2019) 78(2):238–48. 10.1136/annrheumdis-2018-213181 PMC6352428 30563869

[156] Yang F He Y Zhai Z Sun E. Programmed cell death pathways in the pathogenesis of systemic lupus erythematosus. J Immunol Res (2019) 2019:3638562. 31871956 10.1155/2019/3638562 PMC6913273

[157] Leffler J Martin M Gullstrand B Tyden H Lood C Truedsson L. Neutrophil extracellular traps that are not degraded in systemic lupus erythematosus activate complement exacerbating the disease. J Immunol (2012) 188(7):3522–31. 10.4049/jimmunol.1102404 22345666

[158] Al-Mayouf SM Sunker A Abdwani R Abrawi SA Almurshedi F Alhash N. Loss-of-function variant in DNASE1L3 causes a familial form of systemic lupus erythematosus. Nat Genet (2011) 43(12):1186–8. 10.1038/ng.975 22019780

[159] Kamitaki N Sekar A Handsaker RE Rivera H Tooley K Morris DL. Complement genes contribute sex-biased vulnerability in diverse disorders. Nature (2020) 582(7813):577–81. 10.1038/s41586-020-2277-x PMC7319891 32499649

[160] Strickland FM Hewagama A Lu Q Wu A Hinderer R Webb R. Environmental exposure, estrogen and two X chromosomes are required for disease development in an epigenetic model of lupus. J Autoimmun (2012) 38(2-3):J135–43. 10.1016/j.jaut.2011.11.001 PMC3312994 22142890

[161] Nowak K Jablonska E Ratajczak-Wrona W. Immunomodulatory effects of synthetic endocrine disrupting chemicals on the development and functions of human immune cells. Environ Int (2019) 125:350–64. 10.1016/j.envint.2019.01.078 30743143

[162] Noller KL Blair PB O'Brien PC Melton LJ 3rd Offord JR Kaufman RH. Increased occurrence of autoimmune disease among women exposed in utero to diethylstilbestrol. Fertil Steril (1988) 49(6):1080–2. 10.1016/s0015-0282(16)59965-8 3371486

[163] Parks CG D'Aloisio AA Sandler DP. Early life factors associated with adult-onset systemic lupus erythematosus in women. Front Immunol (2016) 7:103. 27064771 10.3389/fimmu.2016.00103 PMC4814765

[164] Parks CG Walitt BT Pettinger M Chen JC Roos AJ Hunt J. Insecticide use and risk of rheumatoid arthritis and systemic lupus erythematosus in the women's health initiative observational study. Arthritis Care Res (Hoboken) (2011) 63(2):184–94. 10.1002/acr.20335 PMC3593584 20740609

[165] Cooper GS Parks CG Treadwell EL St Clair EW Gilkeson GS Dooley MA. Occupational risk factors for the development of systemic lupus erythematosus. J Rheumatol (2004) 31(10):1928–33. 15468355

[166] Mostafalou S Abdollahi M. Pesticides and human chronic diseases: evidences, mechanisms, and perspectives. Toxicol Appl Pharmacol (2013) 268(2):157–77. 10.1016/j.taap.2013.01.025 23402800

[167] Abdollahi M Ranjbar A Shadnia S Nikfar S Rezaie A. Pesticides and oxidative stress: a review. Med Sci Monit (2004) 10(6):RA141–7. 15173684

[168] Gold LS Ward MH Dosemeci M De Roos AJ. Systemic autoimmune disease mortality and occupational exposures. Arthritis Rheum (2007) 56(10):3189–201. 10.1002/art.22880 17907164

[169] Ruiz-Irastorza G Egurbide MV Olivares N Martinez-Berriotxoa A Aguirre C. Vitamin d deficiency in systemic lupus erythematosus: prevalence, predictors and clinical consequences. Rheumatol (Oxford) (2008) 47(6):920–3. 10.1093/rheumatology/ken121 18411213

[170] Borba VZ Vieira JG Kasamatsu T Radominski SC Sato EI Lazaretti-Castro M. Vitamin d deficiency in patients with active systemic lupus erythematosus. Osteoporos Int (2009) 20(3):427–33. 10.1007/s00198-008-0676-1 18600287

[171] Yang CY Leung PS Adamopoulos IE Gershwin ME. The implication of vitamin d and autoimmunity: a comprehensive review. Clin Rev Allergy Immunol (2013) 45(2):217–26. 10.1007/s12016-013-8361-3 PMC6047889 23359064

[172] Luo XY Yang MH Wu FX Wu LJ Chen L Tang Z. Vitamin d receptor gene BsmI polymorphism b allele, but not BB genotype, is associated with systemic lupus erythematosus in a han Chinese population. Lupus (2012) 21(1):53–9. 10.1177/0961203311422709 22004974

[173] Huang CM Wu MC Wu JY Tsai FJ. No association of vitamin d receptor gene start codon fok 1 polymorphisms in Chinese patients with systemic lupus erythematosus. J Rheumatol (2002) 29(6):1211–3. 12064837

[174] Monticielo OA Brenol JC Chies JA Longo MG Rucatti GG Scalco R. The role of BsmI and FokI vitamin d receptor gene polymorphisms and serum 25-hydroxyvitamin d in Brazilian patients with systemic lupus erythematosus. Lupus (2012) 21(1):43–52. 21993390 10.1177/0961203311421798

[175] Handono K Sidarta YO Pradana BA Nugroho RA Hartono IA Kalim H. Vitamin d prevents endothelial damage induced by increased neutrophil extracellular traps formation in patients with systemic lupus erythematosus. Acta Med Indones (2014) 46(3):189–98. 25348181

[176] Terrier B Derian N Schoindre Y Chaara W Geri G Zahr N. Restoration of regulatory and effector T cell balance and b cell homeostasis in systemic lupus erythematosus patients through vitamin d supplementation. Arthritis Res Ther (2012) 14(5):R221. 23075451 10.1186/ar4060 PMC3580532

[177] Borges MC dos Santos Fde M Telles RW Lanna CC Correia MI. Nutritional status and food intake in patients with systemic lupus erythematosus. Nutrition (2012) 28(11-12):1098–103. 10.1016/j.nut.2012.01.015 22898268

[178] Teh P Zakhary B Sandhu VK. The impact of obesity on SLE disease activity: findings from the southern California lupus registry (SCOLR). Clin Rheumatol (2019) 38(2):597–600. 30357495 10.1007/s10067-018-4336-3

[179] Zhao SS Bowes J Barton A Davey Smith G Richardson T. Separating the effects of childhood and adult body size on inflammatory arthritis: a mendelian randomisation study. RMD Open (2022) 8(2). 10.1136/rmdopen-2022-002321 PMC9403135 35995490

[180] Cozier YC Barbhaiya M Castro-Webb N Conte C Tedeschi SK Leatherwood C. Relationship of cigarette smoking and alcohol consumption to incidence of systemic lupus erythematosus in a prospective cohort study of black women. Arthritis Care Res (Hoboken) (2019) 71(5):671–7. 10.1002/acr.23703 PMC6368899 30091287

[181] Fischer K Przepiera-Bedzak H Sawicki M Walecka A Brzosko I Brzosko M. Serum interleukin-23 in polish patients with systemic lupus erythematosus: association with lupus nephritis, obesity, and peripheral vascular disease. Mediators Inflammation (2017) 2017:9401432. 10.1155/2017/9401432 PMC5752988 29430084

[182] Sinicato NA Postal M Peres FA Pelicari Kde O Marini R dos Santos Ade O. Obesity and cytokines in childhood-onset systemic lupus erythematosus. J Immunol Res (2014) 2014:162047. 24741576 10.1155/2014/162047 PMC3987792

[183] Toussirot E Binda D Gueugnon C Dumoulin G. Adiponectin in autoimmune diseases. Curr Med Chem (2012) 19(32):5474–80. 10.2174/092986712803833119 22876925

[184] Dini AA Wang P Ye DQ. Serum adiponectin levels in patients with systemic lupus erythematosus: a meta-analysis. J Clin Rheumatol (2017) 23(7):361–7. 10.1097/RHU.0000000000000580 28937471

[185] Reynolds HR Buyon J Kim M Rivera TL Izmirly P Tunick P. Association of plasma soluble e-selectin and adiponectin with carotid plaque in patients with systemic lupus erythematosus. Atherosclerosis (2010) 210(2):569–74. 10.1016/j.atherosclerosis.2009.12.007 PMC3963602 20044088

[186] Toussirot E Gaugler B Bouhaddi M Nguyen NU Saas P Dumoulin G. Elevated adiponectin serum levels in women with systemic autoimmune diseases. Mediators Inflammation (2010) 2010:938408. 10.1155/2010/938408 PMC3017955 21234350

[187] Lozovoy MA Simao AN Hohmann MS Simao TN Barbosa DS Morimoto HK. Inflammatory biomarkers and oxidative stress measurements in patients with systemic lupus erythematosus with or without metabolic syndrome. Lupus (2011) 20(13):1356–64. 10.1177/0961203311411348 21868433

[188] Patterson SL Schmajuk G Jafri K Yazdany J Katz P. Obesity is independently associated with worse patient-reported outcomes in women with systemic lupus erythematosus. Arthritis Care Res (Hoboken) (2019) 71(1):126–33. 10.1002/acr.23576 PMC6222022 29740985

[189] Kang JH. Obesity increases the incidence of new-onset lupus nephritis and organ damage during follow-up in patients with systemic lupus erythematosus. Lupus (2020) 29(6):578–86. 10.1177/0961203320913616 32208798

[190] Meza-Meza MR Vizmanos-Lamotte B Munoz-Valle JF Parra-Rojas I Garaulet M Campos-Lopez B. Relationship of excess weight with clinical activity and dietary intake deficiencies in systemic lupus erythematosus patients. Nutrients (2019) 11(11). 10.3390/nu11112683 PMC6893805 31698711

[191] Versini M Jeandel PY Rosenthal E Shoenfeld Y. Obesity in autoimmune diseases: not a passive bystander. Autoimmun Rev (2014) 13(9):981–1000. 25092612 10.1016/j.autrev.2014.07.001

[192] Brown K DeCoffe D Molcan E Gibson DL. Diet-induced dysbiosis of the intestinal microbiota and the effects on immunity and disease. Nutrients (2012) 4(8):1095–119. 10.3390/nu4081095 PMC3448089 23016134

[193] Goessler KF Gualano B Nonino CB Bonfa E Nicoletti CF. Lifestyle interventions and weight management in systemic lupus erythematosus patients: a systematic literature review and metanalysis. J Lifestyle Med (2022) 12(1):37–46. 35300036 10.15280/jlm.2022.12.1.37 PMC8918379

[194] Davies RJ Lomer MC Yeo SI Avloniti K Sangle SR D'Cruz DP. Weight loss and improvements in fatigue in systemic lupus erythematosus: a controlled trial of a low glycaemic index diet versus a calorie restricted diet in patients treated with corticosteroids. Lupus (2012) 21(6):649–55. 10.1177/0961203312436854 22311939

[195] Yeh SH Chuang H Lin LW Hsiao CY Eng HL. Regular tai chi chuan exercise enhances functional mobility and CD4CD25 regulatory T cells. Br J Sports Med (2006) 40(3):239–43. 10.1136/bjsm.2005.022095 PMC2491999 16505081

[196] Gleeson M Bishop NC Stensel DJ Lindley MR Mastana SS Nimmo MA. The anti-inflammatory effects of exercise: mechanisms and implications for the prevention and treatment of disease. Nat Rev Immunol (2011) 11(9):607–15. 10.1038/nri3041 21818123

[197] Allison MA Jensky NE Marshall SJ Bertoni AG Cushman M. Sedentary behavior and adiposity-associated inflammation: the multi-ethnic study of atherosclerosis. Am J Prev Med (2012) 42(1):8–13. 22176840 10.1016/j.amepre.2011.09.023 PMC3244676

[198] Legge A Blanchard C Hanly JG. Physical activity, sedentary behaviour and their associations with cardiovascular risk in systemic lupus erythematosus. Rheumatol (Oxford) (2020) 59(5):1128–36. 10.1093/rheumatology/kez429 31691832

[199] Kipen Y Briganti E Strauss B Will R Littlejohn G Morand E. Three year followup of bone mineral density change in premenopausal women with systemic lupus erythematosus. J Rheumatol (1999) 26(2):310–7. 9972964

[200] Alexanderson H Bostrom C. Exercise therapy in patients with idiopathic inflammatory myopathies and systemic lupus erythematosus - a systematic literature review. Best Pract Res Clin Rheumatol (2020) 34(2):101547. 32819833 10.1016/j.berh.2020.101547

[201] Dorner T Furie R. Novel paradigms in systemic lupus erythematosus. Lancet (2019) 393(10188):2344–58. 10.1016/S0140-6736(19)30546-X 31180031

[202] Islam MA Khandker SS Kotyla PJ Hassan R. Immunomodulatory effects of diet and nutrients in systemic lupus erythematosus (SLE): a systematic review. Front Immunol (2020) 11:1477. 32793202 10.3389/fimmu.2020.01477 PMC7387408

[203] de Medeiros MCS Medeiros JCA Medeiros HJ Leitao J Knackfuss MI. Dietary intervention and health in patients with systemic lupus erythematosus: a systematic review of the evidence. Crit Rev Food Sci Nutr (2019) 59(16):2666–73. 10.1080/10408398.2018.1463966 29648479

[204] Choi MY Hahn J Malspeis S Stevens EF Karlson EW Sparks JA. Association of a combination of healthy lifestyle behaviors with reduced risk of incident systemic lupus erythematosus. Arthritis Rheumatol (2022) 74(2):274–83. 10.1002/art.41935 PMC8792100 34313398

[205] Fung TT McCullough ML Newby PK Manson JE Meigs JB Rifai N. Diet-quality scores and plasma concentrations of markers of inflammation and endothelial dysfunction. Am J Clin Nutr (2005) 82(1):163–73. 10.1093/ajcn.82.1.163 16002815

[206] Pocovi-Gerardino G Correa-Rodriguez M Callejas-Rubio JL Rios-Fernandez R Martin-Amada M Cruz-Caparros MG. Beneficial effect of Mediterranean diet on disease activity and cardiovascular risk in systemic lupus erythematosus patients: a cross-sectional study. Rheumatol (Oxford) (2021) 60(1):160–9. 10.1093/rheumatology/keaa210 32594173

[207] Barrea L Muscogiuri G Frias-Toral E Laudisio D Pugliese G Castellucci B. Nutrition and immune system: from the Mediterranean diet to dietary supplementary through the microbiota. Crit Rev Food Sci Nutr (2021) 61(18):3066–90. 10.1080/10408398.2020.1792826 32691606

[208] Makki K Deehan EC Walter J Backhed F. The impact of dietary fiber on gut microbiota in host health and disease. Cell Host Microbe (2018) 23(6):705–15. 10.1016/j.chom.2018.05.012 29902436

[209] Statovci D Aguilera M MacSharry J Melgar S. The impact of Western diet and nutrients on the microbiota and immune response at mucosal interfaces. Front Immunol (2017) 8:838. 28804483 10.3389/fimmu.2017.00838 PMC5532387

[210] Kim M Qie Y Park J Kim CH. Gut microbial metabolites fuel host antibody responses. Cell Host Microbe (2016) 20(2):202–14. 10.1016/j.chom.2016.07.001 PMC4982788 27476413

[211] Kong LC Holmes BA Cotillard A Habi-Rachedi F Brazeilles R Gougis S. Dietary patterns differently associate with inflammation and gut microbiota in overweight and obese subjects. PloS One (2014) 9(10):e109434. 25330000 10.1371/journal.pone.0109434 PMC4203727

[212] Rodriguez-Carrio J Lopez P Sanchez B Gonzalez S Gueimonde M Margolles A. Intestinal dysbiosis is associated with altered short-chain fatty acids and serum-free fatty acids in systemic lupus erythematosus. Front Immunol (2017) 8:23. 28167944 10.3389/fimmu.2017.00023 PMC5253653

[213] Hanninen Kaartinen K Rauma AL Nenonen M Torronen R Hakkinen AS. Antioxidants in vegan diet and rheumatic disorders. Toxicology (2000) 155(1-3):45–53. 11156742 10.1016/s0300-483x(00)00276-6

[214] Hanninen O Nenonen M Ling WH Li DS Sihvonen L. Effects of eating an uncooked vegetable diet for 1 week. Appetite (1992) 19(3):243–54. 10.1016/0195-6663(92)90165-3 1482162

[215] Milovanov Iu S Lysenko LV Milovanova L Dobrosmyslov IA. [The role of balanced low-protein diet in inhibition of predialysis chronic kidney disease progression in patients with systemic diseases]. Ter Arkh (2009) 81(8):52–7. 19799201

[216] Caetano MC Ortiz TT Terreri MT Sarni RO Silva SG Souza FI. Inadequate dietary intake of children and adolescents with juvenile idiopathic arthritis and systemic lupus erythematosus. J Pediatr (Rio J) (2009) 85(6):509–15. 10.2223/JPED.1941 19865782

[217] Liu Y Zhang DT Liu XG. mTOR signaling in T cell immunity and autoimmunity. Int Rev Immunol (2015) 34(1):50–66. 25019278 10.3109/08830185.2014.933957

[218] Gergely P Jr. Grossman C Niland B Puskas F Neupane H Allam F. Mitochondrial hyperpolarization and ATP depletion in patients with systemic lupus erythematosus. Arthritis Rheum (2002) 46(1):175–90. 10.1002/1529-0131(200201)46:1<175::AID-ART10015>3.0.CO;2-H PMC4020417 11817589

[219] Shah D Aggarwal A Bhatnagar A Kiran R Wanchu A. Association between T lymphocyte sub-sets apoptosis and peripheral blood mononuclear cells oxidative stress in systemic lupus erythematosus. Free Radic Res (2011) 45(5):559–67. 10.3109/10715762.2011.555765 21284579

[220] Rodriguez Huerta MD Trujillo-Martin MM Rua-Figueroa I Cuellar-Pompa L Quiros-Lopez R Serrano-Aguilar P. Healthy lifestyle habits for patients with systemic lupus erythematosus: a systemic review. Semin Arthritis Rheum (2016) 45(4):463–70. 10.1016/j.semarthrit.2015.09.003 26522137

[221] Pestka JJ Vines LL Bates MA He K Langoh I. Comparative effects of n-3, n-6 and n-9 unsaturated fatty acid-rich diet consumption on lupus nephritis, autoantibody production and CD4+ T cell-related gene responses in the autoimmune NZBWF1 mouse. PloS One (2014) 9(6):e100255. 24945254 10.1371/journal.pone.0100255 PMC4063768

[222] Dupont J White PJ Carpenter MP Schaefer EJ Meydani SN Elson CE. Food uses and health effects of corn oil. J Am Coll Nutr (1990) 9(5):438–70. 10.1080/07315724.1990.10720403 2258533

[223] Walton AJ Snaith ML Locniskar M Cumberland AG Morrow WJ Isenberg DA. Dietary fish oil and the severity of symptoms in patients with systemic lupus erythematosus. Ann Rheum Dis (1991) 50(7):463–6. 10.1136/ard.50.7.463 PMC1004457 1877851

[224] Wright SA O'Prey FM McHenry MT Leahey WJ Devine AB Duffy EM. A randomised interventional trial of omega-3-polyunsaturated fatty acids on endothelial function and disease activity in systemic lupus erythematosus. Ann Rheum Dis (2008) 67(6):841–8. 10.1136/ard.2007.077156 17875549

[225] Partan RU Hidayat R Saputra N Rahmayani F Prapto H Yudha TW. Seluang fish (Rasbora spp.) oil decreases inflammatory cytokines via increasing vitamin d level in systemic lupus erythematosus. Open Access Maced J Med Sci (2019) 7(9):1418–21. doi: 10.3889/oamjms.2019.308 PMC6542399 31198446

[226] Arriens C Hynan LS Lerman RH Karp DR Mohan C. Placebo-controlled randomized clinical trial of fish oil's impact on fatigue, quality of life, and disease activity in systemic lupus erythematosus. Nutr J (2015) 14:82. 26283629 10.1186/s12937-015-0068-2 PMC4538741

[227] Aparicio-Soto M Sanchez-Hidalgo M Cardeno A Lucena JM Gonzalez-Escribano F Castillo MJ. The phenolic fraction of extra virgin olive oil modulates the activation and the inflammatory response of T cells from patients with systemic lupus erythematosus and healthy donors. Mol Nutr Food Res (2017) 61(8). 10.1002/mnfr.201601080 28198144

[228] Han GM Han XF. Lycopene reduces mortality in people with systemic lupus erythematosus: a pilot study based on the third national health and nutrition examination survey. J Dermatolog Treat (2016) 27(5):430–5. 10.3109/09546634.2015.1133879 26762689

[229] Xu L Zhang L Bertucci AM Pope RM Datta SK. Apigenin, a dietary flavonoid, sensitizes human T cells for activation-induced cell death by inhibiting PKB/Akt and NF-kappaB activation pathway. Immunol Lett (2008) 121(1):74–83. 18812189 10.1016/j.imlet.2008.08.004 PMC2610846

[230] Tam LS Li EK Leung VY Griffith JF Benzie IF Lim PL. Effects of vitamins c and e on oxidative stress markers and endothelial function in patients with systemic lupus erythematosus: a double blind, placebo controlled pilot study. J Rheumatol (2005) 32(2):275–82. 15693087

[231] Comstock GW Burke AE Hoffman SC Helzlsouer KJ Bendich A Masi AT. Serum concentrations of alpha tocopherol, beta carotene, and retinol preceding the diagnosis of rheumatoid arthritis and systemic lupus erythematosus. Ann Rheum Dis (1997) 56(5):323–5. 10.1136/ard.56.5.323 PMC1752374 9175934

[232] Minami Y Sasaki T Arai Y Kurisu Y Hisamichi S. Diet and systemic lupus erythematosus: a 4 year prospective study of Japanese patients. J Rheumatol (2003) 30(4):747–54. 12672194

[233] Molad Y Rachmilewitz B Sidi Y Pinkhas J Weinberger A. Serum cobalamin and transcobalamin levels in systemic lupus erythematosus. Am J Med (1990) 88(2):141–4. 10.1016/0002-9343(90)90463-n 2301440

[234] McKinley MC. Nutritional aspects and possible pathological mechanisms of hyperhomocysteinaemia: an independent risk factor for vascular disease. Proc Nutr Soc (2000) 59(2):221–37. 10.1017/s0029665100000252 10946791

[235] Bronstrup A Hages M Prinz-Langenohl R Pietrzik K. Effects of folic acid and combinations of folic acid and vitamin b-12 on plasma homocysteine concentrations in healthy, young women. Am J Clin Nutr (1998) 68(5):1104–10. 10.1093/ajcn/68.5.1104 9808229

[236] Maggini S Wintergerst ES Beveridge S Hornig DH. Selected vitamins and trace elements support immune function by strengthening epithelial barriers and cellular and humoral immune responses. Br J Nutr (2007) 98(Suppl 1):S29–35. 10.1017/S0007114507832971 17922955

[237] Selhub J Jacques PF Rosenberg IH Rogers G Bowman BA Gunter EW. Serum total homocysteine concentrations in the third national health and nutrition examination survey (1991-1994): population reference ranges and contribution of vitamin status to high serum concentrations. Ann Intern Med (1999) 131(5):331–9. 10.7326/0003-4819-131-5-199909070-00003 10475885

[238] Lourdudoss C Elkan AC Hafström I Jogestrand T Gustafsson T van Vollenhoven R. Dietary micronutrient intake and atherosclerosis in systemic lupus erythematosus. Lupus (2016) 25(14):1602–9. 10.1177/0961203316655211 27334936

[239] Erkelens MN Mebius RE. Retinoic acid and immune homeostasis: a balancing act. Trends Immunol (2017) 38(3):168–80. 10.1016/j.it.2016.12.006 28094101

[240] Duriancik DM Hoag KA. Vitamin a deficiency alters splenic dendritic cell subsets and increases CD8(+)Gr-1(+) memory T lymphocytes in C57BL/6J mice. Cell Immunol (2010) 265(2):156–63. 10.1016/j.cellimm.2010.08.006 PMC2966341 20832059

[241] Huang Z Liu Y Qi G Brand D Zheng SG. Role of vitamin a in the immune system. J Clin Med (2018) 7(9). 10.3390/jcm7090258 PMC6162863 30200565

[242] Mucida D Park Y Kim G Turovskaya O Scott I Kronenberg M. Reciprocal TH17 and regulatory T cell differentiation mediated by retinoic acid. Science (2007) 317(5835):256–60. 10.1126/science.1145697 17569825

[243] Handono K Firdausi SN Pratama MZ Endharti AT Kalim H. Vitamin a improve Th17 and treg regulation in systemic lupus erythematosus. Clin Rheumatol (2016) 35(3):631–8. 10.1007/s10067-016-3197-x 26852315

[244] Shah K Lee WW Lee SH Kim SH Kang SW Craft J. Dysregulated balance of Th17 and Th1 cells in systemic lupus erythematosus. Arthritis Res Ther (2010) 12(2):R53. doi: 10.1186/ar2964 20334681 PMC2888202

[245] Shin MS Lee N Kang I. Effector T-cell subsets in systemic lupus erythematosus: update focusing on Th17 cells. Curr Opin Rheumatol (2011) 23(5):444–8. 10.1097/BOR.0b013e328349a255 PMC3489922 21720245

[246] Scrivo R Massaro L Barbati C Vomero M Ceccarelli F Spinelli FR. The role of dietary sodium intake on the modulation of T helper 17 cells and regulatory T cells in patients with rheumatoid arthritis and systemic lupus erythematosus. PloS One (2017) 12(9):e0184449. 28877244 10.1371/journal.pone.0184449 PMC5587319

[247] Correa-Rodriguez M DelOlmo-Romero S Pocovi-Gerardino G Callejas-Rubio JL Rios-Fernandez R Ortego-Centeno N. Dietary sodium, potassium, and sodium to potassium ratio in patients with systemic lupus erythematosus. Biol Res Nurs (2022) 24(2):235–44. 10.1177/10998004211065491 34978207

[248] Hevia A Milani C Lopez P Cuervo A Arboleya S Duranti S. Intestinal dysbiosis associated with systemic lupus erythematosus. mBio (2014) 5(5):e01548–14. 10.1128/mBio.01548-14 PMC4196225 25271284

[249] Manfredo Vieira S Hiltensperger M Kumar V Zegarra-Ruiz D Dehner C Khan N. Translocation of a gut pathobiont drives autoimmunity in mice and humans. Science (2018) 359(6380):1156–61. 10.1126/science.aar7201 PMC5959731 29590047

[250] Zhao Z Ren J Dai C Kannapell CC Wang H Gaskin F. Nature of T cell epitopes in lupus antigens and HLA-DR determines autoantibody initiation and diversification. Ann Rheum Dis (2019) 78(3):380–90. 10.1136/annrheumdis-2018-214125 PMC6377330 30254034

[251] Choi SC Brown J Gong M Ge Y Zadeh M Li W. Gut microbiota dysbiosis and altered tryptophan catabolism contribute to autoimmunity in lupus-susceptible mice. Sci Transl Med (2020) 12(551). 10.1126/scitranslmed.aax2220 PMC7739186 32641487

[252] Konieczna P Akdis CA Quigley EM Shanahan F O'Mahony L. Portrait of an immunoregulatory bifidobacterium. Gut Microbes (2012) 3(3):261–6. 10.4161/gmic.20358 PMC3427218 22572827

[253] Zhao Q Elson CO. Adaptive immune education by gut microbiota antigens. Immunology (2018) 154(1):28–37. 29338074 10.1111/imm.12896 PMC5904715

[254] Blank M Barzilai O Shoenfeld Y. Molecular mimicry and auto-immunity. Clin Rev Allergy Immunol (2007) 32(1):111–8. 10.1007/BF02686087 17426366

[255] Pisetsky DS. The role of bacterial DNA in autoantibody induction. Curr Top Microbiol Immunol (2000) 247:143–55. 10.1007/978-3-642-59672-8_10 10689785

[256] Kotzin BL Kozora E. Anti-DNA meets NMDA in neuropsychiatric lupus. Nat Med (2001) 7(11):1175–6. 10.1038/nm1101-1175 11689873

[257] Wang X Shu Q Song L Liu Q Qu X Li M. Gut microbiota in systemic lupus erythematosus and correlation with diet and clinical manifestations. Front Med (Lausanne) (2022) 9:915179. 35847775 10.3389/fmed.2022.915179 PMC9279557

[258] Wlodarska M Willing BP Bravo DM Finlay BB. Phytonutrient diet supplementation promotes beneficial clostridia species and intestinal mucus secretion resulting in protection against enteric infection. Sci Rep (2015) 5:9253. 25787310 10.1038/srep09253 PMC4365398

[259] Zegarra-Ruiz DF El Beidaq A Iniguez AJ Lubrano Di Ricco M ManfredoVieira S Ruff WE. A diet-sensitive commensal lactobacillus strain mediates TLR7-dependent systemic autoimmunity. Cell Host Microbe (2019) 25(1):113–127.e6. 30581114 10.1016/j.chom.2018.11.009 PMC6377154

[260] Khorasani S Mahmoudi M Kalantari MR Lavi Arab F Esmaeili SA Mardani F. Amelioration of regulatory T cells by lactobacillus delbrueckii and lactobacillus rhamnosus in pristane-induced lupus mice model. J Cell Physiol (2019) 234(6):9778–86. 10.1002/jcp.27663 30370554

[261] Mu Q Zhang H Liao X Lin K Liu H Edwards MR. Control of lupus nephritis by changes of gut microbiota. Microbiome (2017) 5(1):73. 28697806 10.1186/s40168-017-0300-8 PMC5505136

[262] Rajabi F Drake LA Senna MM Rezaei N. Alopecia areata: a review of disease pathogenesis. Br J Dermatol (2018) 179(5):1033–48. 10.1111/bjd.16808 29791718

[263] Paus R Ito N Takigawa M Ito T. The hair follicle and immune privilege. J Investig Dermatol Symp Proc (2003) 8(2):188–94. 10.1046/j.1087-0024.2003.00807.x 14582671

[264] Alkhalifah A Alsantali A Wang E McElwee KJ Shapiro J. Alopecia areata update: part II. treatment. J Am Acad Dermatol (2010) 62(2):191–202, quiz 203-4. doi: 10.1016/j.jaad.2009.10.031 20115946

[265] Zhou C Li X Wang C Zhang J. Alopecia areata: an update on etiopathogenesis, diagnosis, and management. Clin Rev Allergy Immunol (2021) 61(3):403–23. 10.1007/s12016-021-08883-0 34403083

[266] Pratt CH King LE Jr Messenger AG. Alopecia areata. Nat Rev Dis Primers (2017) 3:17011. 28300084 10.1038/nrdp.2017.11 PMC5573125

[267] Petukhova L Christiano AM. Functional interpretation of genome-wide association study evidence in alopecia areata. J Invest Dermatol (2016) 136(1):314–7. 10.1038/JID.2015.402 PMC4870380 26763452

[268] Bertolini M McElwee K Gilhar A Bulfone-Paus S Paus R. Hair follicle immune privilege and its collapse in alopecia areata. Exp Dermatol (2020) 29(8):703–25. 10.1111/exd.14155 32682334

[269] Jang YH Choi JK Jang YH Moon SY Lee WJ Lee SJ. Increased blood levels of NKG2D(+)CD4(+) T cells in patients with alopecia areata. J Am Acad Dermatol (2017) 76(1):151–3. 10.1016/j.jaad.2016.07.056 27986136

[270] Subramanya RD Coda AB Sinha AA. Transcriptional profiling in alopecia areata defines immune and cell cycle control related genes within disease-specific signatures. Genomics (2010) 96(3):146–53. 10.1016/j.ygeno.2010.05.002 20546884

[271] Dai Z Xing L Cerise J Wang EH Jabbari A de Jong A. CXCR3 blockade inhibits T cell migration into the skin and prevents development of alopecia areata. J Immunol (2016) 197(4):1089–99. 10.4049/jimmunol.1501798 PMC5031416 27412416

[272] Bystryn JC Orentreich N Stengel F. Direct immunofluorescence studies in alopecia areata and male pattern alopecia. J Invest Dermatol (1979) 73(5):317–20. 10.1111/1523-1747.ep12549703 387883

[273] Tobin DJ. Characterization of hair follicle antigens targeted by the anti-hair follicle immune response. J Investig Dermatol Symp Proc (2003) 8(2):176–81. 10.1046/j.1087-0024.2003.00805.x 14582669

[274] Paus R Slominski A Czarnetzki BM. Is alopecia areata an autoimmune-response against melanogenesis-related proteins, exposed by abnormal MHC class I expression in the anagen hair bulb? Yale J Biol Med (1993) 66(6):541–54. PMC2588848 7716973

[275] Barton VR Toussi A Awasthi S Kiuru M. Treatment of pediatric alopecia areata: a systematic review. J Am Acad Dermatol (2022) 86(6):1318–34. 10.1016/j.jaad.2021.04.077 PMC8556406 33940103

[276] Muller SA Winkelmann RK. Alopecia areata. an evaluation of 736 patients. Arch Dermatol (1963) 88:290–7. 10.1001/archderm.1963.01590210048007 14043621

[277] Villasante Fricke AC Miteva M. Epidemiology and burden of alopecia areata: a systematic review. Clin Cosmet Investig Dermatol (2015) 8:397–403. doi: 10.2147/CCID.S53985 PMC4521674 26244028

[278] Ito T. Advances in the management of alopecia areata. J Dermatol (2012) 39(1):11–7. 10.1111/j.1346-8138.2011.01476.x 22211297

[279] Gilhar A Etzioni A Paus R. Alopecia areata. N Engl J Med (2012) 366(16):1515–25. 10.1056/NEJMra1103442 22512484

[280] Tembhre MK Sharma VK. T-Helper and regulatory T-cell cytokines in the peripheral blood of patients with active alopecia areata. Br J Dermatol (2013) 169(3):543–8. 10.1111/bjd.12396 23607748

[281] Suarez-Farinas M Ungar B Noda S Shroff A Mansouri Y Fuentes-Duculan J. Alopecia areata profiling shows TH1, TH2, and IL-23 cytokine activation without parallel TH17/TH22 skewing. J Allergy Clin Immunol (2015) 136(5):1277–87. 10.1016/j.jaci.2015.06.032 26316095

[282] Kurien BT Hensley K Bachmann M Scofield RH. Oxidatively modified autoantigens in autoimmune diseases. Free Radic Biol Med (2006) 41(4):549–56. 10.1016/j.freeradbiomed.2006.05.020 16863987

[283] Tobin DJ. Morphological analysis of hair follicles in alopecia areata. Microsc Res Tech (1997) 38(4):443–51. 10.1002/(SICI)1097-0029(19970815)38:4<443::AID-JEMT12>3.0.CO;2-J 9297694

[284] Yenin JZ Serarslan G Yonden Z Ulutas KT. Investigation of oxidative stress in patients with alopecia areata and its relationship with disease severity, duration, recurrence and pattern. Clin Exp Dermatol (2015) 40(6):617–21. 10.1111/ced.12556 25524272

[285] Sachdeva S Khurana A Goyal P Sardana K. Does oxidative stress correlate with disease activity and severity in alopecia areata? an analytical study. J Cosmet Dermatol (2022) 21(4):1629–34. 10.1111/jocd.14253 34037317

[286] Ozturk P Arican O Kurutas EB Mulayim K. Oxidative stress biomarkers and adenosine deaminase over the alopecic area of the patients with alopecia areata. Balkan Med J (2016) 33(2):188–92. 10.5152/balkanmedj.2016.16190 PMC4924963 27403388

[287] Acharya P Mathur MC. Oxidative stress in alopecia areata: a systematic review and meta-analysis. Int J Dermatol (2020) 59(4):434–40. 10.1111/ijd.14753 31875951

[288] Peters JB Warren MP. Reversible alopecia associated with high blood mercury levels and early menopause: a report of two cases. Menopause (2019) 26(8):915–8. 10.1097/GME.0000000000001332 30939539

[289] Pigatto PD Ferrucci SM Brambilla L Guzzi G. Alopecia areata and toxic metals. Skin Appendage Disord (2020) 6(3):177–9. 10.1159/000507296 PMC7325209 32656240

[290] Lim CP Severin RK Petukhova L. Big data reveal insights into alopecia areata comorbidities. J Investig Dermatol Symp Proc (2018) 19(1):S57–61. 10.1016/j.jisp.2017.10.006 PMC5862393 29273109

[291] Safina DD Abdulkhakov RA Abdulkhakov SR Odintsova A Cheremina NA. [Clinical case of a combination of ulcerative colitis and alopecia areata]. Eksp Klin Gastroenterol (2013) 2013(12):92–6. 24933997

[292] Moreno-Arrones OM Serrano-Villar S Perez-Brocal V Saceda-Corralo D Morales-Raya C Rodrigues-Barata R. Analysis of the gut microbiota in alopecia areata: identification of bacterial biomarkers. J Eur Acad Dermatol Venereol (2020) 34(2):400–5. 10.1111/jdv.15885 31419351

[293] Rebello D Wang E Yen E Lio PA Kelly CR. Hair growth in two alopecia patients after fecal microbiota transplant. ACG Case Rep J (2017) 4:e107. 28932754 10.14309/crj.2017.107 PMC5599691

[294] Bertolini M Gilhar A Paus R. Alopecia areata as a model for T cell-dependent autoimmune diseases. Exp Dermatol (2012) 21(6):477–9. 10.1111/j.1600-0625.2011.01427.x 22621196

[295] Xie Z Komuves L Yu QC Elalieh H Ng DC Leary C. Lack of the vitamin d receptor is associated with reduced epidermal differentiation and hair follicle growth. J Invest Dermatol (2002) 118(1):11–6. 10.1046/j.1523-1747.2002.01644.x 11851870

[296] Malloy PJ Pike JW Feldman D. The vitamin d receptor and the syndrome of hereditary 1,25-dihydroxyvitamin d-resistant rickets. Endocr Rev (1999) 20(2):156–88. doi: 10.1210/edrv.20.2.0359 10204116

[297] Marx SJ Spiegel AM Brown EM Gardner DG Downs RW Jr Attie M. A familial syndrome of decrease in sensitivity to 1,25-dihydroxyvitamin d. J Clin Endocrinol Metab (1978) 47(6):1303–10. 10.1210/jcem-47-6-1303 233695

[298] Cerman AA Solak SS Altunay I Kucukunal NA. Topical calcipotriol therapy for mild-to-Moderate alopecia areata: a retrospective study. J Drugs Dermatol (2015) 14(6):616–20. 26091388

[299] Seleit I Bakry OA Badr E Hassan EH. Vitamin d receptor gene polymorphism in chronic telogen effluvium; a case-control study. Clin Cosmet Investig Dermatol (2019) 12:745–50. 10.2147/CCID.S227232 PMC6790134 31632122

[300] Aksu Cerman A Sarikaya Solak S Kivanc Altunay I. Vitamin d deficiency in alopecia areata. Br J Dermatol (2014) 170(6):1299–304. 10.1111/bjd.12980 24655364

[301] Gade VKV Mony A Munisamy M Chandrashekar L Rajappa M. An investigation of vitamin d status in alopecia areata. Clin Exp Med (2018) 18(4):577–84. 10.1007/s10238-018-0511-8 29869122

[302] Morinaga H Mohri Y Grachtchouk M Asakawa K Matsumura H Oshima M. Obesity accelerates hair thinning by stem cell-centric converging mechanisms. Nature (2021) 595(7866):266–71. 10.1038/s41586-021-03624-x PMC9600322 34163066

[303] Fessatou S Kostaki M Karpathios T. Coeliac disease and alopecia areata in childhood. J Paediatr Child Health (2003) 39(2):152–4. 10.1046/j.1440-1754.2003.00116.x 12603809

[304] Barbato M Viola F Grillo R Franchin L Lo Russo L Lucarelli S. Alopecia and coeliac disease: report of two patients showing response to gluten-free diet. Clin Exp Dermatol (1998) 23(5):236–7. 10.1046/j.1365-2230.1998.00357.x 10233613

[305] Garg S Sangwan A. Dietary protein deficit and deregulated autophagy: a new clinico-diagnostic perspective in pathogenesis of early aging, skin, and hair disorders. Indian Dermatol Online J (2019) 10(2):115–24. 10.4103/idoj.IDOJ_123_18 PMC6434747 30984584

[306] Freinkel RK Freinkel N. Hair growth and alopecia in hypothyroidism. Arch Dermatol (1972) 106(3):349–52. 5055094

[307] Thompson JM Mirza MA Park MK Qureshi AA Ch E. The role of micronutrients in alopecia areata: a review. Am J Clin Dermatol (2017) 18(5):663–79. 10.1007/s40257-017-0285-x PMC5685931 28508256

[308] McElwee KJ Niiyama S Freyschmidt-Paul P Wenzel E Kissling S Sundberg JP. Dietary soy oil content and soy-derived phytoestrogen genistein increase resistance to alopecia areata onset in C3H/HeJ mice. Exp Dermatol (2003) 12(1):30–6. 10.1034/j.1600-0625.2003.120104.x 12631244

[309] Simakou T Butcher JP Reid S Henriquez FL. Alopecia areata: a multifactorial autoimmune condition. J Autoimmun (2019) 98:74–85. 30558963 10.1016/j.jaut.2018.12.001

[310] Harada N Okajima K Arai M Kurihara H Nakagata N. Administration of capsaicin and isoflavone promotes hair growth by increasing insulin-like growth factor-I production in mice and in humans with alopecia. Growth Horm IGF Res (2007) 17(5):408–15. 10.1016/j.ghir.2007.04.009 17569567

[311] Messina M Messina V. Soyfoods, soybean isoflavones, and bone health: a brief overview. J Ren Nutr (2000) 10(2):63–8. 10.1016/s1051-2276(00)90001-3 10757817

[312] Prie BE Voiculescu VM Ionescu-Bozdog OB Petrutescu B Iosif L Gaman LE. Oxidative stress and alopecia areata. J Med Life (2015) 8 Spec Issue(Spec Issue):43–6. PMC4564047 26361510

[313] Kantor J Kessler LJ Brooks DG Cotsarelis G. Decreased serum ferritin is associated with alopecia in women. J Invest Dermatol (2003) 121(5):985–8. 10.1046/j.1523-1747.2003.12540.x 14708596

[314] Kalkan G Yigit S Karakus N Ates O Bozkurt N Ozdemir A. Methylenetetrahydrofolate reductase C677T mutation in patients with alopecia areata in Turkish population. Gene (2013) 530(1):109–12. 10.1016/j.gene.2013.08.016 23954881

[315] Klotz L Farkas M Bain N Keskitalo S Semmler A Ineichen B. The variant methylenetetrahydrofolate reductase c.1298A>C (p.E429A) is associated with multiple sclerosis in a German case-control study. Neurosci Lett (2010) 468(3):183–5. doi: 10.1016/j.neulet.2009.10.057 19854238

[316] Zhou HY Yuan M. MTHFR polymorphisms (rs1801133) and systemic lupus erythematosus risk: a meta-analysis. Med (Baltimore) (2020) 99(40):e22614. 10.1097/MD.0000000000022614 PMC7535654 33019481

[317] Weismann K Hagdrup HK. Hair changes due to zinc deficiency in a case of sucrose malabsorption. Acta Derm Venereol (1981) 61(5):444–7. 6172938

[318] Trost LB Bergfeld WF Calogeras E. The diagnosis and treatment of iron deficiency and its potential relationship to hair loss. J Am Acad Dermatol (2006) 54(5):824–44. 10.1016/j.jaad.2005.11.1104 16635664

[319] Aydingoz IE Ferhanoglu B Guney O. Does tissue iron status have a role in female alopecia? J Eur Acad Dermatol Venereol (1999) 13(1):65–7. 10.1111/j.1468-3083.1999.tb00849.x 10565636

[320] White MI Currie J Williams MP. A study of the tissue iron status of patients with alopecia areata. Br J Dermatol (1994) 130(2):261–3. 10.1111/j.1365-2133.1994.tb02917.x 8123587

[321] Rushton DH Ramsay ID. The importance of adequate serum ferritin levels during oral cyproterone acetate and ethinyl oestradiol treatment of diffuse androgen-dependent alopecia in women. Clin Endocrinol (Oxf) (1992) 36(4):421–7. 10.1111/j.1365-2265.1992.tb01470.x 1424176

[322] Duriancik DM Lackey DE Hoag KA. Vitamin a as a regulator of antigen presenting cells. J Nutr (2010) 140(8):1395–9. 10.3945/jn.110.124461 20554902

[323] Buck J Derguini F Levi E Nakanishi K Hammerling U. Intracellular signaling by 14-hydroxy-4,14-retro-retinol. Science (1991) 254(5038):1654–6. 10.1126/science.1749937 1749937

[324] Geissmann F Revy P Brousse N Lepelletier Y Folli C Durandy A. Retinoids regulate survival and antigen presentation by immature dendritic cells. J Exp Med (2003) 198(4):623–34. 10.1084/jem.20030390 PMC2194172 12925678

[325] Blomhoff HK Smeland EB Erikstein B Rasmussen AM Skrede B Skjonsberg C. Vitamin a is a key regulator for cell growth, cytokine production, and differentiation in normal b cells. J Biol Chem (1992) 267(33):23988–92. 1429735

[326] Naziroglu M Kokcam I. Antioxidants and lipid peroxidation status in the blood of patients with alopecia. Cell Biochem Funct (2000) 18(3):169–73. 10.1002/1099-0844(200009)18:3<169::AID-CBF870>3.0.CO;2-T 10965354

[327] Shih MY Kane MA Zhou P Yen CL Streeper RS Napoli JL. Retinol esterification by DGAT1 is essential for retinoid homeostasis in murine skin. J Biol Chem (2009) 284(7):4292–9. 10.1074/jbc.M807503200 PMC2640966 19028692

[328] Duncan FJ Silva KA Johnson CJ King BL Szatkiewicz JP Kamdar SP. Endogenous retinoids in the pathogenesis of alopecia areata. J Invest Dermatol (2013) 133(2):334–43. 10.1038/jid.2012.344 PMC3546144 23014334

[329] Rangu S Lee JJ Hu W Bittinger K Castelo-Soccio L. Understanding the gut microbiota in pediatric patients with alopecia areata and their siblings: a pilot study. JID Innov (2021) 1(4):100051. 34909748 10.1016/j.xjidi.2021.100051 PMC8659389

[330] Borde A Astrand A. Alopecia areata and the gut-the link opens up for novel therapeutic interventions. Expert Opin Ther Targets (2018) 22(6):503–11. 10.1080/14728222.2018.1481504 29808708

[331] Lerner A Matthias T. Changes in intestinal tight junction permeability associated with industrial food additives explain the rising incidence of autoimmune disease. Autoimmun Rev (2015) 14(6):479–89. 10.1016/j.autrev.2015.01.009 25676324

[332] Blossom SJ Doss JC Gilbert KM. Chronic exposure to a trichloroethylene metabolite in autoimmune-prone MRL+/+ mice promotes immune modulation and alopecia. Toxicol Sci (2007) 95(2):401–11. doi: 10.1093/toxsci/kfl149 17077186

[333] Buckley JP Kim H Wong E Rebholz CM. Ultra-processed food consumption and exposure to phthalates and bisphenols in the US national health and nutrition examination survey, 2013-2014. Environ Int (2019) 131:105057. 31398592 10.1016/j.envint.2019.105057 PMC6728187

[334] Morris DL Sheng Y Zhang Y Wang YF Zhu Z Tombleson P. Genome-wide association meta-analysis in Chinese and European individuals identifies ten new loci associated with systemic lupus erythematosus. Nat Genet (2016) 48(8):940–6. 10.1038/ng.3603 PMC4966635 27399966

[335] Wang YF Zhang Y Lin Z Zhang H Wang TY Cao Y. Identification of 38 novel loci for systemic lupus erythematosus and genetic heterogeneity between ancestral groups. Nat Commun (2021) 12(1):772. 33536424 10.1038/s41467-021-21049-y PMC7858632

[336] Dalton CM Brex PA Jenkins R Fox NC Miszkiel KA Crum WR. Progressive ventricular enlargement in patients with clinically isolated syndromes is associated with the early development of multiple sclerosis. J Neurol Neurosurg Psychiatry (2002) 73(2):141–7. 10.1136/jnnp.73.2.141 PMC1737988 12122170

[337] Li QZ Karp DR Quan J Branch VK Zhou J Lian Y. Risk factors for ANA positivity in healthy persons. Arthritis Res Ther (2011) 13(2):R38. 21366908 10.1186/ar3271 PMC3132017

[338] Satoh M Chan EK Ho LA Rose KM Parks CG Cohn RD. Prevalence and sociodemographic correlates of antinuclear antibodies in the united states. Arthritis Rheum (2012) 64(7):2319–27. 10.1002/art.34380 PMC3330150 22237992

[339] Agre K McCarthy Veach P Bemmels H Wiens K LeRoy BS Hordinsky M. Familial implications of autoimmune disease: recurrence risks of alopecia areata and associated conditions in first-degree relatives. J Genet Couns (2020) 29(1):35–43. 31605426 10.1002/jgc4.1178

[340] Ramos PS Brown EE Kimberly RP Langefeld CD. Genetic factors predisposing to systemic lupus erythematosus and lupus nephritis. Semin Nephrol (2010) 30(2):164–76. 10.1016/j.semnephrol.2010.01.007 PMC2847514 20347645

[341] Kinkel RP Dontchev M Kollman C Skaramagas TT O'Connor PW Simon JH. Association between immediate initiation of intramuscular interferon beta-1a at the time of a clinically isolated syndrome and long-term outcomes: a 10-year follow-up of the controlled high-risk avonex multiple sclerosis prevention study in ongoing neurological surveillance. Arch Neurol (2012) 69(2):183–90. 10.1001/archneurol.2011.1426 21987393

[342] De Jager PL Chibnik LB Cui J Reischl J Lehr S Simon KC. Integration of genetic risk factors into a clinical algorithm for multiple sclerosis susceptibility: a weighted genetic risk score. Lancet Neurol (2009) 8(12):1111–9. 10.1016/S1474-4422(09)70275-3 PMC3099419 19879194

[343] Xia Z Steele SU Bakshi A Clarkson SR White CC Schindler MK. Assessment of early evidence of multiple sclerosis in a prospective study of asymptomatic high-risk family members. JAMA Neurol (2017) 74(3):293–300. 28114441 10.1001/jamaneurol.2016.5056 PMC5348267

[344] Xia Z White CC Owen EK Von Korff A Clarkson SR McCabe CA. Genes and environment in multiple sclerosis project: a platform to investigate multiple sclerosis risk. Ann Neurol (2016) 79(2):178–89. 10.1002/ana.24560 PMC4778957 26583565

[345] Rewers M Bugawan TL Norris JM Blair A Beaty B Hoffman M. Newborn screening for HLA markers associated with IDDM: diabetes autoimmunity study in the young (DAISY). Diabetologia (1996) 39(7):807–12. 10.1007/s001250050514 8817105

[346] Young KA Munroe ME Guthridge JM Kamen DL Gilkensen GS Harley JB. Screening characteristics for enrichment of individuals at higher risk for transitioning to classified SLE. Lupus (2019) 28(5):597–606. 30845880 10.1177/0961203319834675 PMC6506346

[347] Cui J Malspeis S Choi MY Lu B Sparks JA Yoshida K. Risk prediction models for incident systemic lupus erythematosus among women in the nurses' health study cohorts using genetics, family history, and lifestyle and environmental factors. Semin Arthritis Rheum (2023) 58:152143. 36481507 10.1016/j.semarthrit.2022.152143 PMC9840676

[348] Munroe ME Young KA Kamen DL Guthridge JM Niewold TB Costenbader KH. Discerning risk of disease transition in relatives of systemic lupus erythematosus patients utilizing soluble mediators and clinical features. Arthritis Rheumatol (2017) 69(3):630–42. 10.1002/art.40004 PMC5329053 27863174

[349] Cui J Raychaudhuri S Karlson EW Speyer C Malspeis S Guan H. Interactions between genome-wide genetic factors and smoking influencing risk of systemic lupus erythematosus. Arthritis Rheumatol (2020) 72(11):1863–71. 10.1002/art.41414 PMC7722161 32969204

[350] Hahn J Cook NR Alexander EK Friedman S Walter J Bubes V. Vitamin d and marine omega 3 fatty acid supplementation and incident autoimmune disease: VITAL randomized controlled trial. BMJ (2022) 376:e066452. 35082139 10.1136/bmj-2021-066452 PMC8791065

[351] Ascherio A Munger KL White R Kochert K Simon KC Polman CH. Vitamin d as an early predictor of multiple sclerosis activity and progression. JAMA Neurol (2014) 71(3):306–14. 10.1001/jamaneurol.2013.5993 PMC4000029 24445558

[352] Feige J Moser T Bieler L Schwenker K Hauer L Sellner J. Vitamin d supplementation in multiple sclerosis: a critical analysis of potentials and threats. Nutrients (2020) 12(3). 10.3390/nu12030783 PMC7146466 32188044

[353] Lima GL Paupitz J Aikawa NE Takayama L Bonfa E Pereira RM. Vitamin d supplementation in adolescents and young adults with juvenile systemic lupus erythematosus for improvement in disease activity and fatigue scores: a randomized, double-blind, placebo-controlled trial. Arthritis Care Res (Hoboken) (2016) 68(1):91–8. 10.1002/acr.22621 25988278

[354] Franco AS Freitas TQ Bernardo WM Pereira RMR. Vitamin d supplementation and disease activity in patients with immune-mediated rheumatic diseases: a systematic review and meta-analysis. Med (Baltimore) (2017) 96(23):e7024. 10.1097/MD.0000000000007024 PMC5466211 28591033

[355] Stockton KA Kandiah DA Paratz JD Bennell KL. Fatigue, muscle strength and vitamin d status in women with systemic lupus erythematosus compared with healthy controls. Lupus (2012) 21(3):271–8. 10.1177/0961203311425530 22004972

[356] Ruiz-Irastorza G Gordo S Olivares N Egurbide MV Aguirre C. Changes in vitamin d levels in patients with systemic lupus erythematosus: effects on fatigue, disease activity, and damage. Arthritis Care Res (Hoboken) (2010) 62(8):1160–5. 10.1002/acr.20186 20235208

[357] Hayashi K Sada KE Asano Y Katayama Y Ohashi K Morishita M. Real-world data on vitamin d supplementation and its impacts in systemic lupus erythematosus: cross-sectional analysis of a lupus registry of nationwide institutions (LUNA). PloS One (2022) 17(6):e0270569. 35767524 10.1371/journal.pone.0270569 PMC9242469

[358] Magro R Saliba C Camilleri L Scerri C Borg AA. Vitamin d supplementation in systemic lupus erythematosus: relationship to disease activity, fatigue and the interferon signature gene expression. BMC Rheumatol (2021) 5(1):53. 34857051 10.1186/s41927-021-00223-1 PMC8641172

[359] Irfan SA Ali AA Shabbir N Altaf H Ahmed A Kunnath Thamara J. Effects of vitamin d on systemic lupus erythematosus disease activity and autoimmunity: a systematic review and meta-analysis. Cureus (2022) 14(6):e25896. 35844337 10.7759/cureus.25896 PMC9278795

[360] Alam M Amin SS Adil M Arif T Zahra FT Varshney I. Comparative study of efficacy of topical mometasone with calcipotriol versus mometasone alone in the treatment of alopecia areata. Int J Trichology (2019) 11(3):123–7. 10.4103/ijt.ijt_18_19 PMC6580810 31360041

[361] Molinelli E Campanati A Brisigotti V Sapigni C Paolinelli M Offidani A. Efficacy and safety of topical calcipotriol 0.005% versus topical clobetasol 0.05% in the management of alopecia areata: an intrasubject pilot study. Dermatol Ther (Heidelb) (2020) 10(3):515–21. doi: 10.1007/s13555-020-00379-7 PMC7211771 32342443

[362] Narang T Daroach M Kumaran MS. Efficacy and safety of topical calcipotriol in management of alopecia areata: a pilot study. Dermatol Ther (2017) 30(3). doi: 10.1111/dth.12464 28133875

[363] Mehta LR Dworkin RH Schwid SR. Polyunsaturated fatty acids and their potential therapeutic role in multiple sclerosis. Nat Clin Pract Neurol (2009) 5(2):82–92. doi: 10.1038/ncpneuro1009 19194388

[364] Maeshima E Liang XM Goda M Otani H Mune M. The efficacy of vitamin e against oxidative damage and autoantibody production in systemic lupus erythematosus: a preliminary study. Clin Rheumatol (2007) 26(3):401–4. doi: 10.1007/s10067-006-0477-x 17143589

[365] Abeer Shahba NEE. Abd-Allah fooda , samia El-dardiry , ayman wagih, omnia el-deeb, effect of nigella sativa and vitamin e on some oxidative / nitrosative biomarkers in systemic lupus erythematosus patients. Life Sci J (2015) 12(7):2015.

[366] Guan JZ Guan WP Maeda T. Vitamin e administration erases an enhanced oxidation in multiple sclerosis. Can J Physiol Pharmacol (2018) 96(11):1181–3. 10.1139/cjpp-2018-0246 30092167

[367] Kinoshita K Kishimoto K Shimazu H Nozaki Y Sugiyama M Ikoma S. Successful treatment with retinoids in patients with lupus nephritis. Am J Kidney Dis (2010) 55(2):344–7. 10.1053/j.ajkd.2009.06.012 19628316

[368] Vien CV Gonzalez-Cabello R Bodo I Gergely P. Effect of vitamin a treatment on the immune reactivity of patients with systemic lupus erythematosus. J Clin Lab Immunol (1988) 26(1):33–5. 3184159

[369] Bitarafan S Saboor-Yaraghi A Sahraian MA Soltani D Nafissi S Togha M. Effect of vitamin a supplementation on fatigue and depression in multiple sclerosis patients: a double-blind placebo-controlled clinical trial. Iran J Allergy Asthma Immunol (2016) 15(1):13–9. 26996107

[370] Mohammadzadeh Honarvar N Harirchian MH Abdolahi M Abedi E Bitarafan S Koohdani F. Retinyl palmitate supplementation modulates T-bet and interferon gamma gene expression in multiple sclerosis patients. J Mol Neurosci (2016) 59(3):360–5. 10.1007/s12031-016-0747-2 27122150

[371] Talpur R Vu J Bassett R Stevens V Duvic M. Phase I/II randomized bilateral half-head comparison of topical bexarotene 1% gel for alopecia areata. J Am Acad Dermatol (2009) 61(4):592.e1–9. doi: 10.1016/j.jaad.2009.02.037 19682769

[372] Costantini A Nappo A Pala MI Zappone A. High dose thiamine improves fatigue in multiple sclerosis. BMJ Case Rep (2013) 2013. doi: 10.1136/bcr-2013-009144 PMC3736110 23861280

[373] Cree BAC Cutter G Wolinsky JS Freedman MS Comi G Giovannoni G. Safety and efficacy of MD1003 (high-dose biotin) in patients with progressive multiple sclerosis (SPI2): a randomised, double-blind, placebo-controlled, phase 3 trial. Lancet Neurol (2020) 19(12):988–97. doi: 10.1016/S1474-4422(20)30347-1 33222767

[374] Camacho FM Garcia-Hernandez MJ. Zinc aspartate, biotin, and clobetasol propionate in the treatment of alopecia areata in childhood. Pediatr Dermatol (1999) 16(4):336–8. doi: 10.1111/j.1525-1470.1999.pdele65.x 10515774

[375] Salari S Khomand P Arasteh M Yousefzamani B Hassanzadeh K. Zinc sulphate: a reasonable choice for depression management in patients with multiple sclerosis: a randomized, double-blind, placebo-controlled clinical trial. Pharmacol Rep (2015) 67(3):606–9. doi: 10.1016/j.pharep.2015.01.002 25933976

[376] Lux-Battistelli C. Combination therapy with zinc gluconate and PUVA for alopecia areata totalis: an adjunctive but crucial role of zinc supplementation. Dermatol Ther (2015) 28(4):235–8. 10.1111/dth.12215 25754430

[377] Park H Kim CW Kim SS Park CW. The therapeutic effect and the changed serum zinc level after zinc supplementation in alopecia areata patients who had a low serum zinc level. Ann Dermatol (2009) 21(2):142–6. 10.5021/ad.2009.21.2.142 PMC2861201 20523772

[378] Ead RD. Oral zinc sulphate in alopacia areata-a double blind trial. Br J Dermatol (1981) 104(4):483–4. 10.1111/j.1365-2133.1981.tb15323.x 7016162

[379] Leiba A Amital H Gershwin ME Shoenfeld Y. Diet and lupus. Lupus (2001) 10(3):246–8. 10.1191/096120301674681790 11315362

[380] Wu JH Batist G. Glutathione and glutathione analogues; therapeutic potentials. Biochim Biophys Acta (2013) 1830(5):3350–3. doi: 10.1016/j.bbagen.2012.11.016 23201199

[381] Lai ZW Hanczko R Bonilla E Caza TN Clair B Bartos A. N-acetylcysteine reduces disease activity by blocking mammalian target of rapamycin in T cells from systemic lupus erythematosus patients: a randomized, double-blind, placebo-controlled trial. Arthritis Rheum (2012) 64(9):2937–46. 10.1002/art.34502 PMC3411859 22549432

[382] Valencia X Yarboro C Illei G Lipsky PE. Deficient CD4+CD25high T regulatory cell function in patients with active systemic lupus erythematosus. J Immunol (2007) 178(4):2579–88. 10.4049/jimmunol.178.4.2579 17277168

[383] Crispin JC Martinez A Alcocer-Varela J. Quantification of regulatory T cells in patients with systemic lupus erythematosus. J Autoimmun (2003) 21(3):273–6. 10.1016/s0896-8411(03)00121-5 14599852

[384] Perl A Hanczko R Lai ZW Oaks Z Kelly R Borsuk R. Comprehensive metabolome analyses reveal n-acetylcysteine-responsive accumulation of kynurenine in systemic lupus erythematosus: implications for activation of the mechanistic target of rapamycin. Metabolomics (2015) 11(5):1157–74. 10.1007/s11306-015-0772-0 PMC4559110 26366134

[385] Li M Gao W Ma J Zhu Y Li X. Early-stage lupus nephritis treated with n-acetylcysteine: a report of two cases. Exp Ther Med (2015) 10(2):689–92. 10.3892/etm.2015.2510 PMC4509375 26622376

[386] Schoeps VA Graves JS Stern WA Zhang L Nourbakhsh B Mowry EM. N-acetyl cysteine as a neuroprotective agent in progressive multiple sclerosis (NACPMS) trial: study protocol for a randomized, double-blind, placebo-controlled add-on phase 2 trial. Contemp Clin Trials (2022) 122:106941. 36182028 10.1016/j.cct.2022.106941

[387] Shahrampour S Heholt J Wang A Vedaei F Mohamed FB Alizadeh M. N-acetyl cysteine administration affects cerebral blood flow as measured by arterial spin labeling MRI in patients with multiple sclerosis. Heliyon (2021) 7(7):e07615. 34377857 10.1016/j.heliyon.2021.e07615 PMC8327674

[388] Carvalho AN Lim JL Nijland PG Witte ME Van Horssen J. Glutathione in multiple sclerosis: more than just an antioxidant? Mult Scler (2014) 20(11):1425–31. 10.1177/1352458514533400 24842957

[389] Sharquie KE Al-Obaidi HK. Onion juice (Allium cepa l.), a new topical treatment for alopecia areata. J Dermatol (2002) 29(6):343–6. doi: 10.1111/j.1346-8138.2002.tb00277.x 12126069

[390] Bianchini F Vainio H. Allium vegetables and organosulfur compounds: do they help prevent cancer? Environ Health Perspect (2001) 109(9):893–902. 11673117 10.1289/ehp.01109893 PMC1240438

[391] Bogaards JJ Verhagen H Willems MI van Poppel G van Bladeren PJ. Consumption of Brussels sprouts results in elevated alpha-class glutathione s-transferase levels in human blood plasma. Carcinogenesis (1994) 15(5):1073–5. 10.1093/carcin/15.5.1073 8200071

[392] Moore LE Brennan P Karami S Hung RJ Hsu C Boffetta P. Glutathione s-transferase polymorphisms, cruciferous vegetable intake and cancer risk in the central and Eastern European kidney cancer study. Carcinogenesis (2007) 28(9):1960–4. 10.1093/carcin/bgm151 17617661

[393] Bahadoran Z Mirmiran P Hosseinpanah F Hedayati M Hosseinpour-Niazi S. Broccoli sprouts reduce oxidative stress in type 2 diabetes: a randomized double-blind clinical trial. Eur J Clin Nutr (2011) 65(8):972–7. 10.1038/ejcn.2011.59 21559038

[394] Boyanapalli SS Kong AT. "Curcumin, the king of spices": epigenetic regulatory mechanisms in the prevention of cancer, neurological, and inflammatory diseases. Curr Pharmacol Rep (2015) 1(2):129–39. doi: 10.1007/s40495-015-0018-x PMC4596544 26457241

[395] Handono K Pratama MZ Endharti AT Kalim H. Treatment of low doses curcumin could modulate Th17/Treg balance specifically on CD4+ T cell cultures of systemic lupus erythematosus patients. Cent Eur J Immunol (2015) 40(4):461–9. 10.5114/ceji.2015.56970 PMC4737743 26862311

[396] Khajehdehi P Zanjaninejad B Aflaki E Nazarinia M Azad F Malekmakan L. Oral supplementation of turmeric decreases proteinuria, hematuria, and systolic blood pressure in patients suffering from relapsing or refractory lupus nephritis: a randomized and placebo-controlled study. J Ren Nutr (2012) 22(1):50–7. 10.1053/j.jrn.2011.03.002 21742514

[397] Dolati S Ahmadi M Aghebti-Maleki L Nikmaram A Marofi F Rikhtegar R. Nanocurcumin is a potential novel therapy for multiple sclerosis by influencing inflammatory mediators. Pharmacol Rep (2018) 70(6):1158–67. 10.1016/j.pharep.2018.05.008 30340096

[398] Dolati S Ahmadi M Rikhtegar R Babaloo Z Ayromlou H Aghebati-Maleki L. Changes in Th17 cells function after nanocurcumin use to treat multiple sclerosis. Int Immunopharmacol (2018) 61:74–81. 29852475 10.1016/j.intimp.2018.05.018

[399] Dolati S Babaloo Z Ayromlou H Ahmadi M Rikhtegar R Rostamzadeh D. Nanocurcumin improves regulatory T-cell frequency and function in patients with multiple sclerosis. J Neuroimmunol (2019) 327:15–21. 30683426 10.1016/j.jneuroim.2019.01.007

[400] Mao Y Xu Z Song J Xie Y Mei X Shi W. Efficacy of a mixed preparation containing piperine, capsaicin and curcumin in the treatment of alopecia areata. J Cosmet Dermatol (2022) 21(10):4510–4. 10.1111/jocd.14931 35318791

[401] Kucharski R Maleszka J Foret S Maleszka R. Nutritional control of reproductive status in honeybees via DNA methylation. Science (2008) 319(5871):1827–30. 10.1126/science.1153069 18339900

[402] Zahran AM Elsayh KI Saad K Eloseily EM Osman NS Alblihed MA. Effects of royal jelly supplementation on regulatory T cells in children with SLE. Food Nutr Res (2016) 60:32963. 27887663 10.3402/fnr.v60.32963 PMC5124115

[403] Farinotti M Vacchi L Simi S Di Pietrantonj C Brait L Filippini G. Dietary interventions for multiple sclerosis. Cochrane Database Syst Rev (2012) 12:CD004192. 23235605 10.1002/14651858.CD004192.pub3

[404] Jacobs DR Jr. Gross MD Tapsell LC. Food synergy: an operational concept for understanding nutrition. Am J Clin Nutr (2009) 89(5):1543S–8S. 10.3945/ajcn.2009.26736B PMC2731586 19279083

[405] Irish AK Erickson CM Wahls TL Snetselaar LG Darling WG. Randomized control trial evaluation of a modified paleolithic dietary intervention in the treatment of relapsing-remitting multiple sclerosis: a pilot study. Degener Neurol Neuromuscul Dis (2017) 7:1–18. 30050374 10.2147/DNND.S116949 PMC6053098

[406] Mediterranean Vs. high-Fermented-Food diet adherence on inflammation and disease activity in systemic lupus erythematosus University of Florida (2022).

[407] Harvey CJ. Combined diet and supplementation therapy resolves alopecia areata in a paediatric patient: a case study. Cureus (2020) 12(11):e11371. 33304703 10.7759/cureus.11371 PMC7721078

[408] Franzago M Santurbano D Vitacolonna E Stuppia L. Genes and diet in the prevention of chronic diseases in future generations. Int J Mol Sci (2020) 21(7). 10.3390/ijms21072633 PMC7178197 32290086

[409] Renauer PA Coit P Sawalha AH. The DNA methylation signature of human TCRalphabeta+CD4-CD8- double negative T cells reveals CG demethylation and a unique epigenetic architecture permissive to a broad stimulatory immune response. Clin Immunol (2015) 156(1):19–27. 25451162 10.1016/j.clim.2014.10.007 PMC4278938

[410] Lal G Zhang N van der Touw W Ding Y Ju W Bottinger EP. Epigenetic regulation of Foxp3 expression in regulatory T cells by DNA methylation. J Immunol (2009) 182(1):259–73. 10.4049/jimmunol.182.1.259 PMC3731994 19109157

[411] Zhao M Liang G Wu X Wang S Zhang P Su Y. Abnormal epigenetic modifications in peripheral blood mononuclear cells from patients with alopecia areata. Br J Dermatol (2012) 166(2):226–73. 10.1111/j.1365-2133.2011.10646.x 21936853

[412] Ma Q Caillier SJ Muzic S MSET University of California San Francisco Wilson MR Henry RG. Specific hypomethylation programs underpin b cell activation in early multiple sclerosis. Proc Natl Acad Sci U.S.A. (2021) 118(51). doi: 10.1073/pnas.2111920118 PMC8713784 34911760

[413] Roostaei T Klein HU Ma Y Felsky D Kivisakk P Connor SM. Proximal and distal effects of genetic susceptibility to multiple sclerosis on the T cell epigenome. Nat Commun (2021) 12(1):7078. 34873174 10.1038/s41467-021-27427-w PMC8648735

[414] Ray D Strickland FM Richardson BC. Oxidative stress and dietary micronutrient deficiencies contribute to overexpression of epigenetically regulated genes by lupus T cells. Clin Immunol (2018) 196:97–102. 29654844 10.1016/j.clim.2018.04.003 PMC6181796

[415] Wu T Xie C Han J Ye Y Weiel J Li Q. Metabolic disturbances associated with systemic lupus erythematosus. PloS One (2012) 7(6):e37210. 22723834 10.1371/journal.pone.0037210 PMC3378560

[416] Singhal NK Li S Arning E Alkhayer K Clements R Sarcyk Z. Changes in methionine metabolism and histone H3 trimethylation are linked to mitochondrial defects in multiple sclerosis. J Neurosci (2015) 35(45):15170–86. 10.1523/JNEUROSCI.4349-14.2015 PMC6605362 26558787

[417] Singhal NK Freeman E Arning E Wasek B Clements R Sheppard C. Dysregulation of methionine metabolism in multiple sclerosis. Neurochem Int (2018) 112:1–4. 29080803 10.1016/j.neuint.2017.10.011

[418] Gardner LA Desiderio DM Groover CJ Hartzes A Yates CR Zucker-Levin AR. LC-MS/MS identification of the one-carbon cycle metabolites in human plasma. Electrophoresis (2013) 34(11):1710–6. 10.1002/elps.201200536 23417555

[419] David LA Maurice CF Carmody RN Gootenberg DB Button JE Wolfe BE. Diet rapidly and reproducibly alters the human gut microbiome. Nature (2014) 505(7484):559–63. 10.1038/nature12820 PMC3957428 24336217

[420] Bjornevik K Cortese M Healy BC Kuhle J Mina MJ Leng Y. Longitudinal analysis reveals high prevalence of Epstein-Barr virus associated with multiple sclerosis. Science (2022) 375(6578):296–301. 35025605 10.1126/science.abj8222

[421] Manzel A Muller DN Hafler DA Erdman SE Linker RA Kleinewietfeld M. Role of "Western diet" in inflammatory autoimmune diseases. Curr Allergy Asthma Rep (2014) 14(1):404. 24338487 10.1007/s11882-013-0404-6 PMC4034518

[422] De Jager PL Hacohen N Mathis D Regev A Stranger BE Benoist C. ImmVar project: insights and design considerations for future studies of "healthy" immune variation. Semin Immunol (2015) 27(1):51–7. 10.1016/j.smim.2015.03.003 25819567

